# Meta-analysis showing that ERCC1 polymorphism is predictive of osteosarcoma prognosis

**DOI:** 10.18632/oncotarget.19370

**Published:** 2017-07-19

**Authors:** Xueyong Liu, Zhan Zhang, Chunbo Deng, Yihao Tian, Xun Ma

**Affiliations:** ^1^ Department of Spine and Joint Surgery, Shengjing Hospital of China Medical University, Shenyang, China; ^2^ Department of Spine Surgery, Shengjing Hospital of China Medical University, Shenyang, China; ^3^ Department of Orthopedics, Fengtian Hospital of Shenyang Medical College, Shenyang, China

**Keywords:** ERCC, meta-analysis, polymorphism, osteosarcoma, prognosis

## Abstract

To investigate correlations between excision repair cross-complementation group 1 (ERCC1) and 2 (ERCC2) polymorphisms and osteosarcoma prognosis, we conducted a meta-analysis of studies published through October 2016. Studies were identified in the PubMed, ScienceDirect, Springer, and Web of Science databases using preferred reporting items for systematic reviews and meta-analyses (PRISMA). Odds ratios (ORs) or hazard ratios (HRs) and their 95% confidence intervals (CIs) for overall survival (OS), tumor response (TR), and event-free survival (EFS) were estimated. Our meta-analysis included eleven studies in which four SNPs (ERCC1 rs11615 and rs3212986, ERCC2 rs13181 and rs1799793) reportedly associated with osteosarcoma prognosis were investigated. Each of these studies scored > 6 on the Newcastle-Ottawa Scale (NOS). We found that only one SNP, ERCC1 rs11615, correlated with improved OS and TR. The HR of T vs. C for OS was 1.455 (T/C, 95% CI = 1.151–1.839, *P =* 0.002, I^2^ = 37.80%). The OR of T vs. C for good TR was 0.554 (T/C, 95% CI = 0.437–0.702, *P* < 0.001, I^2^ = 0%). Few significant outcome was observed in subgroup analyses stratified based on study characteristics with adjustments for potential confounders. Our results suggest that ERCC1 rs11615 CC is associated with a better clinical outcome. This suggests rs11615 may be a useful genetic marker for predicting osteosarcoma prognosis.

## INTRODUCTION

Osteosarcoma is one of the most common and aggressive malignant bone tumors, primarily occurring during adolescent growth and in the elderly [1]. Osteosarcoma incidence in adolescents is relatively consistent globally and ranges from 3–4.5 cases per million persons per year [2]. Although osteosarcoma treatment options have improved, patient prognosis remains poor [3]. Multiple genes, including *VEGF*, *GRM4*, *GSTP1*, *ABCB1*, and key enzymes of the DNA repair system, have been identified as osteosarcoma biomarkers that may predict patient susceptibility and prognosis [4–6].

DNA repair is critical for maintaining DNA stability and integrity, and cell function. The nucleotide excision repair (NER) pathway is responsible for recognizing and excising DNA lesions [7]. Excision repair cross-complementation group 1 (ERCC1) and 2 (ERCC2), located in 19q13.3, are key rate-limiting enzymes in the NER process [8]. ERCC1 and xeroderma pigmentosum group F (XPF) form a heterodimer to catalyze 5′–3′ incisions, while ERCC2 exhibits ATP-dependent DNA helicase activity, inducing apoptosis and basal transcription. Therefore, ERCC polymorphisms may impact DNA repair and cancer development and progression [7, 9]. Several ERCC single nucleotide polymorphisms (SNPs) associated with osteosarcoma prognosis have been reported, including ERCC1 rs11615 (Asn118Asn) and rs3212986 (Gln504Lys), and ERCC2 rs13181 (Lys751Gln) and rs1799793 (Asp312Asn) [10–21]. We conducted a systematic review and meta-analysis of ERCC1 and 2 polymorphisms to identify any direct correlations between such polymorphisms and osteosarcoma patient prognosis.

## RESULTS

### Reference search

**Figure 1:**
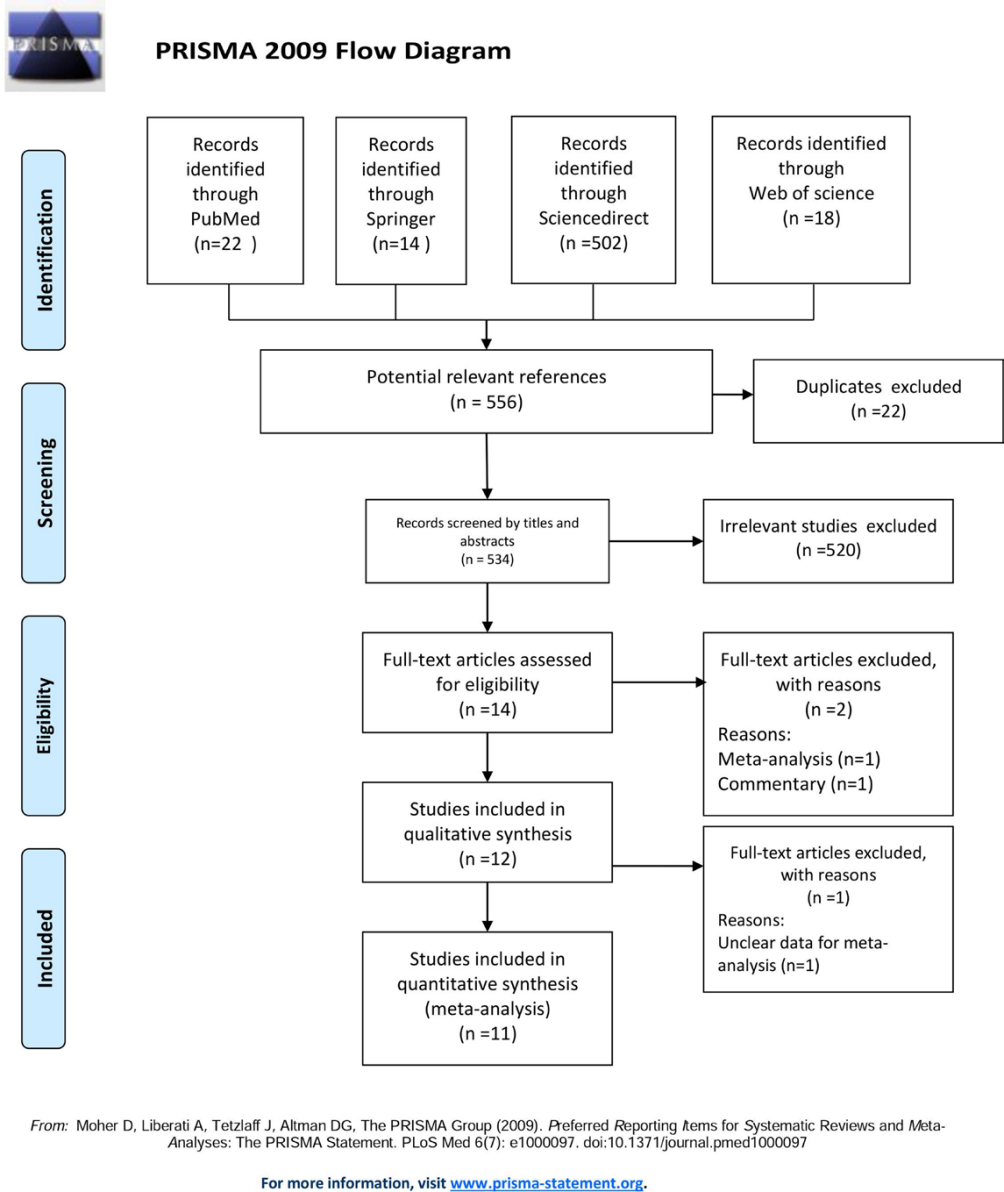
PRISMA 2009 flow diagram

The combined search yielded 556 potentially relevant references. References were screened by title, abstract, and full-text (Figure 1). We found 14 studies regarding ERCC polymorphisms, including one meta-analysis and one commentary, both of which were excluded. One study [19] was excluded due to lack of detailed data. Eleven studies met our criteria and were included in the meta-analysis.

### Data extraction and reference assessment

Data extracted from the 11 studies are shown in Supplementary Table 1, and primary extracted data are shown in Supplementary Table 2. We encountered several challenges during data extraction. In studies by Yang, *et al.* and Hao, *et al.*, rs13181 referred to G/A, but was an A/C SNP of ERCC2 according to PubMed dbSNP, websites of dbSNP of the studied SNPs were provided in Supplementary Table 16. Similarly, in the Hao, *et al.* study, rs1799793 referred to G/T, but was a G/A SNP of ERCC2 according to PubMed dbSNP. We therefore excluded these rs13181 and rs1799793 studies. Additionally, two rs11615 genotype formats were referred to as T/C and C/T, which was also the case in previous rs11615-related studies, and this issue remains unsettled.

In these 11 studies, we were able to examine tumor response (TR), overall survival (OS), and event-free survival (EFS). TR was categorized as either good (GTR) or poor (PTR). GTR was defined as the extent of tumor necrosis > 90% in histology or response (complete response (CR) and partial response (PR)) as per Response Evaluation Criteria in Solid Tumors (RECIST) criteria. Tumor necrosis < 90% or non-response (stable disease (SD) and progressive disease (PD)) as per RECIST criteria was defined as PTR. If the histological findings and RECIST criteria showed good homogeneity, subgroup analysis would be performed if necessary. RECIST criteria was employed to assess TR only in the studies by Yang, *et al.* and Liu, *et al.* Primary and adjusted PTR were only available in the study by Carolina, *et al.* Therefore, we used crude ORs from other studies so that this study could be included. OS was usually obtained from diagnosis until death by any cause or last follow-up. EFS was usually defined as the period before first relapse and after tumor diagnosis.

Newcastle-Ottawa Scale (NOS) assessments are shown in Table 1. With the aim of evaluating studies more precisely, we added additional clauses to some items. All studies received scores ≥ 6.

**Table 1:** Outcomes of reference assessment (Newcastle-Ottawa Scale)

First author	Selection				Comparability		Exposure			Total scores
	Is the case definition adequate? #	Representativeness of the cases $	Selection of Controls	Definition of Controls	Study controls for select the most important factor^@^	Study controls for any additional factor^&^	Ascertainment of exposure*	Same method of ascertainment for cases and controls	Non- Response rate^†^	
D Caronia	☆	-	☆	☆	-	☆	☆	☆	☆	7
Katja Goričar	☆	-	☆	☆	☆	☆	☆	☆	☆	8
M.J. Wang	☆	-	☆	☆	☆	-	-	☆	☆	6
Paola Biason	☆	☆	☆	☆	-	☆	-	☆	☆	7
Q. Zhang	☆	-	☆	☆	☆	☆	-	☆	☆	7
Ting Hao	☆	-	☆	☆	☆	-	-	☆	☆	6
Wei-Ping Ji	☆	-	☆	☆	☆	☆	-	☆	-	6
Li-Min Yang	☆	-	☆	☆	☆	☆	-	☆	☆	7
Yongjian Sun	☆	☆	☆	☆	☆	☆	-	☆	☆	8
Z.F. Liu	☆	-	☆	☆	☆	☆	-	☆	☆	7
Z.H. Cao	☆	-	☆	☆	☆	☆	-	☆	☆	7

#: Study with clear diagnosis from clinical, histopathologically evidence was assigned one star.

$: Study with metastasis occurred at diagnosis might lead to a choose bias and was not obtain one star.

@: A study with main confounders like age and gender not adjusted was not assigned one star.

&: A study with other important confounders like tumor-related characteristics not adjusted was not assigned one star.

*Besides primary assessment, a study with a clear M-FU > 5 year was assigned one star.

†A study with a follow-up rate > 75% and equal non-response rate between groups was assigned one star.

### Statistical analysis

Statistical analysis was performed after study assessment and primary data extraction. This study included 1834 osteosarcoma cases. We were unable to merge OS/TR/EFS data extracted from the study by Hattinger, *et al.* into the pooled analysis, and these data were therefore excluded. Hattinger, *et al.* found that ERCC1 rs11615 and rs3212986 were not associated with osteosarcoma EFS. Meta-analysis results are shown in Supplementary Table 3, and rs11615 subgroup analysis outcomes are shown in Supplementary Table 4.

### Overall survival

**Figure 2:**
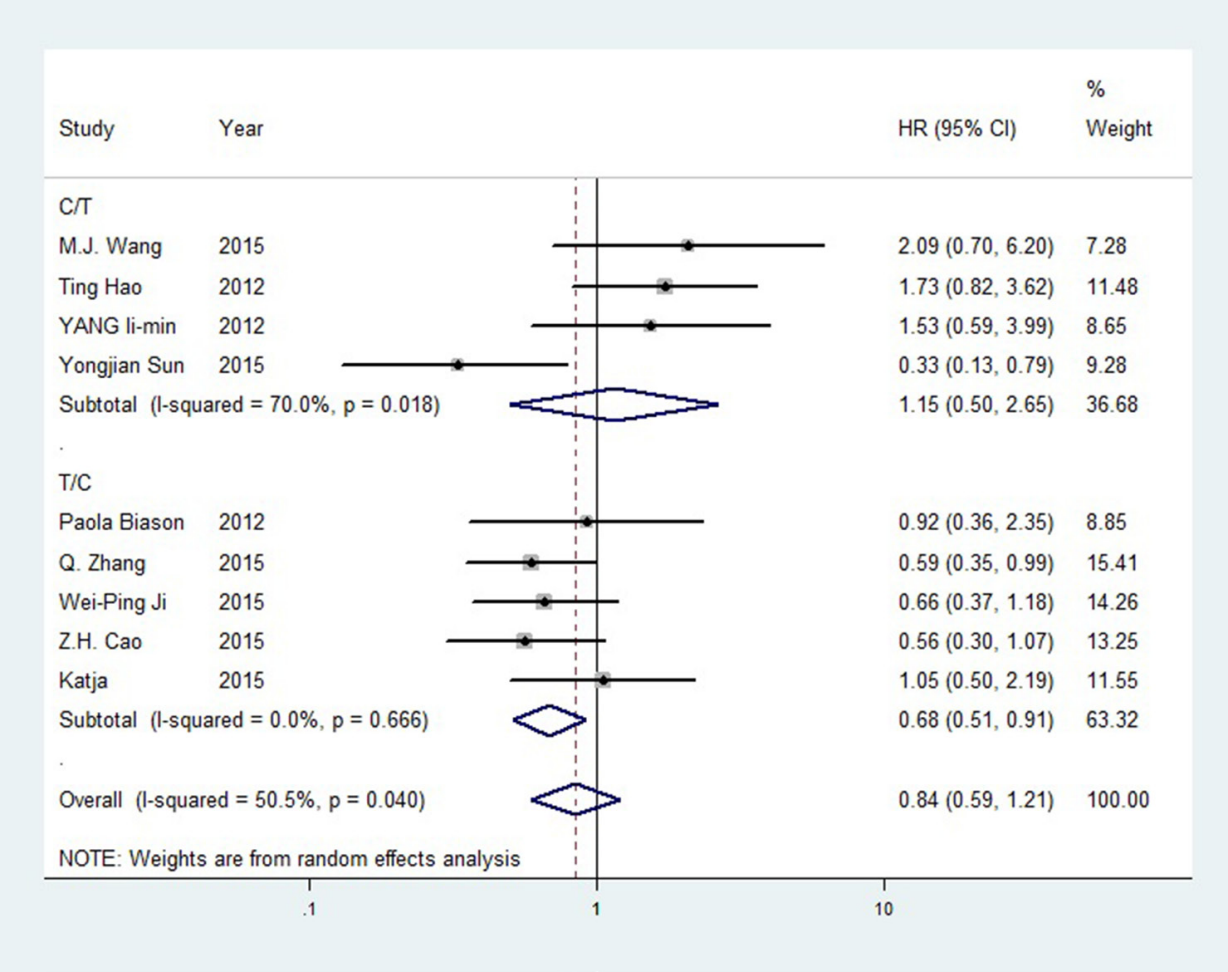
Forest plot of rs11615 OS (TC+CC vs. TT)

For ERCC1 rs11615, no correlation was observed in the pooled outcomes, but high heterogeneity was detected, which may have resulted from the unclear genotype designations discussed above. We observed a strong positive correlation between the C allele and OS in the T/C subgroup. The hazard ratio (HR) of the T vs. C OS model was 1.455 (T/C, 95%CI = 1.151–1.839, *P* = 0.002, I^2^ = 37.80%). For the ERCC2 rs1799793 G allele and the rs13181 A allele, we observed slightly positive OS trends, but these were not significant. In rs3212986, no significant outcome was observed. OS forest plot for rs11615 subgroup analysis is shown in Figure 2, forest plots for other SNPs are available in Supplementary Figures 4–6.

### Tumor response

**Figure 3:**
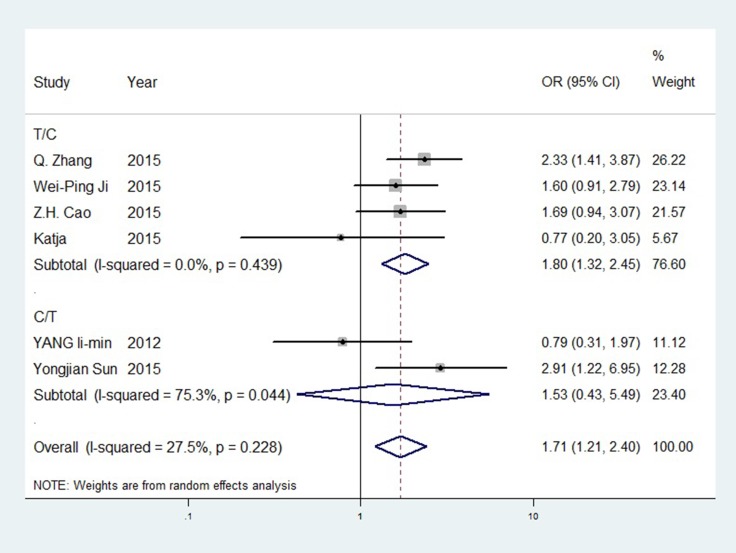
Forest plot of rs11615 GTR (T vs. C)

For ERCC1 rs11615, the C allele was more significant in the T/C subgroup than overall outcomes. The odds ratio (OR) for CC vs. TT was 2.659 (95%CI = 1.554–4.548, *P* < 0.001, I^2^ = 0.00%). The C/T subgroup showed no significance. For the ERCC2 SNPs, rs1799793 and rs13181, no significance was observed before sensitivity analysis. The rs1799793 A allele might have a better TR than the G allele, and the rs13181 C allele had a better TR than the A allele. No significance was found in rs3212986. GTR forest plot for rs11615 is shown in Figure 3, is revised as forest plots for other SNPs are available in Supplementary Figures 1–3. Differences between PTR and GTR outcomes were not significant.

### Event-free survival

All models for the four SNPs about EFS information included ≤ 3 studies. High heterogeneity was observed and could not be eliminated, and no significant outcome differences were found before or after sensitivity analysis.

### Subgroup analysis

OS and TR subgroup analyses were performed according to the indexes TR evaluation method, HWE, race, treatment and adjustment of confounding factors of tumor-related variables. From subgroup analyses, no significant difference was observed from overall outcomes. We observed that two assessments for TR, histological assessment and RECIST criteria, could be seen as in a good homogeneity. In subgroup analysis of race, only study by Biason, *et al.* was included in Caucasian subgroup, thus power of this estimation was limited and further study was needed. Results of subgroup analysis were available in Supplementary Tables 5–13.

### Heterogeneity

**Figure 4:**
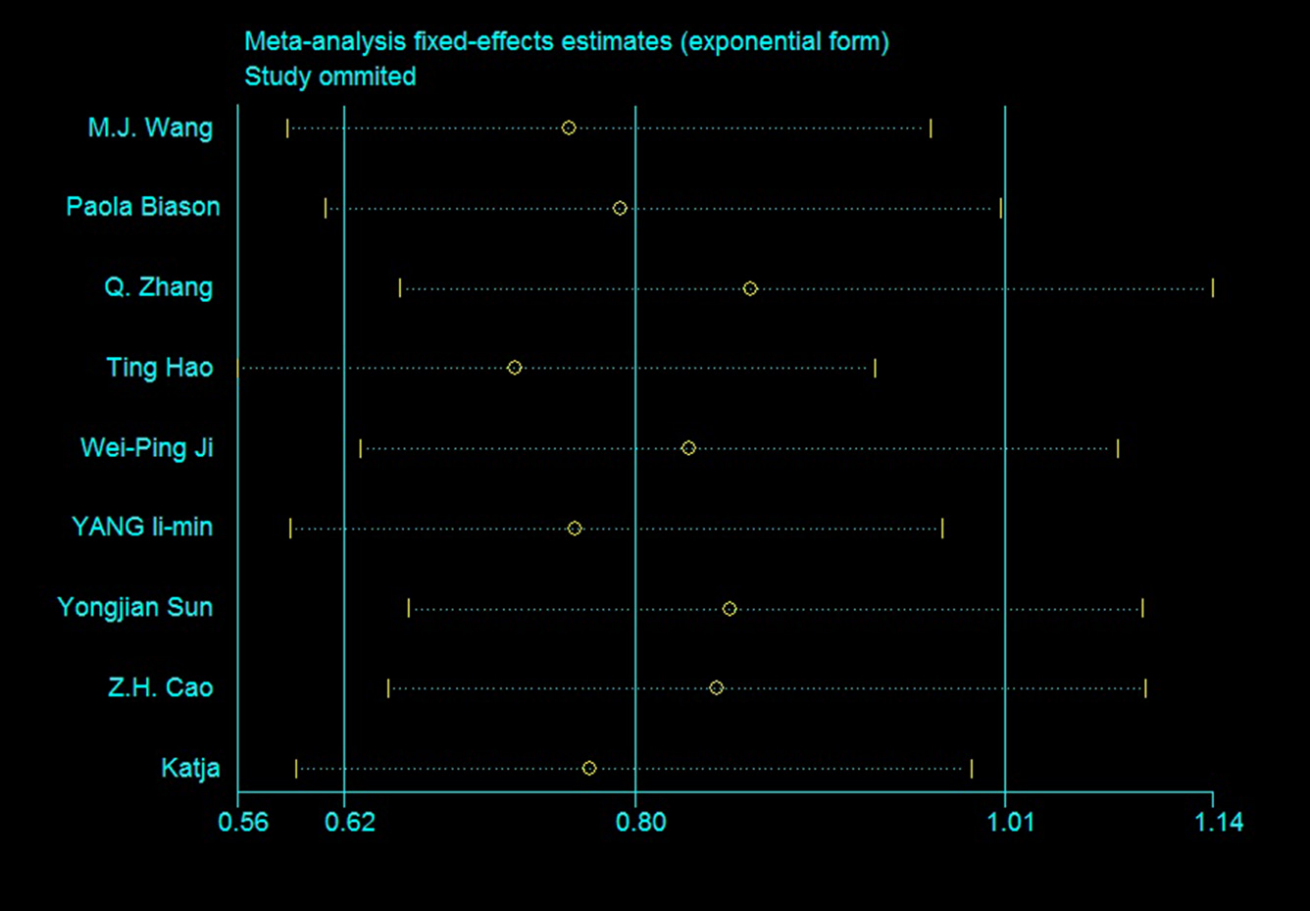
Sensitivity analysis of rs11615 OS (TC+CC vs. TT)

No models exhibited high heterogeneity and most of the heterogeneity could be eliminated. In 12+22 vs. 11 and 1 vs. 2, heterogeneity likely stemmed from crude ORs merged with adjusted ORs, or from unclear genotype formats like ERCC1 rs11615. However heterogeneity was mainly concentrated in several studies, especially that of Sun, *et al.* We were unable to determine why these studies provided such large heterogeneity. Sensitivity analyses for the rs11615 OS is shown in Figure 4, plots for other SNPs are available in Supplementary Figures 10–12.

### Publication bias

**Figure 5:**
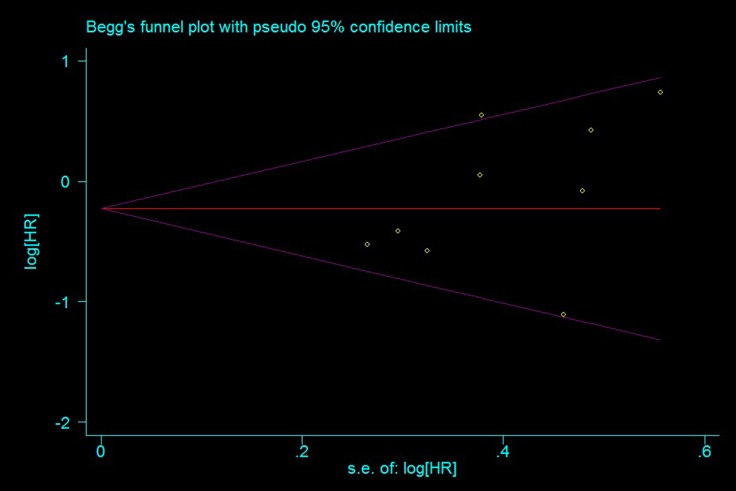
Funnel plot for publication bias estimation, rs11615 OS (TC+CC vs. TT)

Begg’s tests did not show significance for any SNPs with any outcomes, and Egger’s test found significance for TR only in some models (Figure 5 and Supplementary Figures 7–9). This may have been because TR was not estimated in some studies, and thus sample size was reduced.

## DISCUSSION

ERCCs are key enzymes of the NER system, which monitors and repairs DNA damage caused by endogenous and exogenous factors, and are vital in maintaining genome stability and cellular functions [25]. Some of ERCC polymorphisms negatively impact NER system function, promoting tumor development and progression [26]. We conducted an updated systematic review and meta-analysis to investigate associations between ERCC1 and 2 polymorphisms and osteosarcoma prognosis. Of the four SNPs studied, only ERCC1 rs11615 was associated with improved patient OS and TR. No significant outcomes were associated with ERCC2 rs13181 or rs1799793, or ERCC1 rs3212986.

A 2014 meta-analysis of the association between ERCC polymorphisms and osteosarcoma prognosis [27] appears to have had several limitations [28]. The analysis did not provide NOS assessments or HWE test outcomes, and sensitivity analysis and publication bias tests were not performed as well. Additionally, crude ORs/HRs were used in primary outcomes, rather than adjusted ORs/HRs. Finally, two rs11615 genotype formats, T/C or C/T, were not in accordance in included studies, and might have lead to unclear estimations. Based on the recent studies, we attempted to more precisely clarify associations of the tested ERCC polymorphisms with osteosarcoma prognosis.

Metastasis and TR are two of the most important osteosarcoma patient prognosis predictors [29], and current osteosarcoma therapies include surgery and primary and adjuvant chemotherapy. Success of the first-line methotrexate, doxorubicin/adriamycin, and cisplatin (MAP) chemotherapy regimen is limited by osteosarcoma heterogeneity [30] and tumor resistance to platinum-based cisplatin. Cisplatin is a widely-used first-line anti-cancer chemotherapeutic. Platinum agents adduct to DNA, leading to cell death. Platinum resistance is generally defined by tumor recurrence within one month after the last drug administration [31]. Cisplatin resistance may result from increased DNA repair capacity, changes in cisplatin cellular accumulation, and drug inactivation. High-level ERCC1 may remove the platinum-DNA adduct, leading to cisplatin resistance through increased DNA repair capacity. Some ERCC1 SNPs were confirmed as potential cisplatin resistance biomarkers [31]. ERCC SNPs and expression variations may alter DNA repair, thus impacting cell sensitivity to platinum agents [32]. ERCC1 mRNA increased by 6-fold in human ovarian cancer cells exposed to cisplatin, possibly due to increased expression of transactivating factors and c-Jun phosphorylation [33]. Nucleotides -415 to -220 and -220 to -110, upstream of the ERCC1 initiation site, are critical to cisplatin-induced ERCC1 overexpression and promoter activity, respectively [34].

These findings suggest that ERCC1 is an attractive target for reversing cisplatin resistance. UCN-01 (7-hydroxylstaurosporine) is a protein kinase C (PKC) inhibitor that inhibits the ERCC1/xeroderma pigmentosum group A (XPA) interaction, and may reduce ERCC1-induced cisplatin resistance. However, UCN-01 was not developed with the specific aim of reversing cisplatin resistance, and remains in Phase I clinical trials [35–36]. Li, *et al.* observed that siRNA-mediated ERCC1 inhibition promoted cisplatin sensitivity and apoptosis in gastric cancer [37]. Zhang, *et al.* found that Hsp90 inhibitors downregulated ERCC1 and reversed cisplatin resistance in ovarian cancer cells [38]. Similarly, several studies associated increased ERCC2 expression with cisplatin resistance. Zhao, *et al.* reported cisplatin sensitivity and apoptosis following ERCC2 downregulation via miR-770-5p in ovarian cancer cells [39].

ERCC1 overexpression indicated worse survival in osteosarcoma [30, 40] and non-small-cell lung cancer (NSCLC) patients [41–43] receiving cisplatin-based chemotherapy. Similarly, improved patient survival and platinum-based chemotherapy TR were observed in cholangiocarcinoma, cervical squamous cell carcinoma, and other tumors with lower ERCC1 levels [44–51]. However, while ERCC1 may predict chemotherapy sensitivity in these cancers, low ERCC1 expression was also associated with higher tumorigenesis risk [52]. ERCC1 prognosis prediction may also not be independent, and depend on whether or not a patient receives platinum-based chemotherapeutics [53, 54], and may be somehow limited [55]. ERCC2 expression has not been heavily investigated with respect to patient prognosis and platinum-based chemotherapy TR. Ye, *et al.* reported ERCC2 overexpression in cervical squamous cell carcinoma compared to normal cervical tissue [56], but Vogel, *et al.* found no ERCC2 expression changes in lung cancer-derived lymphocytes [57].

ERCC1 SNP rs11615 in codon 118 reduces ERCC1 transcription [58] and is correlated with patient survival and platinum-based chemotherapy TR in multiple tumor types [59–62]. However, a discrepancy in this SNP between T/C or C/T, possibly as a result of global allele variations, may have lead to inaccurate findings [31, 32]. SNP rs3212986 in the ERCC1 3′ UTR is thought to decrease ERCC1 mRNA stability. rs3212986 was associated with better prognosis in NSCLC [61] and T4 stage breast cancers [62] treated with platinum-based chemotherapy, and A allele carriers may be more likely to develop glioma [63–65]. The two studied ERCC2 SNPs, rs13181 and rs1799793, were associated with lower DNA repair capacity and increased DNA aberrations [65–66]. Our finding that rs13181 was not associated with tumor risk or prognosis agreed with previous analyses, although some groups associated the C allele with higher cancer risk and worse prognosis [60, 65, 67–75]. This allele was also associated with radiotherapy toxicity in NSCLC [70–71]. SNP rs1799793 was associated with higher risk of bladder cancer and gastric carcinoma [72–73] and poor OS of NSCLC [58], but had no apparent significance in glioma or non-Hodgkin’s lymphoma [67, 74–76]. Therefore, these two SNPs were not considered as prognostic indicators. However, we observed a slightly positive correlation between these two SNPs and osteosarcoma OS and chemotherapy TR, with some correlations significant after sensitivity analysis. Further studies are needed to identify the value of these two SNPs in targeted therapies and prognosis prediction.

Our study had limitations. First, numerous factors were involved in tumor prognosis. Single SNPs are unlikely to be major factors affecting tumor prognosis. Gene-environment and gene-gene interactions, and the combined effects of multiple SNPs in several genes are more effective outcome predictors than single SNPs alone [77–78]. However, we found no relevant gene interaction data in the included studies. Second, primary data was not available in some models. Thus, adjusted ORs were merged with crude ORs, and heterogeneity under such circumstances was high. We were unable to determine the sources of heterogeneity for some studies, such as that of Sun, *et al.* In addition, in ERCC1 rs11615, discrepancies between T/C and C/T remain unsettled [31]. While rs11615 T/C was correct according to PubMed dbSNP, we could not confirm this with high confidence from our study. rs11615 might reduce levels of ERCC1 mRNA [31], but it was unclear that which allele switched the mRNA levels. In our analysis, T/C and C/T study outcomes were obviously contradictory, with high heterogeneity. We performed a subgroup analysis based on T/C and C/T to reduce heterogeneity, but our sample size was somewhat limited. Further analyses are necessary to rectify this confusing issue.

In conclusion, our meta-analysis indicated that ERCC1 rs11615 is associated with improved osteosarcoma prognosis. Additional studies with larger sample sizes are needed to more precisely estimate the correlation between ERCC polymorphisms and osteosarcoma prognosis.

## MATERIALS AND METHODS

We conducted this meta-analysis based on the preferred reporting items for systematic reviews and meta-analyses (PRISMA) statement [22]. PRISMA checklist was available in Supplementary Table 15.

### Literature search strategy

We comprehensively searched for potential references from the PubMed, ScienceDirect, Springer, and Web of Science databases using key words such as “ERCC,” “osteosarcoma,” “outcome,” etc. through October, 2016. Our detailed literature search strategy is provided in the Supplementary Table 14.

### Study selection

Studies included in our analysis met the following criteria: (1) studies were limited to the published research concerning osteosarcoma prognosis and ERCC polymorphisms; (2) patients in the original studies must have been diagnosed with osteosarcoma via imaging, pathology, or the latest clinical diagnostic criteria, and genotyping was performed using valid molecular techniques; and (3) detailed patient data and the number of participants with distinct genotypes were published in the studies so that the adjusted or crude OR/HR value could be calculated. If the same or overlapping data appeared in multiple studies, the study with the largest sample size or most recent publication date was included in the meta-analysis.

### Data extraction

Data were extracted by two investigators independently and double-checked by a third investigator. Inconsistent data were addressed by open discussion and consensus was achieved via input from a senior investigator.

### Reference quality assessment

Two investigators conducted literature quality assessments of the included studies according to the NOS developed for case-control studies [23], and the star system was employed. The NOS included three domains: case and control selection, comparability, and exposure, eight items with nine stars in total. It was considered as a high quality (or low-bias risk) study if total stars achieved six to nine. Four to five stars would be considered as having intermediate-bias risk and one to three stars may have high-bias risk.

### Statistical analysis

Stata 11.0 software was used to perform this meta-analysis. A Hardy-Weinberg equilibrium (HWE) test was performed using extracted data, and *P* < 0.05 was considered a significant imbalance. Adjusted HRs/ORs from confounders (age, metastasis, etc.) in every model were used in our meta-analysis; otherwise, crude HRs/ORs calculated by Revman 5.3 software or given were used. Pooled data had low heterogeneity if *P* > 0.1 and I^2^ < 50%. In these cases, a fixed effects model was used; otherwise, a random effects model was used. Statistical analysis of pooled data was performed using models as follows: 12 versus 11, 22 versus 11, 12 versus 22, 12+22 versus 11, 1 versus 2 (1 represented the wild allele and 2 represented the mutated allele).

Tumor-related indexes, including TR, OS, and EFS, were assessed if the number of studies containing usable data was greater than 3. Statistical analyses were two-sided and *P* < 0.05 was considered significant. Z-score was evaluated by the *P value* of two-sided *u-test* for overall effect estimation.

### Subgroup analysis

Subgroup analyses were conducted based on study characteristics, to investigate sources of heterogeneity and potential correlations. As per the Savage, *et al.* study [2], the following variables were assessed in subgroup analyses: TR evaluation method, HWE, race, treatment and adjustment of confounding factors of tumor-related variables.

### Heterogeneity and sensitivity analyses

Sensitivity analysis was performed to investigate sources of heterogeneity and estimate the impacts of excluded studies on pooled outcomes. We also performed sensitivity analyses by excluding one study at a time to explore whether results were strongly influenced by a specific study. If the I^2^ value decreased (or even reached 0%), the removed study was considered a source of heterogeneity. Meta-analysis outcomes before and after the study was removed were then compared. If heterogeneity could not be eliminated, maximum or minimum extremes were excluded to achieve a more conservative estimation. Sensitivity analysis was not performed when the number of studies was < 3.

### Publication bias

Egger’s linear regression and Begg’s rank correlation tests were performed to evaluate potential publication bias [24]. *P* < 0.05 designated a significant publication bias. Funnel plots were used to visually evaluate publication bias.

## SUPPLEMENTARY MATERIALS FIGURES AND TABLES


































