# Supplementary material for: Meta-analysis showing that ERCC1 polymorphism is predictive of osteosarcoma prognosis
Source: Oncotarget. 2017 Jul 19;8(37):62769–79. doi: 10.18632/oncotarget.19370 (PMC5617547; doi:10.18632/oncotarget.19370)
Supplement: Supplementary file 1 [file oncotarget-08-62769-s001.pdf]

Meta-analysis showing that ERCC1 polymorphism is predictive of osteosarcoma prognosis

SUPPLEMENTARY MATERIALS

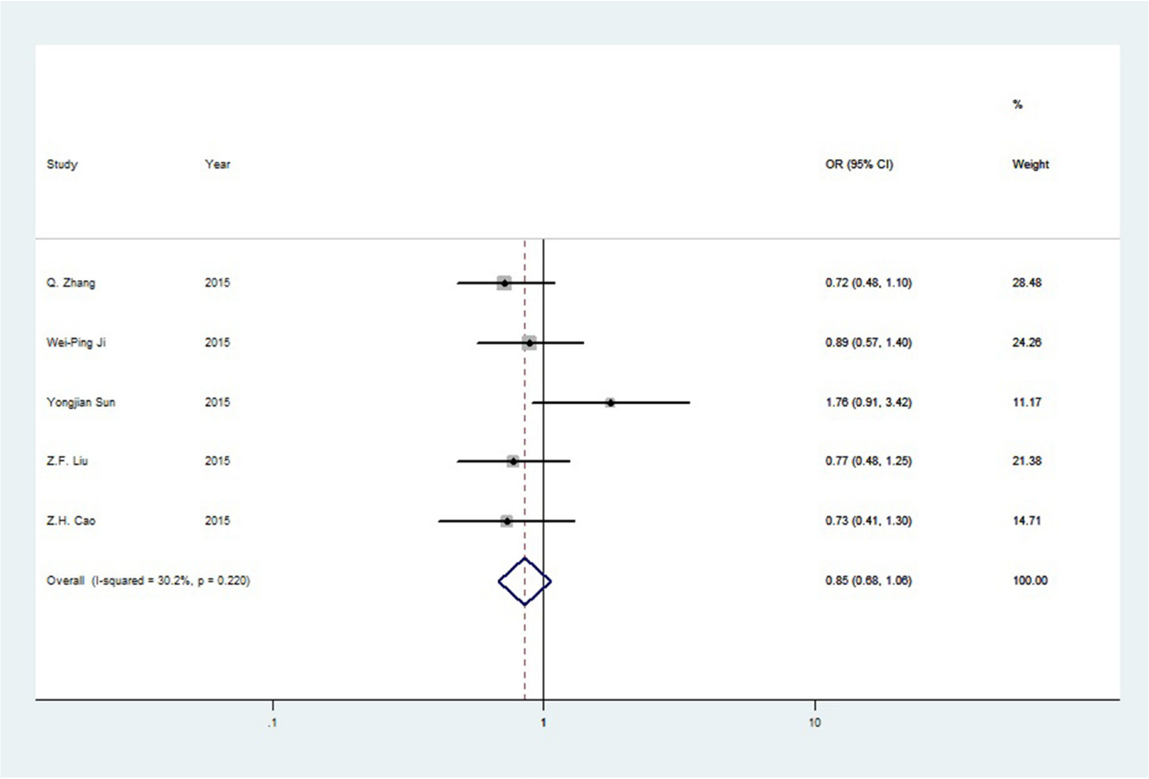

Supplementary Figure 1: Forest plot for GTR of rs13181 A vs. C.

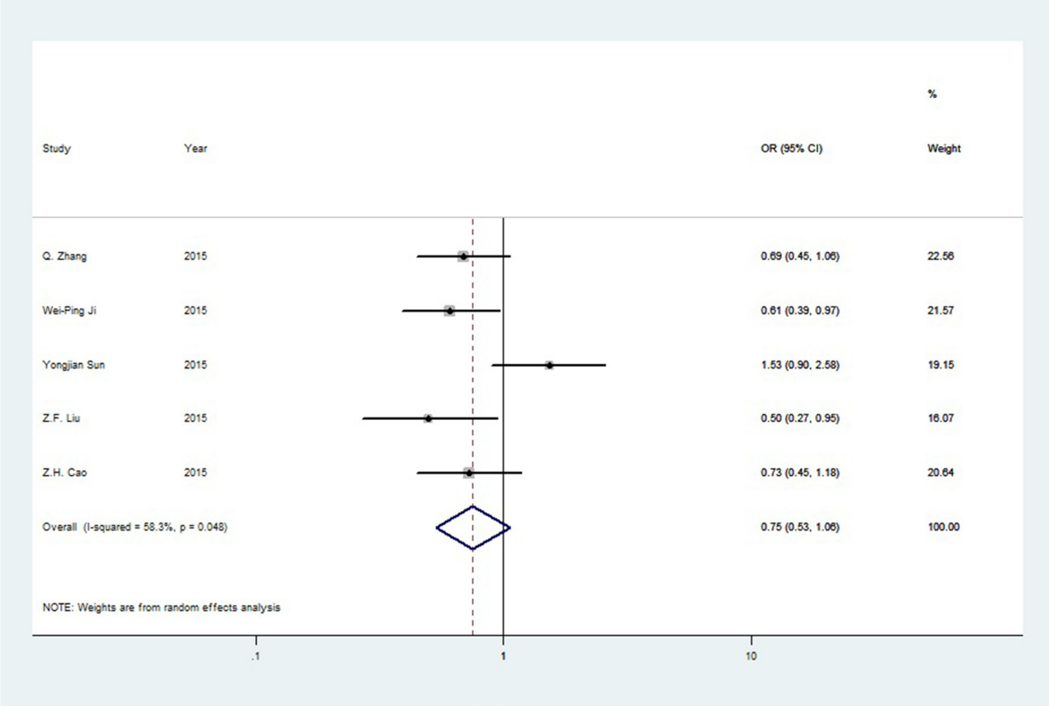

Supplementary Figure 2: Forest plot for GTR of rs1799793 G vs. A.

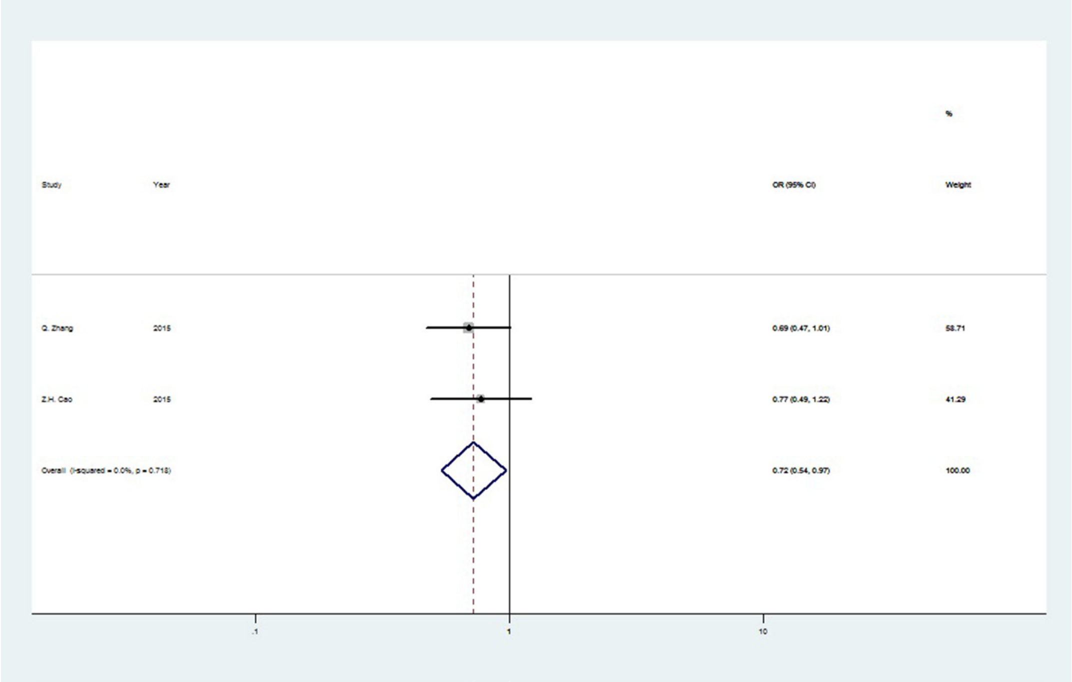

Supplementary Figure 3: Forest plot for GTR of rs3212986 C vs. A.

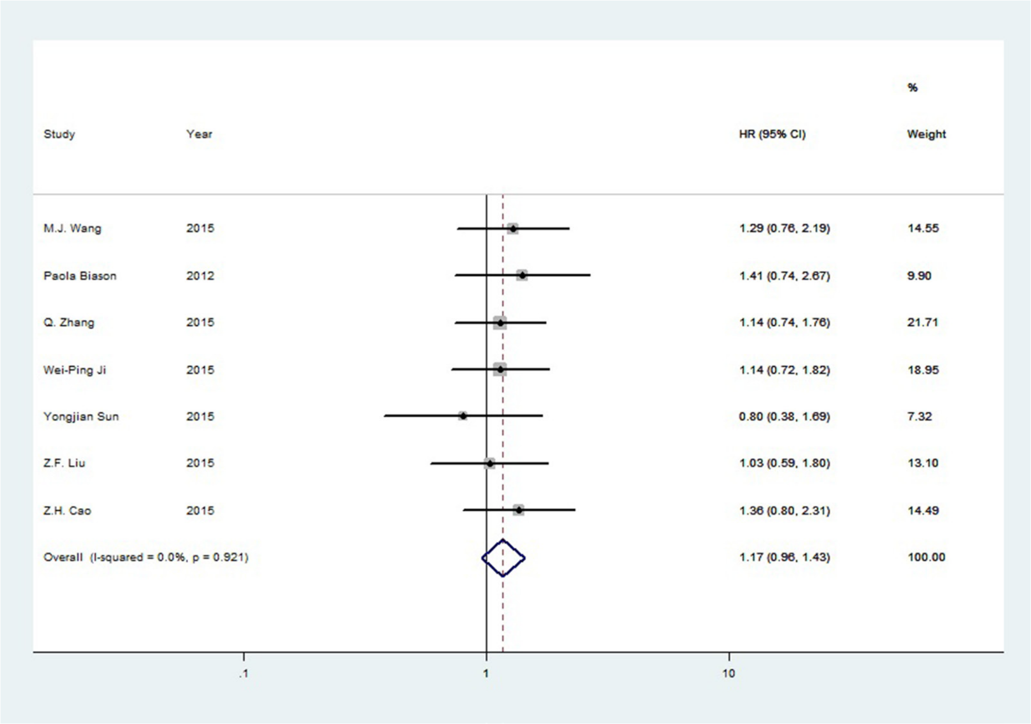

Supplementary Figure 4: Forest plot for OS of rs13181 A vs. C.

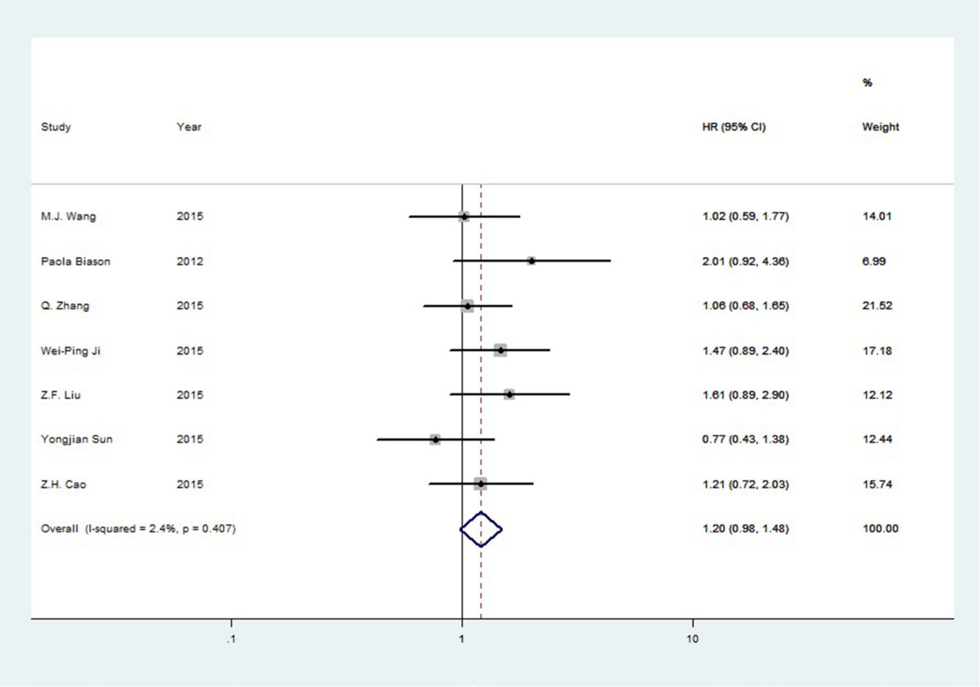

Supplementary Figure 5: Forest plot for OS of rs1799793 G vs. A.

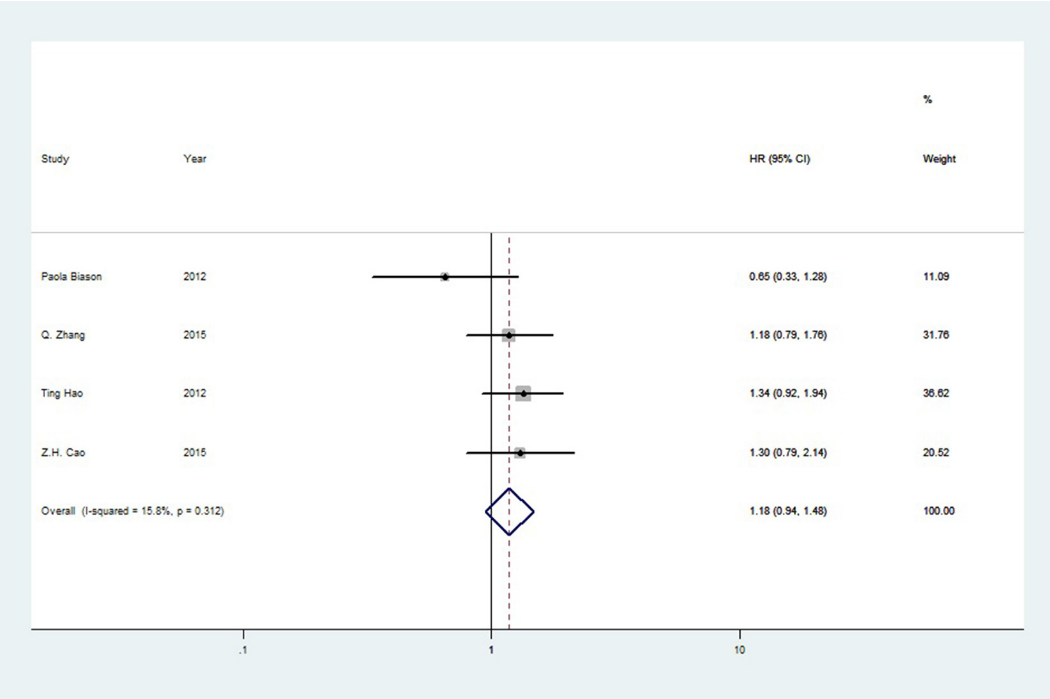

Supplementary Figure 6: Forest plot for OS of rs3212986 C vs. A.

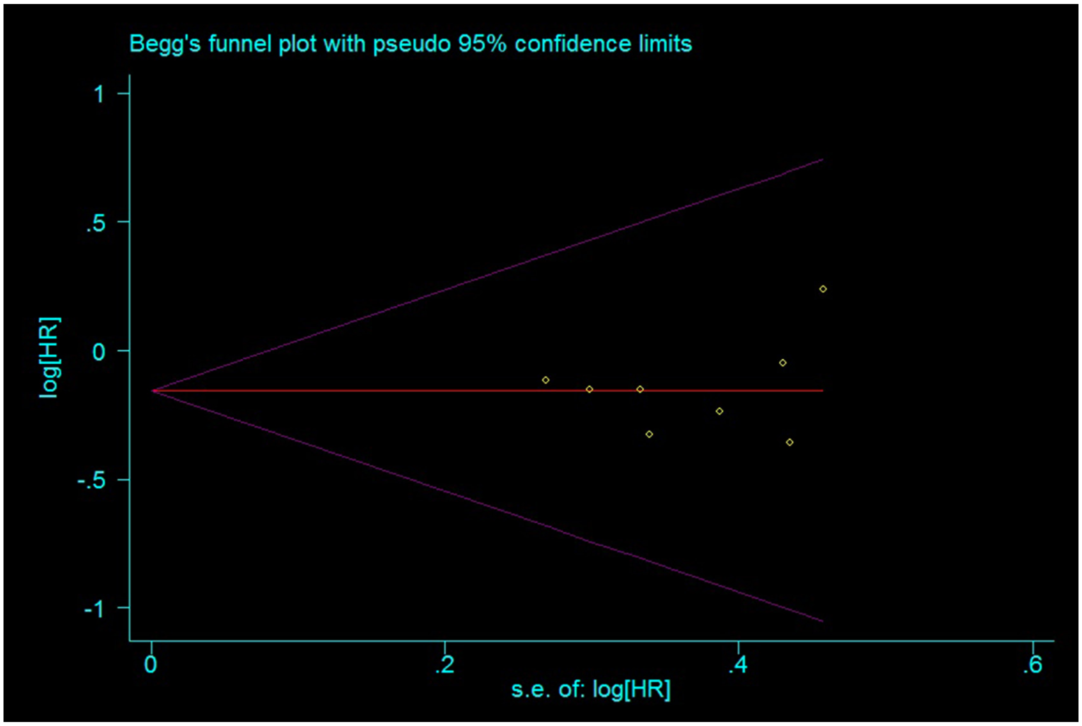

Supplementary Figure 7: Funnel plot for Publication bias, rs13181, OS, AC+CC vs. AA.

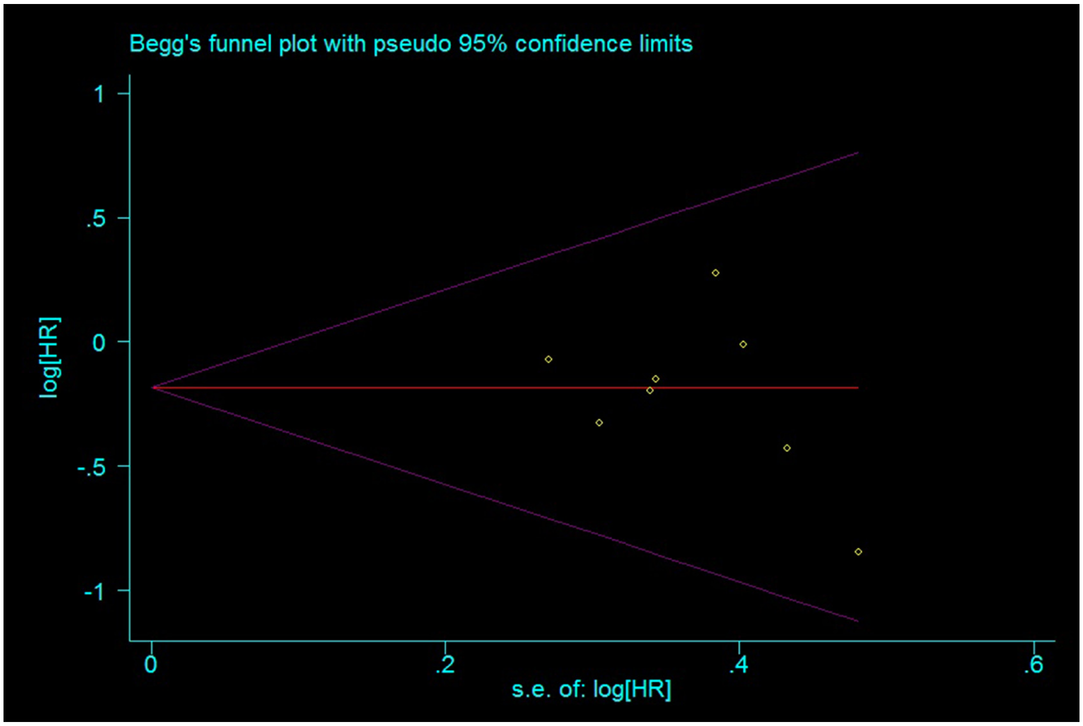

Supplementary Figure 8: Funnel plot for Publication bias, rs1799793, OS, GA+AA vs. GG.

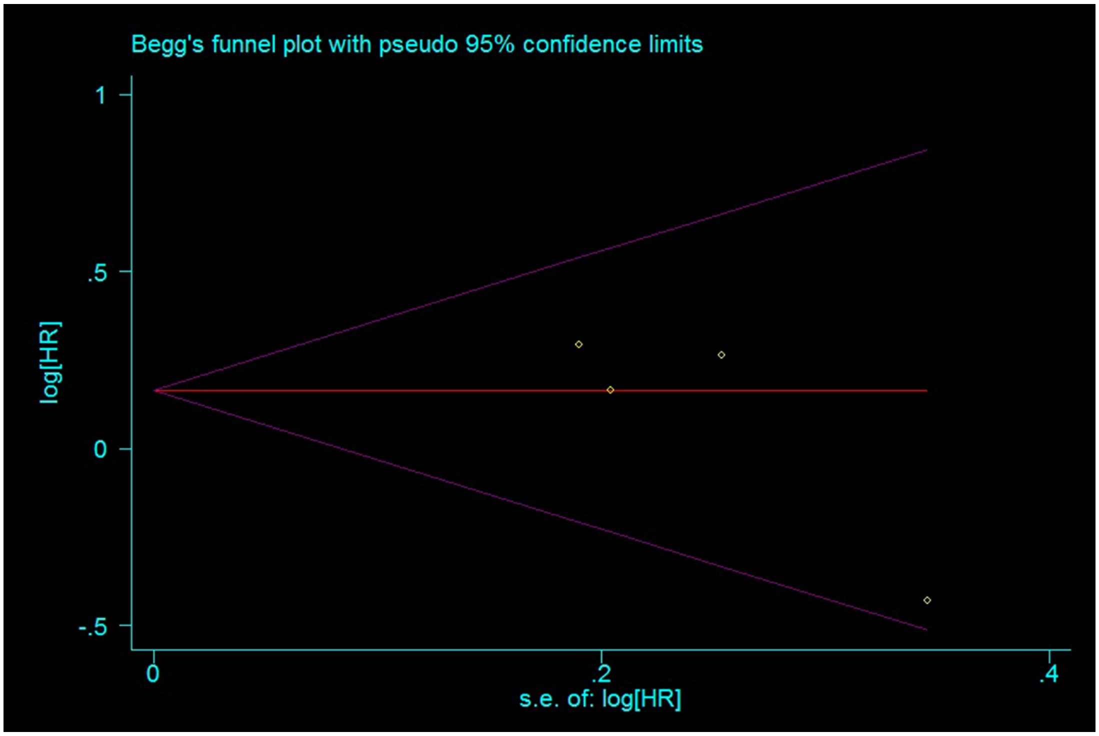

Supplementary Figure 9: Funnel plot for Publication bias, rs3212986, OS, CA+AA vs. CC.

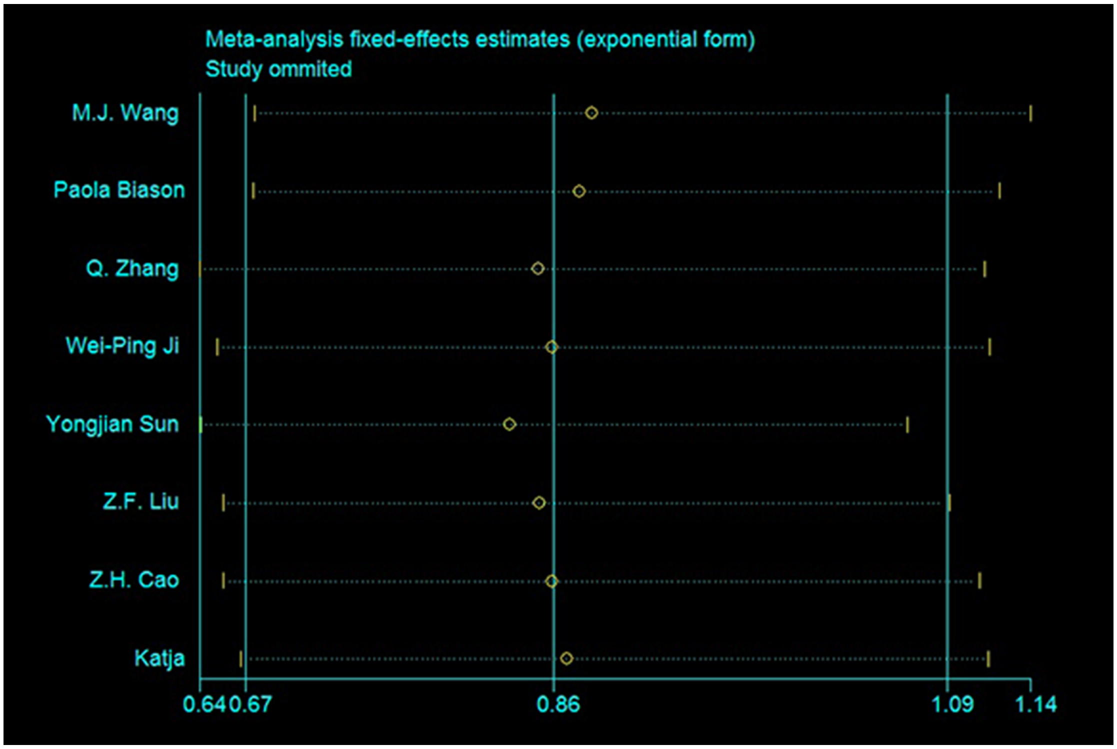

Supplementary Figure 10: Sensitivity analysis of rs13181, OS, AC+CC vs. AA.

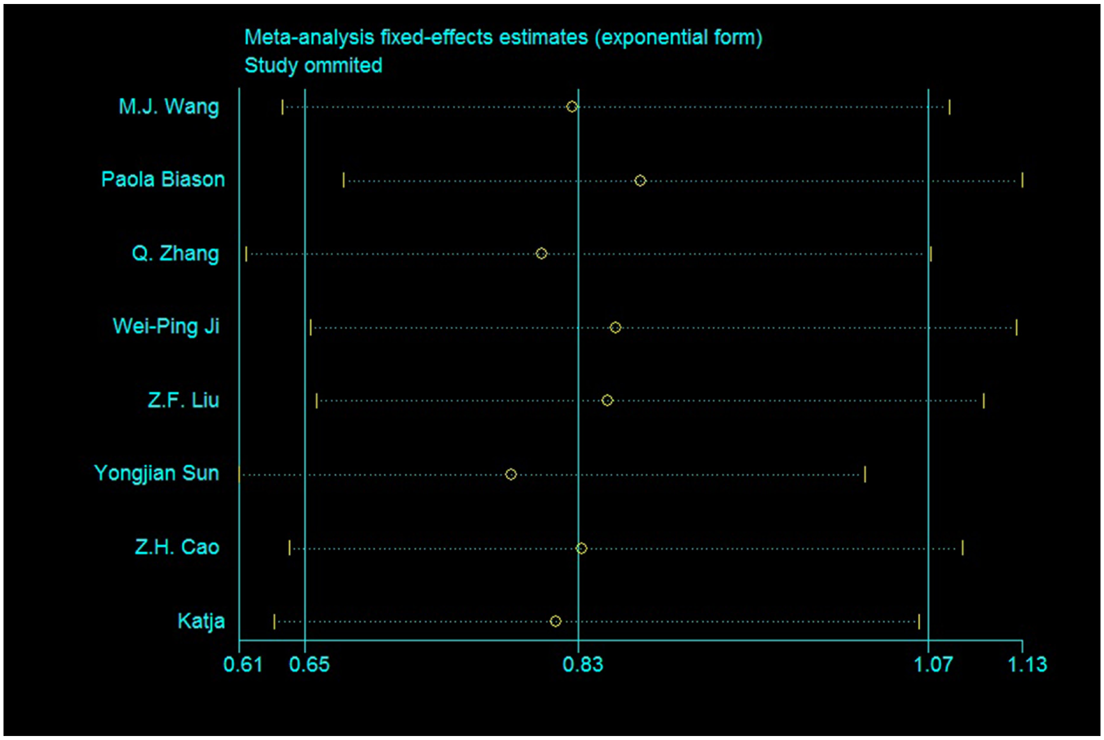

Supplementary Figure 11: Sensitivity analysis of rs1799793, OS, GA+AA vs. GG.

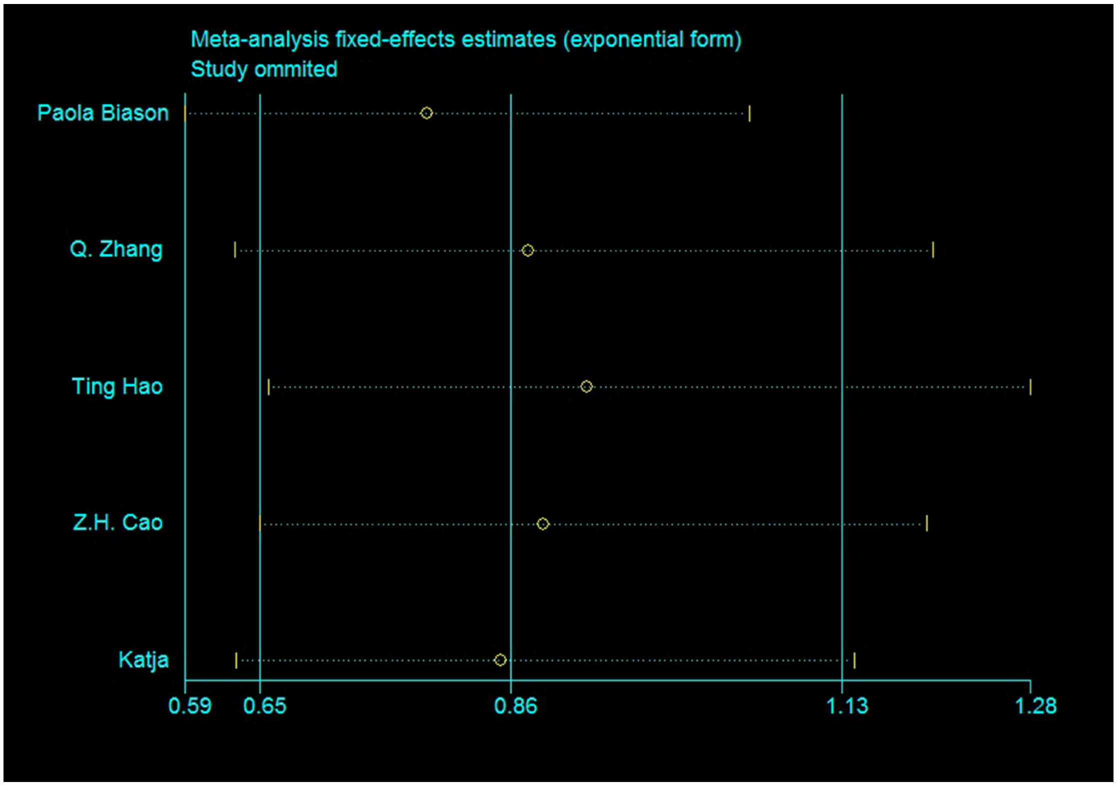

Supplementary Figure 12: Sensitivity analysis of rs3212986, OS, CA+AA vs. AA.

Supplementary Table 1: Characteristics of the included studies for meta-analysis. See Supplementary_Table_1

Supplementary Table 2: Table of data extraction. See Supplementary_Table_2

Supplementary Table 3: Results of meta-analysis. See Supplementary_Table_3

Supplementary Table 4: Results of rs11615 subgroup analysis. See Supplementary_Table_4

Supplementary Table 5: Subgroup analysis: Assessment method of TR. See Supplementary_Table_5

Supplementary Table 6: Subgroup analysis: Confounder adjustment of histology. See Supplementary_Table_6

Supplementary Table 7: Subgroup analysis: Confounder adjustment of metastasis. See Supplementary_Table_7

Supplementary Table 8: Subgroup analysis: Confounder adjustment of tumor location. See Supplementary_Table_8

Supplementary Table 9: Subgroup analysis: Confounder adjustment of tumor stage. See Supplementary_Table_9

Supplementary Table 10: Subgroup analysis: HWE. See Supplementary_Table_10

Supplementary Table 11: Subgroup analysis: Metastasis at diagnosis. See Supplementary_Table_11

Supplementary Table 12: Subgroup analysis: Race. See Supplementary_Table_12

Supplementary Table 13: Subgroup analysis: Treatment. See Supplementary_Table_13

Supplementary Table 14: Literature Strategy. See Supplementary_Table_14

Supplementary Table 15: PRISMA 2009 checklist. See Supplementary_Table_15

Supplementary Table 16: Websites of dbSNP. See Supplementary_Table_16
