# Supplementary material for: Meta-analysis showing that ERCC1 polymorphism is predictive of osteosarcoma prognosis
Source: Oncotarget. 2017 Jul 19;8(37):62769–79. doi: 10.18632/oncotarget.19370 (PMC5617547; doi:10.18632/oncotarget.19370)
Supplement: Supplementary file 2 [file oncotarget-08-62769-s002.doc]

Supplementary Table 1: Characteristics of the included studies for meta-analysis

| First Author | Year | Ethnicity | Number of cases | Ages | Gender  (F/M) | Studying genes and SNPs | Genotyping method | Adjusted confounding factors | Tumor location | Metastasis | Assessment method | Treatment | Stage of tumor | M-FU  (month) |
| --- | --- | --- | --- | --- | --- | --- | --- | --- | --- | --- | --- | --- | --- | --- |
| D Caronia | 2009 | Caucasian | 91 | Mean14.9,range(3.7-34) | 40/51 | ERCC2:rs13181, rs1799793;  ERCC1:rs11615, rs3212986; | TaqMan | Cisplatin doses, tumor  location, metastasis at diagnosis and tumor necrosis | Femur 46, Tibia 32,Arm 7,Central 6 | No 57  At diagnosis 15  At follow-up 19 | Tumor response, EFS | Cisplatin-based chemotherapy | NA | 91(10-272) |
| Katja Goricˇar | 2015 | Caucasian | 66 | Mean17.5,range (13.8–33.3) | 31/35 | ERCC1:rs11615, s3212986;  ERCC2:rs1799793, rs13181 | Kaspar assay | Multi-clinical factors | Extremities 57, Other 9 | NA | Tumor response, EFS, OS | Cisplatin-based chemotherapy; Surgery: Wide resection 39; Amputation 23;  No 3 | I 18  II 36  III10 | 143.0 (109.1–205.6) |
| M.J. Wang | 2015 | Asian | 146 | <20 98;  ≥20 48 | 52/94 | ERCC1:rs11615  ERCC2:rs13181,rs1799793 | PCR-RFLP | Age, gender, tumor stage, type of therapy, tumor location, and metastasis | Long tubular bones 102,  Axial skeleton 44 | Preoperatively:0  Yes:46;No:100 | OS | No preoperative chemotherapy. Amputation 39, Limb salvage 107 | I-II 61  III-IV 85 | NA, patients from 2008-2013 |
| Paola Biason | 2012 | Caucasian | 130 | Median16,range (4-68) | 51/79 | ERCC1:rs11615, rs3212986;  ERCC2: rs13181, rs1799793 | PCR | NA | Extremities 124  Other 6 | At diagnosis:0 | OS,EFS, Relapse ratio | Cisplatin-based chemotherapy | I-II 130  III-IV 0 | 38(1-278) |
| Q. Zhang | 2015 | Asian | 260 | <20 152;  ≥20 108 | 116/144 | ERCC2:rs13181, rs1799793;  ERCC1:rs11615,rs3212986; | PCR-RFLP | Age, gender, grade, family history of cancer, histological type, and tumor location | Extremities 186,  Other 74 | NA | Tumor response, OS | Cisplatin-based chemotherapy | I-II 188  III 72 | NA, patients from 2010-2011 |
| Ting Hao | 2012 | Asian | 267 | Mean age 13.6±5.2  Range 4.5-36 | 92/175 | ERCC1:rs11615,rs3212986；  ERCC2:rs1799793,rs13181 | PCR | Response to treatment and metastasis | Proximal 121,tumor Midshaft 2,Distal 144 | No 134  At diagnosis 57  At follow up 76 | OS,EFS | Cisplatin-based chemotherapy | NA | 44.3 |
| Wei-Ping Ji | 2015 | Asian | 214 | ≤20 123, >20 91 | 81/133 | ERCC1 rs11615  ERCC2 rs1799793,rs13181 | PCR-RFLP | Age, tumor size, clinical stage, lymph node metastasis and ER and PR status | Long tubular bones 158,Axial skeleton 56 | At diagnosis:0  Yes:54  No:151 | Tumor response, OS | Cisplatin-based chemotherapy Amputation 51,limb salvage 163 | I-II 141  III-IV 73 | 36.5(4-60) |
| Li-Min Yang | 2012 | Asian | 187 | <10 10,  10~14 48  15-30 77  >30 52 | 81/106 | ERCC1:rs11615  ERCC2: rs13181 | PCR-RFLP | Sex, age, subtype, location, metastasis, tumor size and anatomic location | Tibia/femur 119,  Elsewhere 68 | No 97  At diagnosis 38  At follow-up 52 | Tumor response, OS | Cisplatin-based chemotherapy before and after surgery | IIA 77  IIB-III 110 | NA, patients from Jan.2005-Jan.2007 |
| Yongjian Sun | 2015 | Asian | 172 | <20 107,  ≥20 65 | 58/114 | ERCC1 rs11615,rs2298881  ERCC2 rs1799793,rs13181 | PCR-RFLP | Age, gender, tumor stage and histological type, tumor location and metastasis | Long tubular bones 107,Axial skeleton 65 | At diagnosis:0  Yes:61  No:111 | Tumor response, OS | No radiotherapy or chemotherapy before enrolled.  Amputation 57,limb salvage 115 | I-II 97  III 75 | NA, patients from 2009-2011 |
| Z.F. Liu | 2015 | Asian | 115 | <20 71,  ≥20 44 | 50/65 | ERCC2 rs1799793,rs13181 | PCR | Gender, age, tumor stage, location, and histological type | Extremities 81,Other 34 | NA | Tumor response, OS | Cisplatin-based chemotherapy | I-II 90  III-IV 25 | NA |
| Z.H. Cao | 2015 | Asian | 186 | <20 98,  ≥20 88 | 79/107 | ERCC1 rs11615,rs2298881, rs3212986;  ERCC2 rs1799793,rs13181 | PCR-RFLP | Gender, age, stage, tumor location, and therapy | Long tubular bones 122,Axial skeleton 64 | NA | Tumor response, OS | Cisplatin-based chemotherapy, Amputation 47,limb salvage 139 | I-II 127  III-IV 59 | 38.5(3-60) |
