# Supplementary material for: Meta-analysis showing that ERCC1 polymorphism is predictive of osteosarcoma prognosis
Source: Oncotarget. 2017 Jul 19;8(37):62769–79. doi: 10.18632/oncotarget.19370 (PMC5617547; doi:10.18632/oncotarget.19370)
Supplement: Supplementary file 3 [file oncotarget-08-62769-s003.doc]

Supplementary Table 2: Table of data extraction

| First author | Index | Locus | Gene distribution | Primary OR(HR) value and 95%CI | HWE |
| --- | --- | --- | --- | --- | --- |
| D Caronia | PTR | rs13181 | TT/TG/GG: 39/40/12 | TG/TT=5.54 (1.76-17.39), GG/TT=3.20 (0.65-15.70), TG/GG=4.89 (1.64-14.54) | 0.731 |
|  |  | rs1799793 | GG/AG/AA:39/42/10 | AG/GG=1.52 (0.54-4.28), AA/GG=3.50 (0.69-17.64), AG/AA=1.80 (0.67-4.80) | 0.794 |
|  |  | rs3212986 | GG/GT/TT:50/30/11 | GT/GG=1.64 (0.56-4.78),TT/GG=2.51 (0.63-10.05),GT/TT=1.87 (0.71-4.94) | 0.067 |
|  |  | rs11615 | TT/TC/CC:29/42/20 | CT/TT=1.37(0.43-4.32),CC/TT=1.78(0.48-6.62),TC/CC=1.50(0.51-4.37) | 0.528 |
|  | EFS | rs13181 |  | TG/TT=5.06 (1.09-23.46), GG/TT=8.33 (1.52-45.56), TG/GG=5.76 (1.30-25.55) |  |
|  |  | rs1799793 |  | AG/GG=1.71 (0.50-5.87), AA/GG=3.83 (0.95-15.47), AG/AA=2.14 (0.68-6.74) |  |
|  |  | rs3212986 |  | GT/GG=2.43 (0.76-7.77),TT/GG=2.05 (0.49-8.65),GT/TT=2.30 (0.78-6.75) |  |
|  |  | rs11615 |  | CT/TT=2.80(0.59-13.20),CC/TT=3.35 (0.64-17.48),TC/CC=2.98(0.67-13.27) |  |
| Katja Goričar | GTR | rs13181 | AA/AC+CC:6/18 | AC+CC/AA=6.96 (1.33-36.43) |  |
|  |  | rs1799793 | GG/GA+AA:9/15 | GA+AA/GG=0.76 (0.19-2.99) |  |
|  |  | rs3212986 | GG/GT+TT:10/14 | GT+TT/GG=2.48 (0.63-9.79) |  |
|  |  | rs11615 | TT/TC+CC:9/15 | TC+CC/TT=0.77 (0.20-3.05) |  |
|  | EFS | rs13181 |  | AC+CC/AA=1.10 (0.40-3.07) |  |
|  |  | rs1799793 |  | GA+AA/GG=0.28 (0.11-0.70) |  |
|  |  | rs3212986 |  | GT+TT/GG=2.09 (0.86-5.08) |  |
|  |  | rs11615 |  | TC+CC/TT=0.96 (0.43-2.14) |  |
|  | OS | rs13181 |  | AC+CC/AA=0.79 (0.37-1.69) |  |
|  |  | rs1799793 |  | GA+AA/GG=0.99 (0.45-2.18) |  |
|  |  | rs3212986 |  | GT+TT/GG=0.92 (0.42-1.98) |  |
|  |  | rs11615 |  | TC+CC/TT=1.05 (0.50-2.19) |  |
| M.J. Wang | OS  Patients(death) | rs13181 | AA/AC/CC:78(36)/57(22)/11(4) | AC/AA= 0.72 (0.31-1.42), CC/AA= 0.53 (0.11-2.16) | 0.895 |
|  |  | rs1799793 | GG/GA/AA:91(43)/44(18)/11(6) | GA/GG= 0.97 (0.41-2.14), AA/GG= 0.22 (0.12-0.93) | 0.094 |
|  |  | rs11615 | CC/CT/TT:60(30)/68(27)/18(5) | CT/CC=0.57 (0.27-1.21), TT/CC= 0.24 (0.08-0.96) | 0.851 |
| Paola Biason | EFS  Percentage of probability to event free  at 5 years | rs13181 | AA/AC/CC:42%/53%/82% | AC/AA= 0.96 (0.54-1.69); CC/AA=0.57 (0.19-1.73) | 0.583 |
|  |  | rs1799793 | GG/GA/AA:40%/66%/80% | GA/GG=0.53 (0.27-1.04); AA/GG= 0.23 (0.05-0.99) | 0.012 |
|  |  | rs3212986 | CC/CA/AA:53%/54%/25% | CA/CC=0.86 (0.48-1.55);AA/CC=1.78 (0.69-4.58) | 0.716 |
|  |  | rs11615 | TT/CT/CC:48%/63%/37% | TC/TT=0.67 (0.36-1.26);CC/TT=1.18 (0.61-2.29) | 0.496 |
|  | OS  N. Death/N. Total (%) | rs13181 | AA/AC/CC:49(12)/64(13)/17(2);67%/72%/88% | AC/AA= 0.73 (0.33-1.61); CC/AA=0.41 (0.09-1.83) | 0.583 |
|  |  | rs1799793 | GG/GA/AA: 77(20)/38(5)/15(2);67%/85%/69% | GA/GG=0.50 (0.19-1.34); AA/GG= 0.48 (0.11-2.05) | 0.012 |
|  |  | rs3212986 | CC/CA/AA:72(12)/44(12)/8(2);75%/67%/69% | CA/CC= 0.86 (0.48-1.55); AA/CC= 1.78 (0.69-4.58) | 0.716 |
|  |  | rs11615 | TT/CT/CC:37(8)/59(9)/30(9);76%/80%/62% | TC/TT= 0.71 (0.28-1.85); CC/TT=1.31 (0.51-3.40) | 0.496 |
| Q. Zhang | TR  Cases(responders) | rs11615 | TT/CT/CC:113(53)/109(68)/38(31) | TC/TT= 1.61 (0.91-2.86); CC/TT=2.87 (1.24-6.97) | 0.168 |
|  |  | rs3212986 | GG/GT/TT: 118(63)/120(72)/22(17) | TG/GG=1.21 (0.70-2.10); TT/GG=2.01 (0.72-6.14) | 0.267 |
|  |  | rs1799793 | GG/GA/AA: 158(87)/86(53)/16(12) | GA/GG=1.22 (0.69-2.16);AA/GG=1.56 (0.51-5.33) | 0.356 |
|  |  | rs13181 | AA/AC/CC: 150(82)/94(59)/16(11) | AC/AA=1.26 (0.72-2.21);CC/AA=1.45 (0.46-5.04) | 0.804 |
|  | OS  Cases(events) | rs11615 | TT/CT/CC:113(44)/109(33)/38(7) | TC/TT= 0.68 (0.38-1.23); CC/TT=0.35 (0.12-0.92) | 0.168 |
|  |  | rs3212986 | GG/GT/TT: 118(41)/120(37)/22(6) | TG/GG=0.84 (0.47-1.49); TT/GG=0.70 (0.21-2.08) | 0.267 |
|  |  | rs1799793 | GG/GA/AA: 158(52)/86(27)/16(5) | GA/GG=0.93 (0.51-1.70);AA/GG=0.93 (0.24-3.08) | 0.356 |
|  |  | rs13181 | AA/AC/CC: 150(50)/94(30)/16(4) | AC/AA=0.94 (0.51-1.68);CC/AA=0.67 (0.15-2.35) | 0.804 |
| Ting Hao | OS | rs11615 | CC/CT/TT:118/113/36  5-year survival:49%/58.2%/68% | CT/CC=0.82(0.50-1.33); TT/CC=0.57(0.23-1.23) | 0.285 |
|  |  | rs3212986 | CC/CA/AA:132/104/32  5-year survival:51.7%/57%/65.6% | CA/CC= 0.89(0.53-1.43); AA/CC= 0.71(0.30-1.55) | 0.107 |
|  |  | rs1799793 | GG/GT/TT:124/108/34  5-year survival:52%/58%/59.9% | GT/GG= 0.86(0.52-1.41); TT/GG= 0.84(0.37-1.76) | 0.176 |
|  |  | rs13181 | GG/GA/AA:157/92/18  5-year survival:51%/59.1%/74.8% | GA/GG= 0.83(0.50-1.36); AA/GG= 0.55(0.14-1.64) | 0.372 |
|  | EFS | rs11615 |  | CT/CC=0.67(0.32-1.24); TT/CC=0.39(0.14-0.95) |  |
|  |  | rs3212986 |  | CA/CC= 0.77(0.32-1.53); AA/CC= 0.65(0.43-1.76) |  |
|  |  | rs1799793 |  | GT/GG= 0.83(0.41-1.56); TT/GG= 0.82(0.30-1.85) |  |
|  |  | rs13181 |  | GA/GG= 0.73(0.35-1.73); AA/GG= 0.44(0.10-0.87) |  |
| Wei-Ping Ji | TR  (GTR,PTR) | rs11615 | TT/CT/CC:52,41/54,31/27,9 | CT/TT=1.37 (0.72-2.62); CC/TT=2.51 (1.02-6.85) | 0.034 |
|  |  | rs1799793 | GG/GA/AA: 74,54/39,20/21,7 | GA/GG=1.42 (0.72-2.87); AA/GG= 2.19 (0.82-6.52) | <0.01 |
|  |  | rs13181 | AA/AC/CC: 76,49/41,24/15,8 | AC/AA=1.10 (0.57-2.15);CC/AA=1.21 (0.44-3.55) | 0.002 |
|  | OS  (death, alive) | rs11615 | TT/CT/CC:34,61/24,58/8,29 | CT/TT=0.74 (0.37-1.47); CC/TT=0.43 (0.15-0.93) | 0.011 |
|  |  | rs1799793 | GG/GA/AA: 74,54/39,20/21,7 | GA/GG=0.83 (0.41-1.67); AA/GG= 0.44 (0.10-1.46) | <0.01 |
|  |  | rs13181 | AA/AC/CC: 76,49/41,24/15,8 | AC/AA=0.88 (0.43-1.75);CC/AA=0.82 (0.27-2.27) | 0.002 |
| Li-min Yang | TR  (Responders, non-responders: CR+PR,SD+PD) | rs11615 | TT/CT/CC:47,46/38,35/12,9 | CT/TT=1.34(0.64-2.45);CC/TT=1.65(0.65-4.13) | 0.254 |
|  |  | rs13181 | AA/AC/CC: 54,55/35,32/9,4 | AC/AA=1.34(0.75-2.78);CC/AA=2.88(1.14-13.25) | 0.541 |
|  | OS  (cases, deaths) | rs11615 | CC/CT/TT:93,42/73,30/21,7 | CT/CC=0.81(0.40-1.33); TT/CC=0.74 (0.28-1.94) | 0.254 |
|  |  | rs13181 | GG/GA/AA: 107,50/67,26/13,3 | AC/AA=0.73(0.40-1.12);CC/AA=0.32 (0.13-0.95) | 0.574 |
| Yongjian Sun | TR  (good-responders, non-responders) | rs11615 | CC/CT/TT:81,35/16,15/10,15 | TC/CC=0.46 (0.19-1.13); TT/CC=0.27 (0.10-0.71) | <0.01 |
|  |  | rs1799793 | GG/GA/AA: 79,42/17,13/11,10 | GA/GG=0.70 (0.29-1.72);AA/GG=0.58 (0.21-1.68) | <0.01 |
|  |  | rs13181 | AA/AC/CC: 92,51/10,8/5,6 | AC/AA=0.69 (0.23-2.16);CC/AA=0.46 (0.11-1.93) | <0.01 |
|  | OS  (cases, deaths) | rs11615 | CC/CT/TT:116(22)/31(8)/25(11) | TC/CC=1.49 (0.50-4.03); TT/CC=3.36 (1.19-9.16) | <0.01 |
|  |  | rs1799793 | GG/GA/AA:121(27)/30(8)/21(6) | GA/GG=1.27 (0.44-3.38);AA/GG=1.39 (0.40-4.26) | <0.01 |
|  |  | rs13181 | AA/AC/CC: 143(33)/18(5)/11(3) | AC/AA=1.28 (0.33-4.19);CC/AA=1.25 (0.20-5.60) | <0.01 |
| Z.F. Liu | TR  (CR+PR,SD+PD) | rs1799793 | GG/GA/AA: 32,22/34,13/12,2 | GA/GG=1.96 (0.78-4.86);AA/GG=4.85 (1.06-42.71) | 0.452 |
|  |  | rs13181 | AA/AC/CC: 29,17/36,16/13,4 | AC/AA=1.47 (0.61-3.77);CC/AA=2.16 (0.52-10.72) | 0.714 |
|  | OS  (cases, deaths) | rs1799793 | GG/GA/AA:54(22)/47(18)/14(2) | GA/GG=0.87 (0.34-1.94);AA/GG=0.23 (0.021-0.97) | 0.452 |
|  |  | rs13181 | AA/AC/CC: 46(17)/52(19)/17(6) | AC/AA=0.95 (0.36-2.18);CC/AA=0.88 (0.25-3.57) | 0.714 |
| Z.H. Cao | TR  (GTR, PTR) | rs11615 | TT/CT/CC:43,38/46,28/23,8 | CT/TT=1.45 (0.73-2.90); CC/TT=2.56 (1.02-7.35) | 0.052 |
|  |  | rs3212986 | CC/CA/AA:54,40/45,29/13,5 | CA/CC= 1.15 (0.59-2.24); AA/CC= 1.93 (0.59-7.45) | 0.541 |
|  |  | rs1799793 | GG/GA/AA:65,48/32,19/16,7 | GA/GG= 1.24 (0.60-2.62);AA/GG= 1.69 (0.60-5.23) | <0.01 |
|  |  | rs13181 | AA/AC/CC:63,46/34,21/15,7 | AC/AA= 1.18 (0.58-2.43); CC/AA= 1.56 (0.54-4.90) | 0.001 |
|  | OS  (death, survivor) | rs11615 | TT/CT/CC:29,53/20,55/4,23 | CT/TT=0.66 (0.32-1.39); CC/TT=0.32 (0.07-0.98) | 0.155 |
|  |  | rs3212986 | CC/CA/AA:28,60/21,56/4,15 | CA/CC= 0.85 (0.41-1.76); AA/CC= 0.60 (0.13-2.14) | 0.723 |
|  |  | rs1799793 | GG/GA/AA:34,78/12,32/7,21 | GA/GG=0.89 (0.37-2.04);AA/GG=0.79 (0.26-2.17) | <0.01 |
|  |  | rs13181 | AA/AC/CC:33,77/16,42/4,12 | AC/AA= 0.89 (0.41-1.89); CC/AA= 0.78 (0.17-2.82) | 0.046 |
