# Supplementary material for: Meta-analysis showing that ERCC1 polymorphism is predictive of osteosarcoma prognosis
Source: Oncotarget. 2017 Jul 19;8(37):62769–79. doi: 10.18632/oncotarget.19370 (PMC5617547; doi:10.18632/oncotarget.19370)
Supplement: Supplementary file 4 [file oncotarget-08-62769-s004.doc]

Supplementary Table 3: Results of meta-analysis

| Index | Locus | Genetic models | Number of studies | Test of association | | Test of heterogeneity | | | | Test of association after sensitivity analysis | | | | Test of heterogeneity after sensitivity analysis | | | | Test of publication bias | |
| --- | --- | --- | --- | --- | --- | --- | --- | --- | --- | --- | --- | --- | --- | --- | --- | --- | --- | --- | --- |
|  |  |  |  | HR/OR (95%CI) | P-value | Model | Chi-square | P-value | I² | OR/HR (95%CI) | P-value | Study removed as heterogeneity source | Percentage of removed studies (%) | Model | Chi-square | P-value | I² | Begg's test(P) | Egger's test(P) |
| OS | rs13181 | AC vs. AA | 7 | 0.869 (0.652-1.158) | 0.336 | F | 0.90 | 0.989 | 0.00% |  |  |  |  |  |  |  |  | 0.368 | 0.300 |
|  |  | CC vs. AA |  | 0.746 (0.449-1.241) | 0.259 | F | 1.12 | 0.981 | 0.00% |  |  |  |  |  |  |  |  | 1.000 | 0.948 |
|  |  | AC vs. CC |  | 1.187 (0.735-1.916) | 0.484 | F | 0.54 | 0.994 | 0.00% |  |  |  |  |  |  |  |  | 0.368 | 0.349 |
|  |  | AC+CC vs. AA | 8 | 0.856 (0.671-1.092) | 0.210 | F | 1.34 | 0.987 | 0.00% |  |  |  |  |  |  |  |  | 1.000 | 0.670 |
|  |  | A vs. C | 7 | 1.169 (0.956-1.431) | 0.129 | F | 1.99 | 0.921 | 0.00% |  |  |  |  |  |  |  |  | 1.000 | 0.543 |
|  | rs11615 | TC vs. TT | 7 | 0.831 (0.624-1.108) | 0.208 | F | 6.57 | 0.476 | 0.00% |  |  |  |  |  |  |  |  | 0.536 | 0.430 |
|  |  | CC vs. TT |  | 0.814 (0.431-1.536) | 0.525 | R | 23.34 | 0.001 | 70.00% |  |  |  |  |  |  |  |  | 1.000 | 0.935 |
|  |  | TC vs. CC |  | 0.915 (0.698-1.201) | 0.523 | F | 10.74 | 0.150 | 34.80% |  |  |  |  |  |  |  |  | 0.536 | 0.157 |
|  |  | TC+CC vs. TT | 8 | 0.845 (0.592-1.206) | 0.353 | R | 16.15 | 0.040 | 50.50% |  |  |  |  |  |  |  |  | 0.118 | 0.169 |
|  |  | T vs. C | 7 | 1.133 (0.800-1.605) | 0.481 | F | 31.41 | <0.001 | 77.70% |  |  |  |  |  |  |  |  | 0.711 | 0.710 |
|  | rs1799793 | GA vs. GG | 7 | 0.875 (0.647-1.184) | 0.386 | F | 1.90 | 0.929 | 0.00% | - |  |  |  |  |  |  |  | 1.000 | 0.778 |
|  |  | AA vs. GG |  | 0.551 (0.343-0.884) | 0.014 | F | 7.47 | 0.280 | 19.70% | 0.462 (0.276-0.774) | 0.003 | Yongjian Sun et al. | 16.00 | F | 4.67 | 0.457 | 0.00% | 0.764 | 0.793 |
|  |  | GA vs. AA |  | 1.179 (0.724-1.919) | 0.509 | F | 3.93 | 0.686 | 0.00% | - |  |  |  |  |  |  |  | 1.000 | 0.613 |
|  |  | GA+AA vs. GG | 8 | 0.834 (0.653-1.065) | 0.146 | F | 4.25 | 0.751 | 0.00% | - |  |  |  |  |  |  |  | 0.386 | 0.376 |
|  |  | G vs. A | 7 | 1.204 (0.980-1.479) | 0.077 | F | 6.15 | 0.407 | 2.40% | 1.283 (1.030-1.598) | 0.026 | Yongjian Sun et al. | 12.44 | F | 3.57 | 0.613 | 0.00% | 0.548 | 0.364 |
|  | rs3212986 | CA vs. CC | 4 | 0.863 (0.646-1.154) | 0.321 | F | 0.03 | 0.999 | 0.00% | - |  |  |  |  |  |  |  | 0.734 | 0.320 |
|  |  | AA vs. CC |  | 0.903 (0.543-1.501) | 0.693 | F | 2.82 | 0.420 | 0.00% | - |  |  |  |  |  |  |  | 1.000 | 0.732 |
|  |  | CA vs. AA |  | 1.336 (0.780-2.287) | 0.291 | F | 0.14 | 0.987 | 0.00% | - |  |  |  |  |  |  |  | 0.734 | 0.387 |
|  |  | CA+AA vs. CC | 5 | 0.857 (0.652-1.127) | 0.271 | F | 3.53 | 0.473 | 0.00% | - |  |  |  |  |  |  |  | 0.086 | 0.106 |
|  |  | C vs. A | 4 | 1.180 (0.942-1.479) | 0.150 | F | 3.56 | 0.312 | 15.80% | 1.272 (1.001-1.616) | 0.049 | Paola Biason et al. | 11.09 | F | 0.22 | 0.987 | 0.00% | 0.308 | 0.148 |
| Good tumor response | rs13181 | AC vs. AA | 5 | 1.166 (0.841-1.615) | 0.356 | F | 1.20 | 0.879 | 0.00% | - |  |  |  |  |  |  |  | 0.806 | 0.375 |
|  |  | CC vs. AA |  | 1.253 (0.726-2.161) | 0.418 | F | 2.59 | 0.628 | 0.00% | - |  |  |  |  |  |  |  | 0.806 | 0.746 |
|  |  | AC vs. CC |  | 0.852 (0.509-1.426) | 0.542 | F | 0.74 | 0.946 | 0.00% | - |  |  |  |  |  |  |  | 1.000 | 0.336 |
|  |  | AC+CC vs. AA | 6 | 1.268 (0.950-1.693) | 0.107 | F | 8.26 | 0.142 | 39.50% | 1.202 (0.897-1.612) | 0.218 | Katja et al. | 3.04 | F | 4.07 | 0.397 | 1.70% | 0.707 | 0.412 |
|  |  | A vs. C | 5 | 0.851 (0.682-1.062) | 0.154 | F | 5.73 | 0.220 | 30.20% | 0.777 (0.614-0.983) | 0.035 | Sun Yongjian et al. | 11.17 | F | 0.53 | 0.913 | 0.00% | 0.462 | 0.180 |
|  | rs11615 | TC vs. TT | 5 | 1.398 (1.010-1.935) | 0.043 | F | 1.52 | 0.823 | 0.00% |  |  |  |  |  |  |  |  | 0.806 | 0.361 |
|  |  | CC vs. TT |  | 2.237 (1.477-3.388) | <0.001 | F | 6.09 | 0.193 | 34.30% |  |  |  |  |  |  |  |  | 0.462 | 0.402 |
|  |  | TC vs. CC |  | 0.633 (0.444-0.903) | 0.012 | F | 4.79 | 0.309 | 16.50% |  |  |  |  |  |  |  |  | 0.462 | 0.054 |
|  |  | TC+CC vs. TT | 6 | 1.755 (1.330-2.316) | <0.001 | F | 6.90 | 0.228 | 27.50% |  |  |  |  |  |  |  |  | 0.260 | 0.247 |
|  |  | T vs. C | 5 | 0.600 (0.424-0.850) | 0.004 | R | 12.75 | 0.013 | 68.60% |  |  |  |  |  |  |  |  | 1.000 | 0.927 |
|  | rs1799793 | GA vs. GG | 5 | 1.248 (0.901-1.727) | 0.182 | F | 2.70 | 0.610 | 0.00% |  |  |  |  |  |  |  |  | 0.806 | 0.935 |
|  |  | AA vs. GG |  | 1.479 (0.881-2.482) | 0.139 | F | 5.32 | 0.256 | 24.80% | 2.014 (1.108-3.660) | 0.022 | Sun Yongjian et al. | 24.81 | F | 1.18 | 0.759 | 0.00% | 0.806 | 0.286 |
|  |  | GA vs. AA |  | 0.708 (0.423-1.185) | 0.189 | F | 1.40 | 0.845 | 0.00% |  |  |  |  |  |  |  |  | 0.462 | 0.395 |
|  |  | GA+AA vs. GG | 6 | 1.308 (0.998-1.715) | 0.052 | F | 7.08 | 0.214 | 29.40% | 1.500 (1.115-2.017) | 0.007 | Sun Yongjian et al. | 16.34 | F | 2.08 | 0.722 | 0.00% | 0.707 | 0.708 |
|  |  | G vs. A | 5 | 0.752(0.533-1.061) | 0.104 | R | 9.59 | 0.048 | 58.30% | 0.645 (0.506-0.821) | <0.001 | Sun Yongjian et al. | 19.15 | F | 1.04 | 0.793 | 0.00% | 1.000 | 0.958 |
|  | rs3212986 | CA vs. CC | 2 | 1.185 (0.776-1.811) | 0.432 | F | 0.01 | 0.908 | 0.00% |  |  |  |  |  |  |  |  | 1.000 | NA |
|  |  | AA vs. CC |  | 1.976 (0.872-4.480) | 0.103 | F | 0.00 | 0.962 | 0.00% |  |  |  |  |  |  |  |  | 1.000 | NA |
|  |  | CA vs. AA |  | 0.509 (0.233-1.111) | 0.090 | F | 0.15 | 0.697 | 0.00% |  |  |  |  |  |  |  |  | 1.000 | NA |
|  |  | CA+AA vs. CC | 3 | 1.436 (0.998-2.075) | 0.052 | F | 0.81 | 0.668 | 0.00% |  |  |  |  |  |  |  |  | 1.000 | 0.415 |
|  |  | C vs. A | 2 | 0.722 (0.539-0.968) | 0.029 | F | 0.13 | 0.718 | 0.00% |  |  |  |  |  |  |  |  | 1.000 | NA |
| Poor tumor response | rs13181 | AC vs. AA | 6 | 1.066 (0.673-1.688) | 0.785 | F | 11.24 | 0.047 | 55.50% | 0.847 (0.626-1.146) | 0.283 | D Caronia et al. | 6.51 | F | 1.59 | 0.811 | 0.00% | 0.133 | 0.048 |
|  |  | CC vs. AA |  | 0.877 (0.553-1.390) | 0.576 | F | 6.34 | 0.274 | 21.20% | 0.779 (0.481-1.260) | 0.131 | D Caronia et al. | 8.37 | F | 3.57 | 0.467 | 0.00% | 0.707 | 0.200 |
|  |  | AC vs. CC |  | 1.524 (0.956-2.427) | 0.076 | F | 6.10 | 0.296 | 18.10% | 1.175 (0.702-1.967) | 0.540 | D Caronia et al. | 18.22 | F | 0.74 | 0.947 | 0.00% | 1.000 | 0.522 |
|  |  | AC+CC vs. AA |  | 0.818 (0.625-1.072) | 0.146 | F | 4.26 | 0.512 | 0.00% |  |  |  |  |  |  |  |  | 1.000 | 0.863 |
|  |  | A vs. C | 5 | 1.171 (0.938-1.461) | 0.163 | F | 5.56 | 0.235 | 28.00% | 1.280 (1.012-1.619) | 0.039 | Sun Yongjian et al. | 12.93 | F | 0.52 | 0.914 | 0.00% | 0.462 | 0.190 |
|  | rs11615 | TC vs. TT | 6 | 0.707 (0.525-0.952) | 0.022 | F | 3.65 | 0.601 | 0.00% |  |  |  |  |  |  |  |  | 0.260 | 0.084 |
|  |  | CC vs. TT |  | 0.494 (0.263-0.926) | 0.028 | R | 13.08 | 0.023 | 61.80% |  |  |  |  |  |  |  |  | 0.566 | 0.128 |
|  |  | TC vs. CC |  | 1.568 (1.118-2.199) | 0.009 | F | 4.72 | 0.451 | 0.00% |  |  |  |  |  |  |  |  | 1.000 | 0.127 |
|  |  | TC+CC vs. TT |  | 1.239 (0.932-1.649) | 0.141 | F | 2.76 | 0.736 | 0.00% |  |  |  |  |  |  |  |  | 0.452 | 0.168 |
|  |  | T vs. C | 5 | 1.676 (1.214-2.314) | 0.002 | R | 12.80 | 0.025 | 60.90% |  |  |  |  |  |  |  |  | 1.000 | 0.945 |
|  | rs1799793 | GA vs. GG | 6 | 0.833 (0.624-1.111) | 0.214 | F | 4.31 | 0.505 | 0.00% |  |  |  |  |  |  |  |  | 0.707 | 0.289 |
|  |  | AA vs. GG |  | 0.721 (0.364-1.428) | 0.348 | R | 10.76 | 0.056 | 53.50% | 0.554 (0.331-0.926) | 0.024 | D Caronia et al. | 11.45 | F | 6.47 | 0.167 | 38.20% | 1.000 | 0.940 |
|  |  | GA vs. AA |  | 1.494 (0.944-2.364) | 0.086 | F | 1.57 | 0.905 | 0.00% |  |  |  |  |  |  |  |  | 1.000 | 0.582 |
|  |  | GA+AA vs. GG |  | 0.765 (0.587-0.997) | 0.047 | F | 6.80 | 0.236 | 26.40% | 0.671 (0.503-0.895) | 0.007 | Sun Yongjian et al. | 15.69 | F | 1.71 | 0.788 | 0.00% | 0.707 | 0.672 |
|  |  | G vs. A | 5 | 1.330 (0.946-1.869) | 0.101 | R | 9.36 | 0.053 | 57.30% | 1.550 (1.216-1.975) | <0.001 | Sun Yongjian et al. | 19.26 | F | 0.94 | 0.815 | 0.00% | 1.000 | 0.989 |
|  | rs3212986 | CA vs. CC | 3 | 0.874 (0.604-1.266) | 0.476 | F | 1.61 | 0.447 | 0.00% |  |  |  |  |  |  |  |  | 0.296 | 0.015 |
|  |  | AA vs. CC |  | 0.704 (0.235-2.115) | 0.532 | R | 5.28 | 0.071 | 62.10% | 0.415 (0.194-0.889) | 0.024 | D Caronia et al. | 29.04 | F | 0.30 | 0.585 | 0.00% | 0.296 | 0.013 |
|  |  | CA vs. AA |  | 1.931 (1.054-3.539) | 0.033 | F | 0.15 | 0.927 | 0.00% |  |  |  |  |  |  |  |  | 1.000 | 0.877 |
|  |  | CA+AA vs. CC |  | 0.711 (0.497-1.016) | 0.061 | F | 0.23 | 0.893 | 0.00% |  |  |  |  |  |  |  |  | 1.000 | 0.758 |
|  |  | C vs. A | 2 | 1.376 (1.026-1.845) | 0.033 | F | 0.10 | 0.755 | 0.00% |  |  |  |  |  |  |  |  | 1.000 | NA |
| EFS | rs13181 | AC VS. AA | 3 | 1.178 (0.532-2.610) | 0.686 | R | 4.90 | 0.086 | 59.20% | 0.875 (0.550-1.392) | 0.574 | D Caronia et al. | 8.39 | F | 0.30 | 0.585 | 0.00% | 1.000 | 0.412 |
|  |  | CC VS. AA |  | 1.114 (0.240-5.170) | 0.890 | R | 8.83 | 0.012 | 77.40% | 0.499 (0.231-1.082) | 0.078 | D Caronia et al. | 28.45 | F | 0.11 | 0.743 | 0.00% | 0.296 | 0.042 |
|  | rs11615 | TC VS. TT | 2 | 1.139 (0.294-4.414) | 0.850 | R | 2.80 | 0.094 | 64.30% |  |  |  |  |  |  |  |  | 1.000 | NA |
|  |  | CC VS. TT |  | 1.363 (0.737-2.518) | 0.323 | F | 1.32 | 0.251 | 24.20% |  |  |  |  |  |  |  |  | 1.000 | NA |
|  | rs1799793 | GA VS. GG | 2 | 0.846 (0.275-2.604) | 0.771 | R | 2.67 | 0.102 | 62.60% |  |  |  |  |  |  |  |  | 1.000 | NA |
|  |  | AA VS. GG |  | 0.951 (0.060-14.966) | 0.971 | R | 7.28 | 0.007 | 86.30% |  |  |  |  |  |  |  |  | 1.000 | NA |
|  | rs3212986 | CA VS. CC | 3 | 0.961 (0.622-1.485) | 0.859 | F | 2.89 | 0.235 | 30.90% | 0.826 (0.517-1.321) | 0.426 | D Caronia et al. | 14.01 | F | 0.05 | 0.825 | 0.00% | 1.000 | 0.377 |
|  |  | AA VS. CC |  | 1.035 (0.612-1.751) | 0.898 | F | 3.81 | 0.149 | 47.50% | 1.858 (0.843-4.094) | 0.124 | Ting Hao et al. | 13.42 | F | 0.03 | 0.872 | 0.00% | 1.000 | 0.366 |
