# Supplementary material for: Meta-analysis showing that ERCC1 polymorphism is predictive of osteosarcoma prognosis
Source: Oncotarget. 2017 Jul 19;8(37):62769–79. doi: 10.18632/oncotarget.19370 (PMC5617547; doi:10.18632/oncotarget.19370)
Supplement: Supplementary file 5 [file oncotarget-08-62769-s005.doc]

Supplementary Table 4: Results of rs11615 subgroup analysis

| Index | Genetic models | Subgroups | Number of studies | Test of association | | Test of heterogeneity | | | | Test of association after sensitivity analysis | | | | Test of heterogeneity after sensitivity analysis | | | |
| --- | --- | --- | --- | --- | --- | --- | --- | --- | --- | --- | --- | --- | --- | --- | --- | --- | --- |
|  |  |  |  | HR/OR (95%CI) | P-value | Model | Chi-square | P-value | I² | HR/OR (95%CI) | P-value | Study removed as heterogeneity source | Percentage of removed study(%) | Model | Chi-square | P-value | I² |
| OS | TC vs. TT | C/T | 4 | 1.178 (0.719-1.928) | 0.516 | F | 3.60 | 0.301 | 16.70% | 1.476 (0.855-2.255) | 0.162 | Sun Yongjian et al. | 6.36 | F | 0.08 | 0.959 | 0.00% |
|  |  | T/C | 4 | 0.695 (0.488-0.990) | 0.044 | F | 0.06 | 0.996 | 0.00% |  |  |  |  |  |  |  |  |
|  | CC vs. TT | C/T | 4 | 1.257 (0.471-3.356) | 0.648 | R | 12.91 | 0.005 | 76.80% | 2.035 (1.184-3.497) | 0.010 | Sun Yongjian et al. | 14.27 | F | 0.35 | 0.841 | 0.00% |
|  |  | T/C | 4 | 0.539 (0.324-0.897) | 0.017 | F | 4.89 | 0.180 | 38.70% | 0.376 (0.205-0.688) | 0.002 | Paola Biason et al. | 13.12 | F | 0.16 | 0.923 | 0.00% |
|  | TC vs. CC | C/T | 4 | 0.795 (0.576-1.099) | 0.165 | F | 2.37 | 0.499 | 0.00% |  |  |  |  |  |  |  |  |
|  |  | T/C | 4 | 1.280 (0.777-2.108) | 0.332 | F | 5.91 | 0.116 | 49.20% | 1.781 (1.009-3.143) | 0.046 | Paola Biason et al. | 6.75 | F | 0.23 | 0.891 | 0.00% |
|  | TC+CC vs. TT | C/T | 4 | 1.151 (0.500-2.650) | 0.741 | R | 10.01 | 0.081 | 70.00% | 0.683 (0.512-0.910) | 0.035 | Sun Yongjian et al. | 9.28 | F | 0.18 | 0.915 | 0.00% |
|  |  | T/C | 5 | 0.683 (0.512-0.910) | 0.009 | F | 2.38 | 0.666 | 0.00% |  |  |  |  |  |  |  |  |
|  | T vs. C | C/T | 4 | 0.934 (0.544-1.605) | 0.806 | R | 16.73 | 0.001 | 82.10% | 0.701 (0.547-0.897) | 0.005 | Sun Yongjian et al. | 11.68 | F | 0.43 | 0.808 | 0.00% |
|  |  | T/C | 4 | 1.455 (1.151-1.839) | 0.002 | F | 4.83 | 0.185 | 37.80% | 1.619 (1.256-2.087) | <0.001 | Paola Biason et al. | 10.89 | F | 0.27 | 0.874 | 0.00% |
| Good tumor response | TC vs. TT | T/C | 3 | 1.486 (1.032-2.138) | 0.033 | F | 0.14 | 0.932 | 0.00% |  |  |  |  |  |  |  |  |
|  |  | C/T | 2 | 1.103 (0.537-2.264) | 0.789 | F | 0.85 | 0.356 | 0.00% |  |  |  |  |  |  |  |  |
|  | CC vs. TT | T/C | 3 | 2.659 (1.554-4.548) | <0.001 | F | 0.05 | 0.975 | 0.00% |  |  |  |  |  |  |  |  |
|  |  | C/T | 2 | 1.653 (0.378-7.227) | 0.504 | R | 5.05 | 0.025 | 80.40% |  |  |  |  |  |  |  |  |
|  | TC vs. CC | T/C | 3 | 0.498 (0.296-0.839) | 0.009 | F | 0.61 | 0.739 | 0.00% |  |  |  |  |  |  |  |  |
|  |  | C/T | 2 | 0.727 (0.322-1.642) | 0.443 | R | 2.67 | 0.102 | 62.50% |  |  |  |  |  |  |  |  |
|  | TC+CC vs. TT | T/C | 4 | 1.800 (1.322-2.450) | <0.001 | F | 2.71 | 0.439 | 0.00% |  |  |  |  |  |  |  |  |
|  |  | C/T | 2 | 1.531 (0.427-5.494) | 0.513 | R | 4.05 | 0.044 | 75.30% |  |  |  |  |  |  |  |  |
|  | T vs. C | T/C | 3 | 0.554 (0.437-0.702) | <0.001 | F | 1.45 | 0.484 | 0.00% |  |  |  |  |  |  |  |  |
|  |  | C/T | 2 | 0.660 (0.227-1.921) | 0.446 | R | 9.94 | 0.002 | 89.90% |  |  |  |  |  |  |  |  |
| Poor tumor response | TC vs. TT | T/C | 4 | 0.673 (0.485-0.933) | 0.017 | F | 2.29 | 0.514 | 0.00% |  |  |  |  |  |  |  |  |
|  |  | C/T | 2 | 0.902 (0.440-1.848) | 0.777 | F | 0.83 | 0.362 | 0.00% |  |  |  |  |  |  |  |  |
|  | CC vs. TT | T/C | 4 | 0.443 (0.208-0.944) | 0.035 | R | 7.23 | 0.065 | 58.50% | 0.323 (0.193-0.540) | <0.001 | D Carolina et al. | 8.60 | F | 1.60 | 0.449 | 0.00% |
|  |  | C/T | 2 | 0.607 (0.139-2.639) | 0.505 | R | 5.11 | 0.024 | 80.40% |  |  |  |  |  |  |  |  |
|  | TC vs. CC | T/C | 4 | 1.893 (1.185-3.026) | 0.008 | F | 0.81 | 0.848 | 0.00% |  |  |  |  |  |  |  |  |
|  |  | C/T | 2 | 1.369 (0.605-3.094) | 0.451 | R | 2.62 | 0.106 | 61.80% |  |  |  |  |  |  |  |  |
|  | TC+CC vs. TT | T/C | 4 | 1.573 (0.990-2.500) | 0.055 | F | 0.82 | 0.845 | 0.00% |  |  |  |  |  |  |  |  |
|  |  | C/T | 2 | 1.071 (0.746-1.539) | 0.710 | F | 0.31 | 0.581 | 0.00% |  |  |  |  |  |  |  |  |
|  | T vs. C | T/C | 3 | 1.814 (1.431-2.300) | <0.001 | F | 1.53 | 0.464 | 0.00% |  |  |  |  |  |  |  |  |
|  |  | C/T | 2 | 1.515 (0.526-4.365) | 0.441 | R | 9.84 | 0.002 | 89.80% |  |  |  |  |  |  |  |  |
