# Supplementary material for: Meta-analysis showing that ERCC1 polymorphism is predictive of osteosarcoma prognosis
Source: Oncotarget. 2017 Jul 19;8(37):62769–79. doi: 10.18632/oncotarget.19370 (PMC5617547; doi:10.18632/oncotarget.19370)
Supplement: Supplementary file 6 [file oncotarget-08-62769-s006.doc]

Supplementary Table 5: Subgroup analysis：Assessment method of TR

| Index | Locus | Genetic models | Subgroups | Number of studies | Test of association | | Test of heterogeneity | | | | Test of association after sensitivity analysis | | | | Test of heterogeneity after sensitivity analysis | | | |
| --- | --- | --- | --- | --- | --- | --- | --- | --- | --- | --- | --- | --- | --- | --- | --- | --- | --- | --- |
|  |  |  |  |  | OR (95%CI) | P-value | Model | Chi-square | P-value | I² | OR (95%CI) | P-value | Study removed as heterogeneity source | Percentage of removed study(%) | Model | Chi-square | P-value | I^2^ |
| Good tumor response | rs13181 | AC vs AA | Histology | 4 | 1.162 (0.806-1.676) | 0.421 | F | 1.19 | 0.754 | 0.00% |  |  |  |  |  |  |  |  |
|  |  | CC vs AA | Histology | 4 | 1.167 (0.623-2.186) | 0.630 | F | 2.39 | 0.496 | 0.00% |  |  |  |  |  |  |  |  |
|  |  | AC vs CC | Histology | 4 | 0.889 (0.506-1.564) | 0.683 | F | 0.61 | 0.894 | 0.00% |  |  |  |  |  |  |  |  |
|  |  | AC+CC vs AA | Histology | 5 | 1.276 (0.789-2.065) | 0.321 | R | 8.15 | 0.086 | 50.90% | 1.168 (0.852-1.602) | 0.334 | Sun Yongjian et al. | 12.09 | F | 3.84 | 0.279 | 0.00% |
|  |  | A vs C | Histology | 4 | 0.874 (0.688-1.111) | 0.272 | F | 5.41 | 0.144 | 44.50% | 0.787 (0.608-1.017) | 0.068 | Sun Yongjian et al. | 9.19 | F | 0.47 | 0.789 | 0.00% |
|  | rs11615 | TC vs TT | Histology,T/C | 2 | 1.500 (0.977-2.302) | 0.064 | F | 0.13 | 0.714 | 0.00% |  |  |  |  |  |  |  |  |
|  |  | CC vs TT | Histology,T/C | 2 | 2.702 (1.425-5.121) | 0.002 | F | 0.04 | 0.838 | 0.00% |  |  |  |  |  |  |  |  |
|  |  | TC vs CC | Histology,T/C | 2 | 0.467 (0.248-0.880) | 0.018 | F | 0.48 | 0.487 | 0.00% |  |  |  |  |  |  |  |  |
|  |  | TC+CC vs TT | Histology,T/C | 3 | 1.843 (1.284-2.645) | 0.001 | F | 2.65 | 0.266 | 24.50% | 1.969 (1.353-2.865) | <0.001 | Katja et al. | 4.15 | F | 0.95 | 0.329 | 0.00% |
|  |  | T vs C | Histology,T/C | 2 | 0.532 (0.402-0.706) | <0.001 | F | 1.19 | 0.275 | 15.90% |  |  |  |  |  |  |  |  |
|  | rs1799793 | GA vs GG | Histology | 4 | 1.250 (0.870-1.795) | 0.228 | F | 2.70 | 0.441 | 0.00% |  |  |  |  |  |  |  |  |
|  |  | AA vs GG | Histology | 4 | 1.421 (0.788-2.564) | 0.243 | F | 5.24 | 0.155 | 42.80% | 2.175 (1.063-4.451) | 0.033 | Sun Yongjian et al. | 24.81 | F | 1.03 | 0.597 | 0.00% |
|  |  | GA vs AA | Histology | 4 | 0.699 (0.387-1.261) | 0.234 | F | 1.39 | 0.709 | 0.00% |  |  |  |  |  |  |  |  |
|  |  | GA+AA vs GG | Histology | 5 | 1.296 (0.958-1.753) | 0.093 | F | 7.06 | 0.133 | 43.40% | 1.546 (1.102-2.170) | 0.012 | Sun Yongjian et al. | 16.34 | F | 1.95 | 0.584 | 0.00% |
|  |  | G vs A | Histology | 4 | 0.757 (0.483-1.186) | 0.224 | R | 9.57 | 0.023 | 68.70% | 0.618 (0.467-0.817) | 0.001 | Sun Yongjian et al. | 19.15 | F | 0.69 | 0.707 | 0.00% |
|  | rs3212986 | CA+AA vs CC | Histology | 2 | 1.042 (0.715-1.518) | 0.831 | F | 0.68 | 0.411 | 0.00% |  |  |  |  |  |  |  |  |
| Poor tumor response | rs13181 | AC vs AA | Histology | 5 | 1.158 (0.672-1.997) | 0.597 | R | 10.91 | 0.028 | 63.30% | 0.861 (0.623-1.191) | 0.367 | D Carolina et al. | 8.00 | F | 1.51 | 0.679 | 0.00% |
|  |  | CC vs AA | Histology | 5 | 0.950 (0.579-1.558) | 0.839 | F | 5.58 | 0.233 | 28.30% | 0.834 (0.496-1.404) | 0.495 | D Carolina et al. | 7.33 | F | 3.11 | 0.375 | 3.50% |
|  |  | AC vs CC | Histology | 5 | 1.537 (0.931-2.537) | 0.093 | F | 6.09 | 0.192 | 34.40% | 1.128 (0.642-1.983) | 0.675 | D Carolina et al. | 16.06 | F | 0.62 | 0.892 | 0.00% |
|  |  | AC+CC vs AA | Histology | 5 | 0.836 (0.627-1.114) | 0.221 | F | 4.09 | 0.394 | 2.20% | 0.755 (0.555-1.027) | 0.073 | Sun Yongjian et al. | 9.21 | F | 0.79 | 0.853 | 0.00% |
|  |  | A vs C | Histology | 4 | 1.140 (0.897-1.448) | 0.284 | F | 5.23 | 0.156 | 42.60% | 1.263 (0.977-1.633) | 0.074 | Sun Yongjian et al. | 9.05 | F | 0.46 | 0.795 | 0.00% |
|  | rs11615 | TC vs TT | Histology,T/C | 3 | 0.667 (0.457-0.974) | 0.036 | F | 2.28 | 0.319 | 12.40% | 0.835 (0.490-1.422) | 0.507 | Zhang Q et al. | 30.50 | F | 0.90 | 0.343 | 0.00% |
|  |  | CC vs TT | Histology,T/C | 3 | 0.484 (0.161-1.462) | 0.198 | R | 7.22 | 0.027 | 72.30% | 0.296 (0.159-0.551) | <0.001 | D Carolina et al. | 12.49 | F | 1.36 | 0.244 | 26.40% |
|  |  | TC vs CC | Histology,T/C | 3 | 1.945 (1.130-3.346) | 0.016 | F | 0.77 | 0.68 | 0.00% |  |  |  |  |  |  |  |  |
|  |  | TC+CC vs TT | Histology,T/C | 3 | 1.600 (0.932-2.747) | 0.088 | F | 0.81 | 0.668 | 0.00% |  |  |  |  |  |  |  |  |
|  |  | T vs C | Histology,T/C | 2 | 1.886 (1.423-2.499) | <0.001 | F | 1.28 | 0.257 | 22.10% |  |  |  |  |  |  |  |  |
|  | rs1799793 | GA vs GG | Histology | 5 | 0.840 (0.611-1.156) | 0.285 | F | 4.30 | 0.367 | 6.90% | 0.762 (0.538-1.077) | 0.124 | Sun Yongjian et al. | 12.66 | F | 2.29 | 0.514 | 0.00% |
|  |  | AA vs GG | Histology | 5 | 0.759 (0.319-1.802) | 0.531 | R | 10.55 | 0.032 | 62.10% | 0.583 (0.228-1.489) | 0.259 | Sun Yongjian et al. | 20.08 | R | 6.45 | 0.092 | 53.50% |
|  |  | GA vs AA | Histology | 5 | 1.527 (0.918-2.541) | 0.103 | F | 1.53 | 0.821 | 0.00% |  |  |  |  |  |  |  |  |
|  |  | GA+AA vs GG | Histology | 5 | 1.318 (0.845-2.054) | 0.223 | R | 9.34 | 0.025 | 67.90% | 1.612 (1.217-2.134) | 0.001 | Sun Yongjian et al. | 15.69 | F | 0.65 | 0.724 | 0.00% |
|  |  | G vs A | Histology | 4 | 0.773 (0.576-1.038) | 0.087 | F | 6.77 | 0.149 | 40.90% | 0.654 (0.471-0.908) | 0.011 | Sun Yongjian et al. | 19.26 | F | 1.62 | 0.656 | 0.00% |
|  | rs3212986 | CA vs CC | Histology | 2 | 0.877 (0.552-1.391) | 0.576 | F | 1.61 | 0.204 | 37.90% |  |  |  |  |  |  |  |  |
|  |  | AA vs CC | Histology | 2 | 0.875 (0.124-6.192) | 0.894 | R | 5.10 | 0.024 | 80.40% |  |  |  |  |  |  |  |  |
|  |  | CA vs AA | Histology | 2 | 2.042 (0.997-4.182) | 0.051 | F | 0.07 | 0.792 | 0.00% |  |  |  |  |  |  |  |  |
|  |  | CA+AA vs CC | Histology | 2 | 0.668 (0.426-1.047) | 0.079 | F | 0.03 | 0.862 | 0.00% |  |  |  |  |  |  |  |  |
