# Supplementary material for: Meta-analysis showing that ERCC1 polymorphism is predictive of osteosarcoma prognosis
Source: Oncotarget. 2017 Jul 19;8(37):62769–79. doi: 10.18632/oncotarget.19370 (PMC5617547; doi:10.18632/oncotarget.19370)
Supplement: Supplementary file 7 [file oncotarget-08-62769-s007.doc]

Supplementary Table 6: Subgroup analysis：Confounder adjustment of histology

| Index | Locus | Genetic models | Subgroups | Number of studies | Test of association | | Test of heterogeneity | | | | Test of association after sensitivity analysis | | | | Test of heterogeneity after sensitivity analysis | | | |
| --- | --- | --- | --- | --- | --- | --- | --- | --- | --- | --- | --- | --- | --- | --- | --- | --- | --- | --- |
|  |  |  |  |  | HR/OR (95%CI) | P-value | Model | Chi-square | P-value | I² | OR (95%CI) | P-value | Study removed as heterogeneity source | Percentage of removed study | Model | Chi-square | P-value | I^2^ |
| OS | rs13181 | AC vs AA | Yes | 3 | 0.982 (0.618-1.560) | 0.939 | F | 0.19 | 0.908 | 0.00% |  |  |  |  |  |  |  |  |
|  |  |  | No | 4 | 0.804 (0.557-1.160) | 0.244 | F | 0.26 | 0.967 | 0.00% |  |  |  |  |  |  |  |  |
|  |  | CC vs AA | Yes | 3 | 0.869 (0.379-1.992) | 0.741 | F | 0.32 | 0.456 | 0.00% |  |  |  |  |  |  |  |  |
|  |  |  | No | 4 | 0.681 (0.358-1.296) | 0.242 | F | 0.59 | 0.954 | 0.00% |  |  |  |  |  |  |  |  |
|  |  | AC vs CC | Yes | 3 | 1.174 (0.558-2.470) | 0.672 | F | 0.14 | 0.931 | 0.00% |  |  |  |  |  |  |  |  |
|  |  |  | No | 4 | 1.196 (0.639-2.238) | 0.576 | F | 0.40 | 0.941 | 0.00% |  |  |  |  |  |  |  |  |
|  |  | AC+CC vs AA | Yes | 4 | 0.927 (0.651-1.321) | 0.183 | F | 0.67 | 0.880 | 0.00% |  |  |  |  |  |  |  |  |
|  |  |  | No | 4 | 0.796 (0.569-1.114) | 0.675 | F | 0.30 | 0.961 | 0.00% |  |  |  |  |  |  |  |  |
|  |  | A vs C | Yes | 3 | 1.039 (0.761-1.418) | 0.811 | F | 0.65 | 0.723 | 0.00% |  |  |  |  |  |  |  |  |
|  |  |  | No | 4 | 1.275 (0.978-1.662) | 0.073 | F | 0.38 | 0.945 | 0.00% |  |  |  |  |  |  |  |  |
|  | rs11615 | TC vs TT | No, C/T | 2 | 1.508 (0.790-2.879) | 0.213 | F | 0.07 | 0.792 | 0.00% |  |  |  |  |  |  |  |  |
|  |  |  | No, T/C | 3 | 0.703 (0.451-1.096) | 0.120 | F | 0.05 | 0.975 | 0.00% |  |  |  |  |  |  |  |  |
|  |  |  | Yes, C/T | 2 | 0.808 (0.260-2.507) | 0.712 | R | 2.19 | 0.139 | 54.40% |  |  |  |  |  |  |  |  |
|  |  | CC vs TT | No, C/T | 2 | 2.224 (1.166-4.242) | 0.015 | F | 0.11 | 0.743 | 0.00% |  |  |  |  |  |  |  |  |
|  |  |  | No, T/C | 3 | 0.623 (0.346-1.121) | 0.114 | F | 3.97 | 0.137 | 49.60% | 0.391 (0.185-0.828) | 0.014 | Paola et al. | 13.12 | F | 0.13 | 0.718 | 0.00% |
|  |  |  | Yes, C/T | 2 | 0.695 (0.131-3.694) | 0.669 | R | 6.15 | 0.013 | 83.70% |  |  |  |  |  |  |  |  |
|  |  | TC vs CC | No, C/T | 2 | 0.679 (0.448-1.030) | 0.069 | F | 0.01 | 0.920 | 0.00% |  |  |  |  |  |  |  |  |
|  |  |  | No, T/C | 3 | 1.084 (0.425-2.763) | 0.867 | R | 4.84 | 0.089 | 58.70% | 1.699 (0.824-3.505) | 0.151 | Paola et al. | 8.65 | F | 0.19 | 0.664 | 0.00% |
|  |  |  | Yes, C/T | 2 | 1.009 (0.605-1.683) | 0.973 | F | 0.98 | 0.322 | 0.50% |  |  |  |  |  |  |  |  |
|  |  | TC+CC vs TT | No, C/T | 2 | 1.837 (0.994-3.393) | 0.052 | F | 0.08 | 0.779 | 0.00% |  |  |  |  |  |  |  |  |
|  |  |  | No, T/C | 3 | 0.657 (0.445-0.970) | 0.035 | F | 0.74 | 0.692 | 0.00% |  |  |  |  |  |  |  |  |
|  |  |  | Yes, T/C | 2 | 0.714 (0.467-1.093) | 0.121 | F | 1.56 | 0.211 | 36.10% |  |  |  |  |  |  |  |  |
|  |  |  | Yes, C/T | 2 | 0.705 (0.157-3.168) | 0.648 | R | 5.23 | 0.022 | 80.90% |  |  |  |  |  |  |  |  |
|  |  | T vs C | No, C/T | 2 | 0.666 (0.495-0.894) | 0.007 | F | 0.04 | 0.847 | 0.00% |  |  |  |  |  |  |  |  |
|  |  |  | No, T/C | 3 | 1.316 (0.859-2.015) | 0.207 | R | 4.19 | 0.123 | 52.30% | 1.591 (1.145-2.211) | 0.006 | Paola et al. | 10.89 | F | 0.24 | 0.621 | 0.00% |
|  |  |  | Yes, C/T | 2 | 1.359 (0.460-4.022) | 0.579 | R | 9.48 | 0.002 | 89.50% |  |  |  |  |  |  |  |  |
|  | rs1799793 | GA vs GG | Yes | 3 | 0.970 (0.621-1.514) | 0.893 | F | 0.35 | 0.841 | 0.00% |  |  |  |  |  |  |  |  |
|  |  |  | No | 4 | 0.801 (0.531-1.209) | 0.291 | F | 1.17 | 0.760 | 0.00% |  |  |  |  |  |  |  |  |
|  |  | AA vs GG | Yes | 3 | 0.877(0.398-1.934) | 0.746 | F | 2.46 | 0.292 | 18.80% | 1.154 (0.485-2.748) | 0.746 | Liu Z.F. et al. | 15.61 | F | 0.20 | 0.651 | 0.00% |
|  |  |  | No | 4 | 0.425 (0.235-0.767) | 0.005 | F | 2.93 | 0.403 | 0.00% |  |  |  |  |  |  |  |  |
|  |  | GA vs AA | Yes | 3 | 1.291 (0.611-2.728) | 0.503 | F | 2.15 | 0.342 | 6.80% | 0.963 (0.414-2.242) | 0.930 | Liu Z.F. et al. | 6.62 | F | 0.01 | 0.904 | 0.00% |
|  |  |  | No | 4 | 1.102 (0.579-2.095) | 0.768 | F | 1.69 | 0.640 | 0.00% |  |  |  |  |  |  |  |  |
|  |  | GA+AA vs GG | Yes | 4 | 0.955 (0.675-1.351) | 0.795 | F | 1.52 | 0.813 | 0.00% |  |  |  |  |  |  |  |  |
|  |  |  | No | 4 | 0.729 (0.516-1.030) | 0.073 | F | 1.56 | 0.669 | 0.00% |  |  |  |  |  |  |  |  |
|  |  | G vs A | Yes | 3 | 1.085 (0.802-1.469) | 0.596 | F | 3.05 | 0.217 | 34.50% | 0.943 (0.663-1.342) | 0.744 | Liu Z.F. et al. | 9.19 | F | 0.73 | 0.392 | 0.00% |
|  |  |  | No | 4 | 1.315 (0.994-1.740) | 0.055 | F | 2.26 | 0.521 | 0.00% |  |  |  |  |  |  |  |  |
|  | rs3212986 | CA vs CC | No | 3 | 0.871 (0.623-1.220) | 0.422 | F | 0.01 | 0.993 | 0.00% |  |  |  |  |  |  |  |  |
|  |  | AA vs CC | No | 3 | 0.961 (0.545-1.694) | 0.890 | F | 2.59 | 0.274 | 22.70% | 0.680 (0.335-1.381) | 0.286 | Paola et al. | 28.85 | F | 0.04 | 0.839 | 0.00% |
|  |  | CA vs AA | No | 3 | 1.397 (0.741-2.634) | 0.301 | F | 0.07 | 0.966 | 0.00% |  |  |  |  |  |  |  |  |
|  |  | CA+AA vs CC | Yes | 2 | 0.850 (0.551-1.313) | 0.464 | F | 0.06 | 0.810 | 0.00% |  |  |  |  |  |  |  |  |
|  |  |  | No | 3 | 0.862 (0.606-1.226) | 0.410 | F | 3.47 | 0.176 | 42.40% | 0.744 (0.506-1.093) | 0.132 | Paola et al. | 9.86 | F | 0.00 | 0.974 | 0.00% |
|  |  | C vs A | No | 3 | 1.181 (0.898-1.551) | 0.234 | F | 3.56 | 0.168 | 43.90% | 1.325 (0.983-1.787) | 0.064 | Paola et al. | 11.09 | F | 0.01 | 0.924 | 0.00% |
| Good tumor response | rs13181 | AC vs AA | Yes | 4 | 1.235 (0.857-1.779) | 0.257 | F | 1.24 | 0.743 | 0.00% |  |  |  |  |  |  |  |  |
|  |  |  | No | 2 | 1.136 (0.698-1.849) | 0.607 | F | 0.02 | 0.888 | 0.00% |  |  |  |  |  |  |  |  |
|  |  | CC vs AA | Yes | 4 | 1.496 (0.772-2.897) | 0.233 | F | 3.93 | 0.269 | 23.60% | 2.058 (0.977-4.337) | 0.058 | Sun Yongjian et al. | 12.10 | F | 0.62 | 0.733 | 0.00% |
|  |  |  | No | 2 | 1.364 (0.639-2.912) | 0.422 | F | 0.11 | 0.743 | 0.00% |  |  |  |  |  |  |  |  |
|  |  | AC vs CC | Yes | 4 | 0.768 (0.420-1.405) | 0.392 | F | 1.25 | 0.742 | 0.00% |  |  |  |  |  |  |  |  |
|  |  |  | No | 2 | 0.818 (0.376-1.780) | 0.613 | F | 0.12 | 0.734 | 0.00% |  |  |  |  |  |  |  |  |
|  |  | AC+CC vs AA | Yes | 5 | 1.280 (0.937-1.748) | 0.121 | F | 8.01 | 0.091 | 50.00% | 1.472 (1.049-2.066) | 0.025 | Sun Yongjian et al. | 9.07 | F | 3.73 | 0.292 | 19.50% |
|  |  |  | No | 2 | 1.226 (0.774-1.942) | 0.385 | F | 0.24 | 0.625 | 0.00% |  |  |  |  |  |  |  |  |
|  |  | A vs C | Yes | 4 | 0.839 (0.657-1.070) | 0.157 | F | 5.62 | 0.131 | 46.60% | 0.747 (0.575-0.970) | 0.029 | Sun Yongjian et al. | 9.19 | F | 0.05 | 0.975 | 0.00% |
|  |  |  | No | 2 | 0.826 (0.579-1.177) | 0.290 | F | 0.28 | 0.595 | 0.00% |  |  |  |  |  |  |  |  |
|  | rs11615 | TC vs TT | No, T/C | 2 | 1.407 (0.878-2.254) | 0.156 | F | 0.01 | 0.906 | 0.00% |  |  |  |  |  |  |  |  |
|  |  |  | Yes, C/T | 2 | 1.103 (0.537-2.264) | 0.789 | F | 0.85 | 0.356 | 0.00% |  |  |  |  |  |  |  |  |
|  |  | CC vs TT | No, T/C | 2 | 2.534 (1.277-5.029) | 0.008 | F | 0.00 | 0.978 | 0.00% |  |  |  |  |  |  |  |  |
|  |  |  | Yes, C/T | 2 | 1.653 (0.378-7.227) | 0.504 | R | 5.05 | 0.025 | 80.20% |  |  |  |  |  |  |  |  |
|  |  | TC vs CC | No, T/C | 2 | 0.575 (0.305-1.086) | 0.088 | F | 0.00 | 0.979 | 0.00% |  |  |  |  |  |  |  |  |
|  |  |  | Yes, C/T | 2 | 0.727 (0.322-1.642) | 0.443 | R | 2.67 | 0.102 | 62.50% |  |  |  |  |  |  |  |  |
|  |  | TC+CC vs TT | Yes, T/C | 2 | 1.617 (0.583-4.486) | 0.356 | R | 2.23 | 0.135 | 55.20% |  |  |  |  |  |  |  |  |
|  |  |  | No, T/C | 2 | 1.642 (1.093-2.466) | 0.017 | F | 0.02 | 0.895 | 0.00% |  |  |  |  |  |  |  |  |
|  |  |  | Yes, C/T | 2 | 1.531 (0.427-5.494) | 0.513 | R | 4.05 | 0.044 | 75.30% |  |  |  |  |  |  |  |  |
|  |  | T vs C | No, T/C | 2 | 0.621 (0.459-0.839) | 0.002 | F | 0.01 | 0.917 | 0.00% |  |  |  |  |  |  |  |  |
|  |  |  | Yes, C/T | 2 | 0.660 (0.227-1.921) | 0.446 | R | 9.94 | 0.002 | 89.90% |  |  |  |  |  |  |  |  |
|  | rs1799793 | GA vs GG | Yes | 3 | 1.191 (0.778-1.822) | 0.421 | F | 2.52 | 0.284 | 20.50% | 1.393 (0.859-2.261) | 0.179 | Sun Yongjian et al. | 13.34 | F | 0.74 | 0.389 | 0.00% |
|  |  |  | No | 2 | 1.333 (0.805-2.207) | 0.264 | F | 0.07 | 0.793 | 0.00% |  |  |  |  |  |  |  |  |
|  |  | AA vs GG | Yes | 3 | 1.352 (0.451-4.053) | 0.590 | R | 4.25 | 0.119 | 53.00% | 2.161 (0.803-5.819) | 0.127 | Sun Yongjian et al. | 23.91 | F | 1.03 | 0.31 | 3.00% |
|  |  |  | No | 2 | 1.935 (0.915-4.091) | 0.084 | F | 0.11 | 0.735 | 0.00% |  |  |  |  |  |  |  |  |
|  |  | GA vs AA | Yes | 3 | 0.726 (0.350-1.507) | 0.390 | F | 1.36 | 0.507 | 0.00% |  |  |  |  |  |  |  |  |
|  |  |  | No | 2 | 0.692 (0.335-1.428) | 0.319 | F | 0.03 | 0.861 | 0.00% |  |  |  |  |  |  |  |  |
|  |  | GA+AA vs GG | Yes | 4 | 1.170 (0.667-2.052) | 0.583 | R | 6.27 | 0.099 | 52.10% | 1.508(0.992-2.293) | 0.055 | Sun Yongjian et al. | 17.35 | F | 1.91 | 0.385 | 0.00% |
|  |  |  | No | 2 | 1.491 (0.982-2.266) | 0.061 | F | 0.17 | 0.682 | 0.00% |  |  |  |  |  |  |  |  |
|  |  | G vs A | Yes | 3 | 0.817 (0.440-1.516) | 0.521 | R | 8.39 | 0.015 | 76.20% | 0.623 (0.437-0.888) | 0.009 | Sun Yongjian et al. | 19.95 | F | 0.69 | 0.407 | 0.00% |
|  |  |  | No | 2 | 0.664 (0.477-0.925) | 0.015 | F | 0.28 | 0.596 | 0.00% |  |  |  |  |  |  |  |  |
|  | rs3212986 | CA+AA vs CC | Yes | 2 | 1.562 (0.978-2.495) | 0.062 | F | 0.49 | 0.482 | 0.00% |  |  |  |  |  |  |  |  |
| Poor tumor response | rs13181 | AC vs AA | Yes | 4 | 1.306 (0.621-2.746) | 0.481 | R | 10.81 | 0.013 | 72.30% | 0.844 (0.577-1.234) | 0.381 | D Carolina et al. | 10.63 | F | 1.47 | 0.479 | 0.00% |
|  |  |  | No | 2 | 0.854 (0.519-1.405) | 0.534 | F | 0.12 | 0.735 | 0.00% |  |  |  |  |  |  |  |  |
|  |  | CC vs AA | Yes | 4 | 1.003 (0.558-1.801) | 0.992 | F | 5.47 | 0.141 | 45.10% | 0.836 (0.446-1.570) | 0.578 | D Carolina et al. | 8.37 | F | 3.11 | 0.211 | 35.70% |
|  |  |  | No | 3 | 0.705 (0.334-1.486) | 0.358 | F | 0.34 | 0.558 | 0.00% |  |  |  |  |  |  |  |  |
|  |  | AC vs CC | Yes | 4 | 1.725 (0.965-3.084) | 0.066 | F | 5.50 | 0.138 | 45.50% | 1.142 (0.575-2.269) | 0.705 | D Carolina et al. | 18.22 | F | 0.61 | 0.735 | 0.00% |
|  |  |  | No | 2 | 1.219 (0.559-2.656) | 0.619 | F | 0.11 | 0.742 | 0.00% |  |  |  |  |  |  |  |  |
|  |  | AC+CC vs AA | Yes | 4 | 0.818 (0.586-1.141) | 0.237 | F | 4.03 | 0.259 | 25.50% | 0.705 (0.489-1.016) | 0.061 | Sun Yongjian et al. | 11.22 | F | 0.33 | 0.848 | 0.00% |
|  |  |  | No | 2 | 0.819 (0.517-1.299) | 0.397 | F | 0.24 | 0.628 | 0.00% |  |  |  |  |  |  |  |  |
|  |  | A vs C | Yes | 3 | 1.068 (0.664-1.718) | 0.787 | R | 5.22 | 0.074 | 61.70% | 1.340 (0.980-1.834) | 0.067 | Sun Yongjian et al. | 12.93 | F | 0.04 | 0.835 | 0.00% |
|  |  |  | No | 2 | 1.207 (0.847-1.720) | 0.297 | F | 0.29 | 0.590 | 0.00% |  |  |  |  |  |  |  |  |
|  | rs11615 | TC vs TT | Yes, T/C | 2 | 0.739 (0.304-1.795) | 0.504 | R | 2.14 | 0.144 | 53.20% |  |  |  |  |  |  |  |  |
|  |  |  | No, T/C | 2 | 0.711 (0.458-1.104) | 0.129 | F | 0.02 | 0.900 | 0.00% |  |  |  |  |  |  |  |  |
|  |  |  | Yes, C/T | 2 | 0.902 (0.440-1.848) | 0.777 | F | 0.83 | 0.362 | 0.00% |  |  |  |  |  |  |  |  |
|  |  | CC vs TT | Yes, T/C | 2 | 0.566 (0.067-4.805) | 0.602 | R | 7.22 | 0.007 | 86.10% |  |  |  |  |  |  |  |  |
|  |  |  | No, T/C | 2 | 0.406 (0.218-0.756) | 0.005 | F | 0.01 | 0.907 | 0.00% |  |  |  |  |  |  |  |  |
|  |  |  | Yes, C/T | 2 | 0.607 (0.139-2.639) | 0.505 | R | 5.11 | 0.024 | 80.40% |  |  |  |  |  |  |  |  |
|  |  | TC vs CC | Yes, T/C | 2 | 2.101 (1.051-4.199) | 0.036 | F | 0.65 | 0.421 | 0.00% |  |  |  |  |  |  |  |  |
|  |  |  | No, T/C | 2 | 1.734 (0.917-3.278) | 0.090 | F | 0.00 | 0.979 | 0.00% |  |  |  |  |  |  |  |  |
|  |  |  | Yes, C/T | 2 | 1.369 (0.605-3.094) | 0.451 | R | 2.62 | 0.106 | 61.80% |  |  |  |  |  |  |  |  |
|  |  | TC+CC vs TT | Yes, T/C | 2 | 1.690 (0.835-3.418) | 0.145 | F | 0.75 | 0.387 | 0.00% |  |  |  |  |  |  |  |  |
|  |  |  | No, T/C | 2 | 1.489 (0.805-2.755) | 0.204 | F | 0.00 | 0.983 | 0.00% |  |  |  |  |  |  |  |  |
|  |  |  | Yes, C/T | 2 | 1.071 (0.746-1.539) | 0.710 | F | 0.31 | 0.581 | 0.00% |  |  |  |  |  |  |  |  |
|  |  | T vs C | No, T/C | 2 | 1.613 (1.192-2.182) | 0.002 | F | 0.02 | 0.889 | 0.00% |  |  |  |  |  |  |  |  |
|  |  |  | Yes, C/T | 2 | 1.515 (0.526-4.365) | 0.441 | R | 9.84 | 0.002 | 89.80% |  |  |  |  |  |  |  |  |
|  | rs1799793 | GA vs GG | Yes | 3 | 0.826 (0.558-1.223) | 0.340 | F | 2.72 | 0.257 | 26.50% | 0.697 (0.445-1.091) | 0.114 | Sun Yongjian et al. | 12.66 | F | 0.36 | 0.547 | 0.00% |
|  |  |  | No | 3 | 0.841 (0.550-1.287) | 0.425 | F | 1.59 | 0.452 | 0.00% |  |  |  |  |  |  |  |  |
|  |  | AA vs GG | Yes | 3 | 0.617 (0.185-2.053) | 0.431 | R | 6.01 | 0.050 | 66.70% | 0.340 (0.133-0.867) | 0.024 | Sun Yongjian et al. | 20.08 | F | 0.29 | 0.593 | 0.00% |
|  |  |  | No | 3 | 0.805 (0.299-2.169) | 0.668 | R | 4.69 | 0.096 | 57.40% | 0.519 (0.267-1.009) | 0.053 | D Carolina et al. | 11.45 | F | 0.13 | 0.714 | 0.00% |
|  |  | GA vs AA | Yes | 3 | 1.387 (0.665-2.895) | 0.384 | F | 1.36 | 0.507 | 0.00% |  |  |  |  |  |  |  |  |
|  |  |  | No | 3 | 1.567 (0.871-2.817) | 0.134 | F | 0.15 | 0.929 | 0.00% |  |  |  |  |  |  |  |  |
|  |  | GA+AA vs GG | Yes | 3 | 0.812 (0.430-1.533) | 0.521 | R | 5.69 | 0.058 | 64.90% | 0.625 (0.407-0.960) | 0.032 | Sun Yongjian et al. | 16.58 | F | 0.67 | 0.412 | 0.00% |
|  |  |  | No | 3 | 0.711 (0.481-1.049) | 0.086 | F | 0.85 | 0.653 | 0.00% |  |  |  |  |  |  |  |  |
|  |  | G vs A | Yes | 3 | 1.224 (0.665-2.254) | 0.517 | R | 8.20 | 0.017 | 75.60% | 1.601 (1.123-2.283) | 0.009 | Sun Yongjian et al. | 19.26 | F | 0.64 | 0.423 | 0.00% |
|  |  |  | No | 2 | 1.506 (1.080-2.100) | 0.016 | F | 0.24 | 0.624 | 0.00% |  |  |  |  |  |  |  |  |
|  | rs3212986 | CA vs CC | No | 2 | 1.019 (0.596-1.742) | 0.944 | F | 1.01 | 0.315 | 0.80% |  |  |  |  |  |  |  |  |
|  |  | AA vs CC | No | 2 | 1.080 (0.232-5.030) | 0.922 | R | 3.02 | 0.082 | 66.90% |  |  |  |  |  |  |  |  |
|  |  | CA vs AA | No | 2 | 1.787 (0.856-3.733) | 0.122 | F | 0.02 | 0.888 | 0.00% |  |  |  |  |  |  |  |  |
|  |  | CA+AA vs CC | No | 2 | 0.668 (0.426-1.047) | 0.079 | F | 0.03 | 0.863 | 0.00% |  |  |  |  |  |  |  |  |
