# Supplementary material for: Meta-analysis showing that ERCC1 polymorphism is predictive of osteosarcoma prognosis
Source: Oncotarget. 2017 Jul 19;8(37):62769–79. doi: 10.18632/oncotarget.19370 (PMC5617547; doi:10.18632/oncotarget.19370)
Supplement: Supplementary file 8 [file oncotarget-08-62769-s008.doc]

Supplementary Table 7: Subgroup analysis：Confounder adjustment of metastasis

| Index | Locus | Genetic models | Subgroups | Number of studies | Test of association | | Test of heterogeneity | | | | Test of association after sensitivity analysis | | | | Test of heterogeneity after sensitivity analysis | | | |
| --- | --- | --- | --- | --- | --- | --- | --- | --- | --- | --- | --- | --- | --- | --- | --- | --- | --- | --- |
|  |  |  |  |  | HR/OR (95%CI) | P-value | Model | Chi-square | P-value | I² | OR (95%CI) | P-value | Study removed as heterogeneity source | Percentage of removed study | Model | Chi-square | P-value | I^2^ |
| OS | rs13181 | AC vs AA | Yes | 3 | 0.851 (0.538-1.346) | 0.490 | F | 0.60 | 0.743 | 0.00% |  |  |  |  |  |  |  |  |
|  |  |  | No | 4 | 0.880 (0.609-1.273) | 0.498 | F | 0.29 | 0.962 | 0.00% |  |  |  |  |  |  |  |  |
|  |  | CC vs AA | Yes | 3 | 0.835 (0.399-1.750) | 0.633 | F | 0.34 | 0.846 | 0.00% |  |  |  |  |  |  |  |  |
|  |  |  | No | 4 | 0.675 (0.335-1.359) | 0.271 | F | 0.61 | 0.893 | 0.00% |  |  |  |  |  |  |  |  |
|  |  | AC vs CC | Yes | 3 | 1.071 (0.517-2.219) | 0.853 | F | 0.00 | 0.998 | 0.00% |  |  |  |  |  |  |  |  |
|  |  |  | No | 4 | 1.283 (0.679-2.425) | 0.442 | F | 0.40 | 0.939 | 0.00% |  |  |  |  |  |  |  |  |
|  |  | AC+CC vs AA | Yes | 3 | 0.871 (0.587-1.293) | 0.494 | F | 0.99 | 0.608 | 0.00% |  |  |  |  |  |  |  |  |
|  |  |  | No | 5 | 0.846 (0.621-1.153) | 0.290 | F | 0.33 | 0.933 | 0.00% |  |  |  |  |  |  |  |  |
|  |  | A vs C | Yes | 3 | 1.118 (0.815-1.533) | 0.489 | F | 1.06 | 0.588 | 0.00% |  |  |  |  |  |  |  |  |
|  |  |  | No | 4 | 1.206 (0.928-1.568) | 0.162 | F | 0.80 | 0.850 | 0.00% |  |  |  |  |  |  |  |  |
|  | rs11615 | TC vs TT | Yes C/T | 4 | 1.178 (0.719-1.928) | 0.514 | F | 3.60 | 0.308 | 16.70% | 1.476 (0.855-2.550) | 0.162 | SunYongjian et al. | 6.36 | F | 0.08 | 0.959 | 0.00% |
|  |  |  | No,T/C | 3 | 0.679 (0.450-1.026) | 0.066 | F | 0.01 | 0.993 | 0.00% |  |  |  |  |  |  |  |  |
|  |  | CC vs TT | Yes C/T | 4 | 1.257 (0.471-3.356) | 0.648 | R | 12.91 | 0.005 | 76.80% | 2.035 (1.184-3.497) | 0.010 | SunYongjian et al. | 13.21 | F | 0.35 | 0.841 | 0.00% |
|  |  |  | No,T/C | 3 | 0.558 (0.217-1.435) | 0.226 | R | 4.55 | 0.103 | 56.10% | 0.338 (0.151-0.758) | 0.008 | Paola et al. | 12.91 | F | 0.01 | 0.916 | 0.00% |
|  |  | TC vs CC | Yes C/T | 4 | 0.795 (0.576-1.099) | 0.165 | F | 2.37 | 0.499 | 0.00% |  |  |  |  |  |  |  |  |
|  |  |  | No,T/C | 3 | 1.187 (0.428-3.292) | 0.742 | R | 5.74 | 0.057 | 65.20% | 1.982 (0.961-4.088) | 0.064 | Paola et al. | 8.65 | F | 0.01 | 0.911 | 0.00% |
|  |  | TC+CC vs TT | Yes C/T | 4 | 1.151 (0.500-2.650) | 0.742 | R | 10.01 | 0.018 | 70.00% | 1.741 (1.039-2.918) | 0.034 | SunYongjian et al. | 9.28 | F | 0.18 | 0.915 | 0.00% |
|  |  |  | No,T/C | 4 | 0.690 (0.496-0.961) | 0.028 | F | 2.37 | 0.500 | 0.00% |  |  |  |  |  |  |  |  |
|  |  | T vs C | Yes C/T | 4 | 0.934 (0.544-1.605) | 0.806 | R | 16.73 | 0.001 | 82.10% | 1.376 (0.882-2.146) | 0.005 | SunYongjian et al. | 11.68 | F | 0.43 | 0.808 | 0.00% |
|  |  |  | No,T/C | 3 | 1.376 (0.882-2.146) | 0.160 | R | 4.82 | 0.090 | 58.50% | 1.695 (1.240-2.316) | 0.001 | Paola et al. | 10.89 | F | 0.03 | 0.872 | 0.00% |
|  | rs1799793 | GA vs GG | Yes | 4 | 0.958 (0.596-1.538) | 0.858 | F | 0.45 | 0.797 | 0.00% |  |  |  |  |  |  |  |  |
|  |  |  | No | 4 | 0.822 (0.556-1.217) | 0.329 | F | 1.21 | 0.751 | 0.00% |  |  |  |  |  |  |  |  |
|  |  | AA vs GG | Yes | 4 | 0.500 (0.165-1.519) | 0.222 | R | 5.35 | 0.069 | 62.60% | 0.284 (0.126-0.641) | 0.002 | SunYongjian et al. | 15.93 | F | 0.65 | 0.421 | 0.00% |
|  |  |  | No | 4 | 0.641 (0.329-1.250) | 0.192 | F | 1.72 | 0.631 | 0.00% |  |  |  |  |  |  |  |  |
|  |  | GA vs AA | Yes | 4 | 1.041 (0.503-2.157) | 0.913 | F | 1.73 | 0.420 | 0.00% |  |  |  |  |  |  |  |  |
|  |  |  | No | 4 | 1.303 (0.676-2.512) | 0.429 | F | 2.00 | 0.573 | 0.00% |  |  |  |  |  |  |  |  |
|  |  | GA+AA vs GG | Yes | 4 | 0.893 (0.609-1.312) | 0.565 | F | 1.55 | 0.461 | 0.00% |  |  |  |  |  |  |  |  |
|  |  |  | No | 5 | 0.796 (0.579-1.093) | 0.158 | F | 2.49 | 0.646 | 0.00% |  |  |  |  |  |  |  |  |
|  |  | G vs A | Yes | 4 | 1.087 (0.796-1.484) | 0.599 | F | 2.82 | 0.244 | 29.00% | 1.247 (0.863-1.803) | 0.239 | SunYongjian et al. | 12.44 | F | 0.94 | 0.333 | 0.00% |
|  |  |  | No | 4 | 1.303 (0.991-1.713) | 0.058 | F | 2.60 | 0.458 | 0.00% |  |  |  |  |  |  |  |  |
|  | rs3212986 | CA vs CC | No | 3 | 0.850 (0.594-1.216) | 0.373 | F | 0.00 | 0.998 | 0.00% |  |  |  |  |  |  |  |  |
|  |  | AA vs CC | No | 3 | 1.048 (0.549-2.002) | 0.887 | F | 2.29 | 0.318 | 12.60% | 0.658 (0.271-1.598) | 0.355 | Paola et al. | 28.85 | F | 0.03 | 0.867 | 0.00% |
|  |  | CA vs AA | No | 3 | 1.251 (0.615-2.542) | 0.537 | F | 0.06 | 0.970 | 0.00% |  |  |  |  |  |  |  |  |
|  |  | CA+AA vs CC | No | 4 | 0.919 (0.660-1.281) | 0.618 | F | 3.01 | 0.390 | 0.30% | 0.919 (0.660-1.281) | 0.270 | Paola et al. | 9.86 | F | 0.16 | 0.923 | 0.00% |
|  |  | C vs A | No | 3 | 1.097 (0.826-1.457) | 0.522 | F | 2.86 | 0.239 | 30.20% | 1.226 (0.897-1.675) | 0.201 | Paola et al. | 11.09 | F | 0.09 | 0.767 | 0.00% |
| Good tumor response | rs13181 | AC vs AA | Yes | 3 | 1.118 (0.727-1.720) | 0.611 | F | 1.01 | 0.604 | 0.00% |  |  |  |  |  |  |  |  |
|  |  |  | No | 3 | 1.272 (0.855-1.892) | 0.236 | F | 0.14 | 0.932 | 0.00% |  |  |  |  |  |  |  |  |
|  |  | CC vs AA | Yes | 3 | 1.273 (0.635-2.551) | 0.496 | F | 3.65 | 0.161 | 45.20% | 1.742 (0.787-3.857) | 0.171 | SunYongjian et al. | 12.10 | F | 1.11 | 0.291 | 10.20% |
|  |  |  | No | 3 | 1.634 (0.800-3.340) | 0.178 | F | 0.18 | 0.916 | 0.00% |  |  |  |  |  |  |  |  |
|  |  | AC vs CC | Yes | 3 | 0.838 (0.420-1.673) | 0.377 | F | 1.30 | 0.523 | 0.00% |  |  |  |  |  |  |  |  |
|  |  |  | No | 3 | 0.743 (0.385-1.435) | 0.616 | F | 0.02 | 0.991 | 0.00% |  |  |  |  |  |  |  |  |
|  |  | AC+CC vs AA | Yes | 3 | 1.572 (1.086-2.277) | 0.888 | F | 2.38 | 0.305 | 15.80% | 1.183 (0.790-1.771) | 0.416 | Katja et al. | 2.43 | F | 0.05 | 0.822 | 0.00% |
|  |  |  | No | 4 | 1.026 (0.716-1.471) | 0.017 | F | 3.27 | 0.352 | 8.30% | 1.454 (0.994-2.125) | 0.054 | SunYongjian et al. | 10.49 | F | 0.00 | 0.999 | 0.00% |
|  |  | A vs C | Yes | 3 | 1.003 (0.647-1.554) | 0.990 | R | 4.25 | 0.119 | 53.00% | 0.826 (0.596-1.146) | 0.253 | SunYongjian et al. | 10.00 | F | 0.22 | 0.637 | 0.00% |
|  |  |  | No | 3 | 0.739 (0.561-0.973) | 0.031 | F | 0.05 | 0.978 | 0.00% |  |  |  |  |  |  |  |  |
|  | rs11615 | TC vs TT | No,T/C | 2 | 1.543 (0.993-2.397) | 0.054 | F | 0.05 | 0.819 | 0.00% |  |  |  |  |  |  |  |  |
|  |  |  | Yes C/T | 2 | 1.103 (0.537-2.264) | 0.789 | F | 0.85 | 0.356 | 0.00% |  |  |  |  |  |  |  |  |
|  |  | CC vs TT | No,T/C | 2 | 2.731 (1.426-5.232) | 0.002 | F | 0.03 | 0.864 | 0.00% |  |  |  |  |  |  |  |  |
|  |  |  | Yes C/T | 2 | 1.653 (0.378-7.227) | 0.504 | R | 5.05 | 0.025 | 80.20% |  |  |  |  |  |  |  |  |
|  |  | TC vs CC | No,T/C | 2 | 0.458 (0.240-0.876) | 0.018 | F | 0.43 | 0.513 | 0.00% |  |  |  |  |  |  |  |  |
|  |  |  | Yes C/T | 2 | 0.727 (0.322-1.642) | 0.443 | R | 2.67 | 0.102 | 62.50% |  |  |  |  |  |  |  |  |
|  |  | TC+CC vs TT | No,T/C | 3 | 1.845 (1.198-2.841) | 0.005 | F | 2.47 | 0.291 | 18.90% | 2.035 (1.386-2.988) | <0.001 | Katja et al. | 5.67 | F | 0.65 | 0.418 | 0.00% |
|  |  |  | Yes C/T | 2 | 1.531 (0.427-5.494) | 0.513 | R | 4.05 | 0.044 | 75.30% |  |  |  |  |  |  |  |  |
|  |  | T vs C | No,T/C | 2 | 0.520 (0.389-0.695) | <0.001 | F | 0.89 | 0.344 | 0.00% |  |  |  |  |  |  |  |  |
|  |  |  | Yes C/T | 2 | 0.660 (0.227-1.921) | 0.446 | R | 9.94 | 0.002 | 89.90% |  |  |  |  |  |  |  |  |
|  | rs1799793 | GA vs GG | Yes | 2 | 1.088 (0.630-1.879) | 0.762 | F | 1.51 | 0.219 | 33.90% |  |  |  |  |  |  |  |  |
|  |  |  | No | 3 | 1.345 (0.898-2.016) | 0.151 | F | 0.81 | 0.667 | 0.00% |  |  |  |  |  |  |  |  |
|  |  | AA vs GG | Yes | 2 | 1.128 (0.307-4.146) | 0.856 | R | 3.15 | 0.076 | 68.20% |  |  |  |  |  |  |  |  |
|  |  |  | No | 3 | 1.932 (0.930-4.012) | 0.077 | F | 1.14 | 0.566 | 0.00% |  |  |  |  |  |  |  |  |
|  |  | GA vs AA | Yes | 2 | 0.852 (0.404-1.798) | 0.158 | F | 0.62 | 0.430 | 0.00% |  |  |  |  |  |  |  |  |
|  |  |  | No | 3 | 0.600 (0.295-1.219) | 0.674 | F | 0.33 | 0.849 | 0.00% |  |  |  |  |  |  |  |  |
|  |  | GA+AA vs GG | Yes | 2 | 1.043 (0.427-2.552) | 0.926 | R | 4.10 | 0.043 | 75.60% |  |  |  |  |  |  |  |  |
|  |  |  | No | 4 | 1.459 (1.033-2.060) | 0.032 | F | 1.98 | 0.576 | 0.00% |  |  |  |  |  |  |  |  |
|  |  | G vs A | Yes | 2 | 0.957 (0.389-2.355) | 0.923 | R | 6.70 | 0.010 | 85.10% |  |  |  |  |  |  |  |  |
|  |  |  | No | 3 | 0.659 (0.495-0.876) | 0.004 | F | 0.96 | 0.620 | 0.00% |  |  |  |  |  |  |  |  |
| Poor tumor response | rs13181 | AC vs AA | Yes | 3 | 1.773 (0.650-4.836) | 0.263 | R | 7.40 | 0.025 | 73.00% | 1.036 (0.613-1.750) | 0.895 | D.Carolina et al. | 10.63 | F | 0.60 | 0.44 | 0.00% |
|  |  |  | No | 3 | 0.767 (0.530-1.110) | 0.159 | F | 0.15 | 0.928 | 0.00% |  |  |  |  |  |  |  |  |
|  |  | CC vs AA | Yes | 3 | 1.400 (0.715-2.740) | 0.326 | F | 2.74 | 0.255 | 26.90% | 2.503 (0.944-6.641) | 0.065 | Ji Weiping et al. | 24.79 | F | 0.15 | 0.702 | 0.00% |
|  |  |  | No | 3 | 0.578 (0.307-1.089) | 0.090 | F | 0.08 | 0.962 | 0.00% |  |  |  |  |  |  |  |  |
|  |  | AC vs CC | Yes | 3 | 1.622 (0.510-5.163) | 0.413 | R | 5.82 | 0.054 | 65.70% | 0.946 (0.414-2.162) | 0.896 | D.Carolina et al. | 18.22 | F | 0.29 | 0.589 | 0.00% |
|  |  |  | No | 3 | 1.348 (0.698-2.605) | 0.374 | F | 0.01 | 0.993 | 0.00% |  |  |  |  |  |  |  |  |
|  |  | AC+CC vs AA | Yes | 2 | 1.099 (0.691-1.750) | 0.690 | F | 1.59 | 0.207 | 37.20% |  |  |  |  |  |  |  |  |
|  |  |  | No | 4 | 0.704 (0.505-0.981) | 0.038 | F | 0.33 | 0.954 | 0.00% |  |  |  |  |  |  |  |  |
|  |  | A vs C | Yes | 2 | 0.838 (0.435-1.613) | 0.596 | R | 2.72 | 0.099 | 63.30% |  |  |  |  |  |  |  |  |
|  |  |  | No | 3 | 1.347 (1.022-1.775) | 0.034 | F | 0.05 | 0.976 | 0.00% |  |  |  |  |  |  |  |  |
|  | rs11615 | TC vs TT | No,T/C | 2 | 0.591 (0.391-0.893) | 0.013 | F | 0.38 | 0.539 | 0.00% |  |  |  |  |  |  |  |  |
|  |  |  | Yes,T/C | 2 | 0.835 (0.490-1.422) | 0.507 | F | 0.90 | 0.343 | 0.00% |  |  |  |  |  |  |  |  |
|  |  |  | Yes,C/T | 2 | 0.902 (0.440-1.848) | 0.777 | F | 0.83 | 0.362 | 0.00% |  |  |  |  |  |  |  |  |
|  |  | CC vs TT | No,T/C | 2 | 0.279 (0.147-0.530) | <0.001 | F | 1.04 | 0.307 | 4.10% |  |  |  |  |  |  |  |  |
|  |  |  | Yes,T/C | 2 | 0.791 (0.194-3.222) | 0.744 | R | 3.26 | 0.071 | 69.30% |  |  |  |  |  |  |  |  |
|  |  |  | Yes,C/T | 2 | 0.607 (0.139-2.639) | 0.505 | R | 5.11 | 0.024 | 80.40% |  |  |  |  |  |  |  |  |
|  |  | TC vs CC | No,T/C | 2 | 2.174 (1.136-4.161) | 0.019 | F | 0.41 | 0.524 | 0.00% |  |  |  |  |  |  |  |  |
|  |  |  | Yes,T/C | 2 | 1.629 (0.827-3.207) | 0.158 | F | 0.04 | 0.846 | 0.00% |  |  |  |  |  |  |  |  |
|  |  |  | Yes,C/T | 2 | 1.369 (0.605-3.094) | 0.451 | R | 2.62 | 0.106 | 61.80% |  |  |  |  |  |  |  |  |
|  |  | TC+CC vs TT | No,T/C | 3 | 1.615 (0.927-2.813) | 0.090 | F | 0.79 | 0.673 | 0.00% |  |  |  |  |  |  |  |  |
|  |  |  | Yes,C/T | 2 | 1.071 (0.746-1.539) | 0.710 | F | 0.31 | 0.581 | 0.00% |  |  |  |  |  |  |  |  |
|  |  | T vs C | No,T/C | 2 | 1.939 (1.453-2.589) | <0.001 | F | 0.90 | 0.342 | 0.00% |  |  |  |  |  |  |  |  |
|  |  |  | Yes,C/T | 2 | 1.515 (0.526-4.365) | 0.441 | R | 9.84 | 0.002 | 89.80% |  |  |  |  |  |  |  |  |
|  | rs1799793 | GA vs GG | Yes | 3 | 1.017 (0.647-1.601) | 0.941 | F | 2.58 | 0.275 | 22.50% | 1.470 (0.776-2.783) | 0.237 | Ji Weiping et al. | 20.10 | F | 0.01 | 0.936 | 0.00% |
|  |  |  | No | 3 | 0.727 (0.500-1.056) | 0.094 | F | 0.47 | 0.789 | 0.00% |  |  |  |  |  |  |  |  |
|  |  | AA vs GG | Yes | 3 | 1.246 (0.401-3.875) | 0.704 | R | 6.20 | 0.045 | 67.80% | 2.045 (0.910-4.598) | 0.083 | Ji Weiping et al. | 23.60 | F | 0.56 | 0.453 | 0.00% |
|  |  |  | No | 3 | 0.446 (0.229-0.869) | 0.018 | F | 0.94 | 0.626 | 0.00% |  |  |  |  |  |  |  |  |
|  |  | GA vs AA | Yes | 3 | 1.376 (0.757-2.502) | 0.296 | F | 1.07 | 0.586 | 0.00% |  |  |  |  |  |  |  |  |
|  |  |  | No | 3 | 1.681 (0.822-3.435) | 0.154 | F | 0.32 | 0.851 | 0.00% |  |  |  |  |  |  |  |  |
|  |  | GA+AA vs GG | Yes | 3 | 0.987 (0.522-1.867) | 0.969 | R | 4.31 | 0.116 | 53.60% | 1.431 (0.803-2.552) | 0.224 | Ji Weiping et al. | 21.76 | F | 0.22 | 0.642 | 0.00% |
|  |  |  | No | 3 | 0.658 (0.464-0.934) | 0.019 | F | 0.84 | 0.656 | 0.00% |  |  |  |  |  |  |  |  |
|  |  | G vs A | Yes | 2 | 1.047 (0.432-2.538) | 0.920 | R | 6.48 | 0.011 | 84.60% |  |  |  |  |  |  |  |  |
|  |  |  | No | 3 | 1.520 (1.142-2.022) | 0.004 | F | 0.88 | 0.644 | 0.00% |  |  |  |  |  |  |  |  |
|  | rs3212986 | CA+AA vs CC | No | 3 | 0.723 (0.496-1.054) | 0.092 | F | 0.15 | 0.701 | 0.00% |  |  |  |  |  |  |  |  |
