# Supplementary material for: Meta-analysis showing that ERCC1 polymorphism is predictive of osteosarcoma prognosis
Source: Oncotarget. 2017 Jul 19;8(37):62769–79. doi: 10.18632/oncotarget.19370 (PMC5617547; doi:10.18632/oncotarget.19370)
Supplement: Supplementary file 9 [file oncotarget-08-62769-s009.doc]

Supplementary Table 8: Subgroup analysis：Confounder adjustment of tumor location

| Index | Locus | Genetic models | Subgroups | Number of studies | Test of association | | Test of heterogeneity | | | | Test of association after sensitivity analysis | | | | Test of heterogeneity after sensitivity analysis | | | |
| --- | --- | --- | --- | --- | --- | --- | --- | --- | --- | --- | --- | --- | --- | --- | --- | --- | --- | --- |
|  |  |  |  |  | HR/OR (95%CI) | P-value | Model | Chi-square | P-value | I² | OR (95%CI) | P-value | Study removed as heterogeneity source | Percentage of removed study(%) | Model | Chi-square | P-value | I^2^ |
| OS | rs13181 | AC vs AA | Yes | 5 | 0.895 (0.635-1.261) | 0.525 | F | 0.68 | 0.953 | 0.00% |  |  |  |  |  |  |  |  |
|  |  |  | No | 2 | 0.811 (0.479-1.371) | 0.433 | F | 0.12 | 0.729 | 0.00% |  |  |  |  |  |  |  |  |
|  |  | CC vs AA | Yes | 5 | 0.801 (0.428-1.500) | 0.489 | F | 0.43 | 0.980 | 0.00% |  |  |  |  |  |  |  |  |
|  |  |  | No | 2 | 0.651 (0.273-1.553) | 0.333 | F | 0.54 | 0.461 | 0.00% |  |  |  |  |  |  |  |  |
|  |  | AC vs CC | Yes | 5 | 1.153 (0.646-2.056) | 0.630 | F | 0.15 | 0.997 | 0.00% |  |  |  |  |  |  |  |  |
|  |  |  | No | 2 | 1.264 (0.538-2.973) | 0.591 | F | 0.36 | 0.547 | 0.00% |  |  |  |  |  |  |  |  |
|  |  | AC+CC vs AA | Yes | 6 | 0.874 (0.659-1.158) | 0.348 | F | 1.10 | 0.954 | 0.00% |  |  |  |  |  |  |  |  |
|  |  |  | No | 2 | 0.805 (0.497-1.305) | 0.379 | F | 0.15 | 0.697 | 0.00% |  |  |  |  |  |  |  |  |
|  |  | A vs C | Yes | 5 | 1.147 (0.903-1.457) | 0.261 | F | 1.63 | 0.804 | 0.00% |  |  |  |  |  |  |  |  |
|  |  |  | No | 2 | 1.226 (0.842-1.786) | 0.287 | F | 0.28 | 0.599 | 0.00% |  |  |  |  |  |  |  |  |
|  | rs11615 | TC vs TT | Yes C/T | 3 | 1.043 (0.554-1.964) | 0.897 | F | 3.24 | 0.198 | 38.30% | 1.531 (0.715-3.276) | 0.272 | Sun Yongjian et al. | 6.36 | F | 0.07 | 0.798 | 0.00% |
|  |  |  | No,T/C | 2 | 0.729 (0.418-1.273) | 0.267 | F | 0.00 | 0.945 | 0.00% |  |  |  |  |  |  |  |  |
|  |  |  | Yes,T/C | 2 | 0.672 (0.425-1.063) | 0.090 | F | 0.00 | 0.950 | 0.00% |  |  |  |  |  |  |  |  |
|  |  | CC vs TT | Yes C/T | 3 | 1.054 (0.279-3.977) | 0.938 | R | 10.29 | 0.006 | 80.60% | 2.003 (0.944-4.251) | 0.070 | Sun Yongjian et al. | 13.21 | F | 0.34 | 0.558 | 0.00% |
|  |  |  | No,T/C | 2 | 0.745 (0.250-2.218) | 0.597 | R | 2.75 | 0.097 | 63.70% |  |  |  |  |  |  |  |  |
|  |  |  | Yes,T/C | 2 | 0.338 (0.151-0.758) | 0.008 | F | 0.00 | 0.916 | 0.00% |  |  |  |  |  |  |  |  |
|  |  | TC vs CC | Yes C/T | 3 | 0.870 (0.576-1.313) | 0.506 | F | 1.91 | 0.386 | 0.00% |  |  |  |  |  |  |  |  |
|  |  |  | No,T/C | 2 | 0.862 (0.433-1.716) | 0.747 | R | 3.18 | 0.075 | 68.50% |  |  |  |  |  |  |  |  |
|  |  |  | Yes,T/C | 2 | 1.982 (0.961-4.088) | 0.064 | F | 0.01 | 0.911 | 0.00% |  |  |  |  |  |  |  |  |
|  |  | TC+CC vs TT | Yes C/T | 3 | 0.992 (0.315-3.125) | 0.989 | R | 8.22 | 0.016 | 75.70% | 1.752 (0.854-3.595) | 0.126 | Sun Yongjian et al. | 9.28 | F | 0.18 | 0.673 | 0.00% |
|  |  |  | No,T/C | 2 | 0.723 (0.442-1.185) | 0.196 | F | 0.35 | 0.555 | 0.00% |  |  |  |  |  |  |  |  |
|  |  |  | Yes,T/C | 3 | 0.662 (0.465-0.943) | 0.022 | F | 1.95 | 0.377 | 0.00% |  |  |  |  |  |  |  |  |
|  |  | T vs C | Yes C/T | 3 | 1.055 (0.494-2.251) | 0.891 | R | 14.10 | 0.001 | 85.80% | 0.718 (0.515-1.002) | 0.051 | Sun Yongjian et al. | 11.68 | F | 0.38 | 0.537 | 0.00% |
|  |  |  | No,T/C | 2 | 1.122 (0.609-2.067) | 0.712 | R | 2.70 | 0.100 | 63.00% |  |  |  |  |  |  |  |  |
|  |  |  | Yes,T/C | 2 | 1.695 (1.240-2.316) | 0.001 | F | 0.03 | 0.872 | 0.00% |  |  |  |  |  |  |  |  |
|  | rs1799793 | GA vs GG | Yes | 5 | 0.956 (0.669-1.364) | 0.802 | F | 0.38 | 0.984 | 0.00% |  |  |  |  |  |  |  |  |
|  |  |  | No | 2 | 0.698 (0.395-1.235) | 0.217 | F | 0.68 | 0.409 | 0.00% |  |  |  |  |  |  |  |  |
|  |  | AA vs GG | Yes | 5 | 0.582 (0.340-0.998) | 0.049 | F | 7.29 | 0.121 | 45.10% | 0.845 (0.448-1.593) | 0.603 | Wang MJ et al. | 15.61 | F | 2.49 | 0.477 | 0.00% |
|  |  |  | No | 2 | 0.458 (0.170-1.230) | 0.121 | F | 0.01 | 0.931 | 0.00% |  |  |  |  |  |  |  |  |
|  |  | GA vs AA | Yes | 5 | 1.084 (0.619-1.896) | 0.778 | F | 3.20 | 0.525 | 0.00% |  |  |  |  |  |  |  |  |
|  |  |  | No | 2 | 1.537 (0.569-4.157) | 0.397 | F | 0.37 | 0.543 | 0.00% |  |  |  |  |  |  |  |  |
|  |  | GA+AA vs GG | Yes | 6 | 0.913 (0.690-1.208) | 0.523 | F | 1.72 | 0.887 | 0.00% |  |  |  |  |  |  |  |  |
|  |  |  | No | 2 | 0.621 (0.375-1.029) | 0.064 | F | 0.82 | 0.365 | 0.00% |  |  |  |  |  |  |  |  |
|  |  | G vs A | Yes | 5 | 1.097 (0.867-1.390) | 0.440 | F | 3.26 | 0.515 | 0.00% |  |  |  |  |  |  |  |  |
|  |  |  | No | 2 | 1.609 (1.059-2.445) | 0.026 | F | 0.44 | 0.506 | 0.00% |  |  |  |  |  |  |  |  |
|  | rs3212986 | CA vs CC | Yes | 2 | 0.844 (0.537-1.326) | 0.498 | F | 0.00 | 0.980 | 0.00% |  |  |  |  |  |  |  |  |
|  |  |  | No | 2 | 0.877 (0.601-1.281) | 0.462 | F | 0.01 | 0.930 | 0.00% |  |  |  |  |  |  |  |  |
|  |  | AA vs CC | Yes | 2 | 0.658 (0.271-1.598) | 0.355 | F | 0.03 | 0.867 | 0.00% |  |  |  |  |  |  |  |  |
|  |  |  | No | 2 | 1.089 (0.444-2.676) | 0.869 | F | 2.07 | 0.151 | 51.60% |  |  |  |  |  |  |  |  |
|  |  | CA vs AA | Yes | 2 | 1.276 (0.586-2.779) | 0.539 | F | 0.04 | 0.833 | 0.00% |  |  |  |  |  |  |  |  |
|  |  |  | No | 2 | 1.392 (0.661-2.931) | 0.383 | F | 0.07 | 0.793 | 0.00% |  |  |  |  |  |  |  |  |
|  |  | CA+AA vs CC | Yes | 3 | 0.817 (0.571-1.170) | 0.270 | F | 0.16 | 0.923 | 0.00% |  |  |  |  |  |  |  |  |
|  |  |  | No | 2 | 1.082 (0.449-2.611) | 0.860 | R | 3.21 | 0.073 | 68.80% |  |  |  |  |  |  |  |  |
|  |  | C vs A | Yes | 2 | 1.226 (0.897-1.675) | 0.201 | F | 0.09 | 0.767 | 0.00% |  |  |  |  |  |  |  |  |
|  |  |  | No | 2 | 0.989 (0.491-1.991) | 0.974 | R | 3.36 | 0.067 | 70.20% |  |  |  |  |  |  |  |  |
| Good tumor response | rs13181 | AC vs AA | Yes | 5 | 1.223 (0.884-1.693) | 0.224 | F | 1.26 | 0.869 | 0.00% |  |  |  |  |  |  |  |  |
|  |  | CC vs AA | Yes | 5 | 1.512 (0.858-2.666) | 0.153 | F | 3.93 | 0.415 | 0.00% |  |  |  |  |  |  |  |  |
|  |  | AC vs CC | Yes | 5 | 0.753 (0.437-1.297) | 0.307 | F | 1.27 | 0.867 | 0.00% |  |  |  |  |  |  |  |  |
|  |  | AC+CC vs AA | Yes | 6 | 1.300 (0.973-1.738) | 0.076 | F | 8.08 | 0.152 | 38.10% | 1.467 (1.074-2.003) | 0.016 | Sun Yongjian et al. | 10.49 | F | 3.73 | 0.444 | 0.00% |
|  |  | A vs C | Yes | 5 | 0.821 (0.656-1.028) | 0.085 | F | 5.81 | 0.214 | 31.20% | 0.744 (0.586-0.944) | 0.015 | Sun Yongjian et al. | 9.19 | F | 0.06 | 0.997 | 0.00% |
|  | rs11615 | TC vs TT | Yes,T/C | 2 | 1.543 (0.993-2.397) | 0.054 | F | 0.05 | 0.819 | 0.00% |  |  |  |  |  |  |  |  |
|  |  |  | Yes C/T | 2 | 1.103 (0.537-2.264) | 0.789 | F | 0.85 | 0.356 | 0.00% |  |  |  |  |  |  |  |  |
|  |  | CC vs TT | Yes,T/C | 2 | 2.731 (1.426-5.232) | 0.002 | F | 0.03 | 0.864 | 0.00% |  |  |  |  |  |  |  |  |
|  |  |  | Yes C/T | 2 | 1.653 (0.378-7.227) | 0.504 | R | 5.05 | 0.025 | 80.20% |  |  |  |  |  |  |  |  |
|  |  | TC vs CC | Yes,T/C | 2 | 0.458 (0.240-0.876) | 0.018 | F | 0.43 | 0.513 | 0.00% |  |  |  |  |  |  |  |  |
|  |  |  | Yes C/T | 2 | 0.727 (0.322-1.642) | 0.443 | R | 2.67 | 0.102 | 62.50% |  |  |  |  |  |  |  |  |
|  |  | TC+CC vs TT | Yes,T/C | 3 | 1.895 (1.309-2.742) | 0.001 | F | 2.47 | 0.291 | 18.90% |  |  |  |  |  |  |  |  |
|  |  |  | Yes C/T | 2 | 1.531 (0.427-5.494) | 0.513 | R | 4.05 | 0.044 | 75.30% | 2.035 (1.386-2.988) | <0.001 | Katja et al. | 5.67 | F | 0.65 | 0.418 | 0.00% |
|  |  | T vs C | Yes,T/C | 2 | 0.520 (0.389-0.695) | <0.001 | F | 0.89 | 0.344 | 0.00% |  |  |  |  |  |  |  |  |
|  |  |  | Yes C/T | 2 | 0.660 (0.227-1.921) | 0.446 | R | 9.94 | 0.002 | 89.90% |  |  |  |  |  |  |  |  |
|  | rs1799793 | GA vs GG | Yes | 5 | 1.203 (0.832-1.738) | 0.326 | F | 2.52 | 0.471 | 0.00% |  |  |  |  |  |  |  |  |
|  |  | AA vs GG | Yes | 5 | 1.298 (0.714-2.360) | 0.393 | F | 4.58 | 0.205 | 34.50% | 1.932 ( 0.930-4.012) | 0.077 | Sun Yongjian et al. | 24.81 | F | 1.14 | 0.566 | 0.00% |
|  |  | GA vs AA | Yes | 5 | 0.730 (0.401-1.330) | 0.304 | F | 1.36 | 0.715 | 0.00% |  |  |  |  |  |  |  |  |
|  |  | GA+AA vs GG | Yes | 5 | 1.231 (0.906-1.674) | 0.184 | F | 6.41 | 0.171 | 37.50% | 1.459 (1.033-2.060) | 0.032 | Sun Yongjian et al. | 16.34 | F | 1.98 | 0.576 | 0.00% |
|  |  | G vs A | Yes | 5 | 0.795 (0.517-1.224) | 0.297 | R | 8.57 | 0.036 | 65.00% | 0.659 (0.495-0.876) | 0.004 | Sun Yongjian et al. | 19.15 | F | 0.96 | 0.62 | 0.00% |
| Poor tumor response | rs13181 | AC vs AA | Yes | 5 | 1.146 (0.635-2.067) | 0.652 | R | 11.21 | 0.024 | 64.30% | 0.829 (0.586-1.172) | 0.288 | D Carolina et al. | 10.63 | F | 1.52 | 0.677 | 0.00% |
|  |  | CC vs AA | Yes | 5 | 0.893 (0.525-1.518) | 0.675 | F | 6.32 | 0.176 | 36.70% | 0.761 (0.433-1.336) | 0.341 | D Carolina et al. | 8.37 | F | 3.54 | 0.315 | 15.40% |
|  |  | AC vs CC | Yes | 5 | 1.671 (0.986-2.833) | 0.056 | F | 5.57 | 0.234 | 28.20% | 1.204 (0.659-2.201) | 0.546 | D Carolina et al. | 18.22 | F | 0.71 | 0.87 | 0.00% |
|  |  | AC+CC vs AA | Yes | 5 | 0.799 (0.588-1.085) | 0.151 | F | 4.15 | 0.386 | 3.70% | 0.704 (0.505-0.981) | 0.038 | Sun Yongjian et al. | 11.22 | F | 0.33 | 0.954 | 0.00% |
|  |  | A vs C | Yes | 5 | 1.188 (0.920-1.533) | 0.186 | F | 5.51 | 0.138 | 45.50% | 1.347 (1.022-1.775) | 0.034 | Sun Yongjian et al. | 11.02 | F | 0.05 | 0.976 | 0.00% |
|  | rs11615 | TC vs TT | Yes,T/C | 3 | 0.650 (0.440-0.959) | 0.030 | F | 2.19 | 0.335 | 8.70% | 0.591 (0.391-0.893) | 0.013 | D Carolina et al. | 6.64 | F | 0.38 | 0.539 | 0.00% |
|  |  |  | Yes C/T | 2 | 0.902 (0.440-1.848) | 0.777 | F | 0.83 | 0.362 | 0.00% |  |  |  |  |  |  |  |  |
|  |  | CC vs TT | Yes,T/C | 3 | 0.473 (0.154-1.458) | 0.193 | R | 7.22 | 0.027 | 72.30% | 0.279 (0.147-0.530) | <0.001 | D Carolina et al. | 12.49 | F | 1.04 | 0.307 | 4.10% |
|  |  |  | Yes C/T | 2 | 0.607 (0.139-2.639) | 0.505 | R | 5.11 | 0.024 | 80.40% |  |  |  |  |  |  |  |  |
|  |  | TC vs CC | Yes,T/C | 3 | 1.969 (1.129-3.431) | 0.017 | F | 0.74 | 0.690 | 0.00% |  |  |  |  |  |  |  |  |
|  |  |  | Yes C/T | 2 | 1.369 (0.605-3.094) | 0.451 | R | 2.62 | 0.106 | 61.80% |  |  |  |  |  |  |  |  |
|  |  | TC+CC vs TT | Yes,T/C | 3 | 1.615 (0.927-2.813) | 0.090 | F | 0.79 | 0.673 | 0.00% |  |  |  |  |  |  |  |  |
|  |  |  | Yes C/T | 2 | 1.071 (0.746-1.539) | 0.710 | F | 0.31 | 0.581 | 0.00% |  |  |  |  |  |  |  |  |
|  |  | T vs C | Yes,T/C | 2 | 1.939 (1.453-2.589) | <0.001 | F | 0.90 | 0.342 | 0.00% |  |  |  |  |  |  |  |  |
|  |  |  | Yes C/T | 2 | 1.515 (0.526-4.365) | 0.441 | R | 9.84 | 0.002 | 89.80% |  |  |  |  |  |  |  |  |
|  | rs1799793 | GA vs GG | Yes | 5 | 0.870 (0.630-1.202) | 0.398 | F | 3.96 | 0.411 | 0.00% |  |  |  |  |  |  |  |  |
|  |  | AA vs GG | Yes | 5 | 0.807 (0.352-1.851) | 0.612 | R | 9.59 | 0.048 | 58.30% | 0.702 (0.408-1.209) | 0.202 | D Carolina et al. | 11.45 | R | 6.20 | 0.102 | 51.60% |
|  |  | GA vs AA | Yes | 5 | 1.482 (0.886-2.480) | 0.134 | F | 1.56 | 0.815 | 0.00% |  |  |  |  |  |  |  |  |
|  |  | GA+AA vs GG | Yes | 5 | 0.811 (0.601-1.094) | 0.170 | F | 6.13 | 0.190 | 34.70% | 0.689 (0.493-0.963) | 0.029 | Sun Yongjian et al. | 15.69 | F | 1.62 | 0.656 | 0.00% |
|  |  | G vs A | Yes | 4 | 1.259 (0.822-1.929) | 0.290 | R | 8.40 | 0.038 | 64.30% | 1.520 (1.142-2.022) | 0.004 | Sun Yongjian et al. | 19.26 | F | 0.88 | 0.644 | 0.00% |
