# Supplementary material for: Meta-analysis showing that ERCC1 polymorphism is predictive of osteosarcoma prognosis
Source: Oncotarget. 2017 Jul 19;8(37):62769–79. doi: 10.18632/oncotarget.19370 (PMC5617547; doi:10.18632/oncotarget.19370)
Supplement: Supplementary file 10 [file oncotarget-08-62769-s010.doc]

Supplementary Table 9: Subgroup analysis：Confounder adjustment of tumor stage

| Index | Locus | Genetic models | Subgroups | Number of studies | Test of association | | Test of heterogeneity | | | | Test of association after sensitivity analysis | | | | Test of heterogeneity after sensitivity analysis | | | |
| --- | --- | --- | --- | --- | --- | --- | --- | --- | --- | --- | --- | --- | --- | --- | --- | --- | --- | --- |
|  |  |  |  |  | HR/OR (95%CI) | P-value | Model | Chi-square | P-value | I² | OR (95%CI) | P-value | Study removed as heterogeneity source | Percentage of removed study(%) | Model | Chi-square | P-value | I^2^ |
| OS | rs13181 | AC vs AA | Yes | 6 | 0.892 (0.655-1.214) | 0.466 | F | 0.68 | 0.984 | 0.00% |  |  |  |  |  |  |  |  |
|  |  | CC vs AA | Yes | 6 | 0.806 (0.470-1.383) | 0.434 | F | 0.43 | 0.994 | 0.00% |  |  |  |  |  |  |  |  |
|  |  | AC vs CC | Yes | 6 | 1.132 (0.685-1.871) | 0.629 | F | 0.17 | 0.999 | 0.00% |  |  |  |  |  |  |  |  |
|  |  | AC+CC vs AA | Yes | 7 | 0.871 (0.676-1.123) | 0.287 | F | 1.11 | 0.981 | 0.00% |  |  |  |  |  |  |  |  |
|  |  | A vs C | Yes | 6 | 1.146 (0.926-1.417) | 0.210 | F | 1.63 | 0.898 | 0.00% |  |  |  |  |  |  |  |  |
|  | rs11615 | TC vs TT | Yes C/T | 2 | 0.868 (0.229-3.283) | 0.835 | R | 2.73 | 0.099 | 63.30% |  |  |  |  |  |  |  |  |
|  |  |  | Yes,T/C | 3 | 0.692 (0.472-1.014) | 0.059 | F | 0.06 | 0.973 | 0.00% |  |  |  |  |  |  |  |  |
|  |  |  | No,C/T | 2 | 1.413 (0.757-2.634) | 0.277 | F | 0.00 | 0.983 | 0.00% |  |  |  |  |  |  |  |  |
|  |  | CC vs TT | Yes C/T | 2 | 0.857 (0.103-7.108) | 0.886 | R | 8.32 | 0.004 | 88.00% |  |  |  |  |  |  |  |  |
|  |  |  | Yes,T/C | 3 | 0.376 (0.205-0.688) | 0.002 | F | 0.16 | 0.923 | 0.00% |  |  |  |  |  |  |  |  |
|  |  |  | No,C/T | 2 | 1.899 (1.028-3.506) | 0.041 | F | 0.12 | 0.725 | 0.00% |  |  |  |  |  |  |  |  |
|  |  | TC vs CC | Yes C/T | 2 | 0.886 (0.508-1.546) | 0.670 | F | 1.90 | 0.168 | 47.30% |  |  |  |  |  |  |  |  |
|  |  |  | Yes,T/C | 3 | 1.781 (1.009-3.143) | 0.046 | F | 0.23 | 0.891 | 0.00% |  |  |  |  |  |  |  |  |
|  |  |  | No,C/T | 2 | 0.753 (0.506-1.119) | 0.161 | F | 0.26 | 0.611 | 0.00% |  |  |  |  |  |  |  |  |
|  |  | TC+CC vs TT | Yes C/T | 2 | 0.809 (0.133-4.933) | 0.818 | R | 6.53 | 0.011 | 84.70% |  |  |  |  |  |  |  |  |
|  |  |  | Yes,T/C | 4 | 0.662 (0.489-0.895) | 0.007 | F | 1.95 | 0.582 | 0.00% |  |  |  |  |  |  |  |  |
|  |  |  | No,C/T | 2 | 1.652 (0.919-2.969) | 0.093 | F | 0.04 | 0.842 | 0.00% |  |  |  |  |  |  |  |  |
|  |  | T vs C | Yes C/T | 2 | 1.231 (0.338-4.477) | 0.753 | R | 12.37 | <0.001 | 91.90% |  |  |  |  |  |  |  |  |
|  |  |  | Yes,T/C | 3 | 1.619 (1.256-2.087) | <0.001 | F | 0.27 | 0.874 | 0.00% |  |  |  |  |  |  |  |  |
|  |  |  | No,C/T | 2 | 0.722 (0.543-0.960) | 0.025 | F | 0.26 | 0.613 | 0.00% |  |  |  |  |  |  |  |  |
|  | rs1799793 | GA vs GG | Yes | 6 | 0.928 (0.676-1.276) | 0.647 | F | 0.50 | 0.992 | 0.00% |  |  |  |  |  |  |  |  |
|  |  | AA vs GG | Yes | 6 | 0.560 (0.340-0.923) | 0.023 | F | 7.43 | 0.190 | 32.70% | 0.750 (0.423-1.330) | 0.325 | M.J.Wang et al. | 11.69 | F | 3.23 | 0.52 | 0.00% |
|  |  | GA vs AA | Yes | 6 | 1.197 (0.721-1.988) | 0.488 | F | 3.89 | 0.566 | 0.00% |  |  |  |  |  |  |  |  |
|  |  | GA+AA vs GG | Yes | 7 | 0.875 (0.679-1.127) | 0.300 | F | 2.21 | 0.899 | 0.00% |  |  |  |  |  |  |  |  |
|  |  | G vs A | Yes | 6 | 1.158 (0.936-1.434) | 0.177 | F | 4.35 | 0.500 | 0.00% |  |  |  |  |  |  |  |  |
|  | rs3212986 | CA vs CC | Yes | 2 | 0.844 (0.537-1.326) | 0.462 | F | 0.00 | 0.980 | 0.00% |  |  |  |  |  |  |  |  |
|  |  |  | No | 2 | 0.877 (0.601-1.281) | 0.498 | F | 0.01 | 0.930 | 0.00% |  |  |  |  |  |  |  |  |
|  |  | AA vs CC | Yes | 2 | 0.658 (0.271-1.598) | 0.355 | F | 0.03 | 0.867 | 0.00% |  |  |  |  |  |  |  |  |
|  |  |  | No | 2 | 1.089 (0.444-2.676) | 0.852 | R | 2.07 | 0.151 | 51.60% |  |  |  |  |  |  |  |  |
|  |  | CA vs AA | Yes | 2 | 1.276 (0.586-2.779) | 0.539 | F | 0.04 | 0.833 | 0.00% |  |  |  |  |  |  |  |  |
|  |  |  | No | 2 | 1.392 (0.661-2.931) | 0.383 | F | 0.07 | 0.793 | 0.00% |  |  |  |  |  |  |  |  |
|  |  | CA+AA vs CC | Yes | 3 | 0.817 (0.571-1.170) | 0.270 | F | 0.16 | 0.923 | 0.00% |  |  |  |  |  |  |  |  |
|  |  |  | No | 2 | 0.905 (0.388-2.112) | 0.860 | R | 3.21 | 0.073 | 68.80% |  |  |  |  |  |  |  |  |
|  |  | C vs A | Yes | 2 | 1.226 (0.897-1.675) | 0.201 | F | 0.09 | 0.767 | 0.00% |  |  |  |  |  |  |  |  |
|  |  |  | No | 2 | 0.989 (0.491-1.991) | 0.974 | R | 3.36 | 0.067 | 70.20% |  |  |  |  |  |  |  |  |
| Good tumor response | rs13181 | AC vs AA | Yes | 5 | 1.166 (0.841-1.615) | 0.356 | F | 1.20 | 0.879 | 0.00% |  |  |  |  |  |  |  |  |
|  |  | CC vs AA | Yes | 5 | 1.253 (0.726-2.161) | 0.418 | F | 2.59 | 0.628 | 0.00% |  |  |  |  |  |  |  |  |
|  |  | AC vs CC | Yes | 5 | 0.852 (0.509-1.426) | 0.542 | F | 0.74 | 0.946 | 0.00% |  |  |  |  |  |  |  |  |
|  |  | AC+CC vs AA | Yes | 6 | 1.268 (0.950-1.693) | 0.107 | F | 8.26 | 0.142 | 39.50% | 1.423 (1.044-1.940) | 0.025 | Sun Yongjian et al. | 10.49 | F | 4.19 | 0.381 | 4.50% |
|  |  | A vs C | Yes | 5 | 0.851 (0.682-1.062) | 0.154 | F | 5.73 | 0.220 | 30.20% | 0.777 (0.614-0.983) | 0.035 | Sun Yongjian et al. | 9.19 | F | 0.53 | 0.913 | 0.00% |
|  | rs11615 | TC vs TT | Yes,T/C | 3 | 1.486 (1.032-2.138) | 0.033 | F | 0.14 | 0.932 | 0.00% |  |  |  |  |  |  |  |  |
|  |  | CC vs TT | Yes,T/C | 3 | 2.659 (1.554-4.548) | <0.001 | F | 0.05 | 0.975 | 0.00% |  |  |  |  |  |  |  |  |
|  |  | TC vs CC | Yes,T/C | 3 | 0.498 (0.296-0.839) | 0.009 | F | 0.61 | 0.739 | 0.00% |  |  |  |  |  |  |  |  |
|  |  | TC+CC vs TT | Yes,T/C | 4 | 1.800 (1.322-2.450) | <0.001 | F | 2.71 | 0.439 | 0.00% |  |  |  |  |  |  |  |  |
|  |  | T vs C | Yes,T/C | 3 | 0.554 (0.437-0.702) | <0.001 | F | 1.45 | 0.484 | 0.00% |  |  |  |  |  |  |  |  |
| Poor tumor response | rs13181 | AC vs AA | Yes | 5 | 0.847 (0.626-1.146) | 0.283 | F | 1.59 | 0.811 | 0.00% |  |  |  |  |  |  |  |  |
|  |  | CC vs AA | Yes | 5 | 0.779 (0.481-1.260) | 0.309 | F | 3.57 | 0.467 | 0.00% |  |  |  |  |  |  |  |  |
|  |  | AC vs CC | Yes | 5 | 1.175 (0.702-1.967) | 0.540 | F | 0.74 | 0.947 | 0.00% |  |  |  |  |  |  |  |  |
|  |  | AC+CC vs AA | Yes | 6 | 0.818 (0.625-1.072) | 0.146 | F | 4.26 | 0.512 | 0.00% |  |  |  |  |  |  |  |  |
|  |  | A vs C | Yes | 5 | 1.171 (0.938-1.461) | 0.163 | F | 5.56 | 0.235 | 28.00% | 1.280 (1.012-1.619) | 0.039 | Sun Yongjian et al. | 9.05 | F | 0.52 | 0.914 | 0.00% |
|  | rs11615 | TC vs TT | Yes,T/C | 3 | 0.632 (0.450-0.889) | 0.008 | F | 0.70 | 0.704 | 0.00% |  |  |  |  |  |  |  |  |
|  |  | CC vs TT | Yes,T/C | 3 | 0.323 (0.193-0.540) | <0.001 | F | 1.60 | 0.449 | 0.00% |  |  |  |  |  |  |  |  |
|  |  | TC vs CC | Yes,T/C | 3 | 2.000 (1.188-3.368) | 0.009 | F | 0.58 | 0.747 | 0.00% |  |  |  |  |  |  |  |  |
|  |  | TC+CC vs TT | Yes,T/C | 4 | 1.573 (0.990-2.500) | 0.055 | F | 0.82 | 0.845 | 0.00% |  |  |  |  |  |  |  |  |
|  |  | T vs C | Yes,T/C | 3 | 1.814 (1.431-2.300) | <0.001 | F | 1.53 | 0.464 | 0.00% |  |  |  |  |  |  |  |  |
