# Supplementary material for: Meta-analysis showing that ERCC1 polymorphism is predictive of osteosarcoma prognosis
Source: Oncotarget. 2017 Jul 19;8(37):62769–79. doi: 10.18632/oncotarget.19370 (PMC5617547; doi:10.18632/oncotarget.19370)
Supplement: Supplementary file 11 [file oncotarget-08-62769-s011.doc]

Supplementary Table 10: Subgroup analysis：HWE

| Index | Locus | Genetic models | Subgroups | Number of studies | Test of association | | Test of heterogeneity | | | | Test of association after sensitivity analysis | | | | Test of heterogeneity after sensitivity analysis | | | |
| --- | --- | --- | --- | --- | --- | --- | --- | --- | --- | --- | --- | --- | --- | --- | --- | --- | --- | --- |
|  |  |  |  |  | HR/OR (95%CI) | P-value | Model | Chi-square | P-value | I² | OR (95%CI) | P-value | Study removed as heterogeneity source | Percentage of removed study(%) | Model | Chi-square | P-value | I^2^ |
| OS | rs13181 | AC vs AA | Yes | 4 | 0.835 (0.583-1.195) | 0.324 | F | 0.490 | 0.922 | 0.00% |  |  |  |  |  |  |  |  |
|  |  |  | No | 3 | 0.932 (0.578-1.505) | 0.774 | F | 0.280 | 0.870 | 0.00% |  |  |  |  |  |  |  |  |
|  |  | CC vs AA | Yes | 4 | 0.651 (0.327-1.293) | 0.220 | F | 0.560 | 0.905 | 0.00% |  |  |  |  |  |  |  |  |
|  |  |  | No | 3 | 0.881 (0.414-1.877) | 0.744 | F | 0.220 | 0.898 | 0.00% |  |  |  |  |  |  |  |  |
|  |  | AC vs CC | Yes | 4 | 1.276 (0.670-2.431) | 0.458 | F | 0.420 | 0.936 | 0.00% |  |  |  |  |  |  |  |  |
|  |  |  | No | 3 | 1.084 (0.529-2.221) | 0.146 | F | 0.010 | 0.995 | 0.00% |  |  |  |  |  |  |  |  |
|  |  | AC+CC vs AA | Yes | 4 | 0.819 (0.583-1.151) | 0.250 | F | 0.490 | 0.921 | 0.00% |  |  |  |  |  |  |  |  |
|  |  |  | No | 3 | 0.926 (0.626-1.371) | 0.703 | F | 0.590 | 0.746 | 0.00% |  |  |  |  |  |  |  |  |
|  |  | A vs C | Yes | 4 | 1.191 (0.916-1.548) | 0.192 | F | 0.650 | 0.884 | 0.00% |  |  |  |  |  |  |  |  |
|  |  |  | No | 3 | 1.139 (0.830-1.563) | 0.420 | F | 1.290 | 0.524 | 0.00% |  |  |  |  |  |  |  |  |
|  | rs11615 | TC vs TT | Yes,C/T | 3 | 1.476 (0.855-2.550) | 0.162 | F | 0.080 | 0.959 | 0.00% |  |  |  |  |  |  |  |  |
|  |  |  | Yes,T/C | 3 | 0.679 (0.450-1.026) | 0.066 | F | 0.010 | 0.993 | 0.00% |  |  |  |  |  |  |  |  |
|  |  | CC vs TT | Yes,C/T | 3 | 2.035 (1.184-3.497) | 0.010 | F | 0.350 | 0.841 | 0.00% |  |  |  |  |  |  |  |  |
|  |  |  | Yes,T/C | 3 | 0.558 (0.217-1.435) | 0.226 | R | 4.550 | 0.103 | 56.10% | 0.338 (0.151-0.758) | 0.008 | Paola Biason et al. | 12.91 | F | 0.01 | 0.916 | 0.00% |
|  |  | TC vs CC | Yes,C/T | 3 | 0.729 (0.516-1.029) | 0.072 | F | 0.360 | 0.835 | 0.00% |  |  |  |  |  |  |  |  |
|  |  |  | Yes,T/C | 3 | 1.187 (0.428-3.292) | 0.742 | R | 5.740 | 0.057 | 65.20% | 1.982 (0.961-4.088) | 0.064 | Paola Biason et al. | 8.65 | F | 0.01 | 0.911 | 0.00% |
|  |  | TC+CC vs TT | Yes,C/T | 3 | 1.741 (1.039-2.918) | 0.035 | F | 0.180 | 0.919 | 0.00% |  |  |  |  |  |  |  |  |
|  |  |  | Yes,T/C | 3 | 0.621 (0.429-0.899) | 0.012 | F | 0.810 | 0.666 | 0.00% |  |  |  |  |  |  |  |  |
|  |  | T vs C | Yes,C/T | 3 | 0.701 (0.547-0.897) | 0.005 | F | 0.430 | 0.808 | 0.00% |  |  |  |  |  |  |  |  |
|  |  |  | Yes,T/C | 3 | 1.445 (1.095-1.908) | 0.009 | F | 4.870 | 0.088 | 58.90% | 1.695 (1.240-2.316) | 0.001 | Paola Biason et al. | 8.84 | F | 0.03 | 0.872 | 0.00% |
|  | rs1799793 | GA vs GG | Yes | 3 | 0.926 (0.605-1.415) | 0.721 | F | 0.030 | 0.984 | 0.00% |  |  |  |  |  |  |  |  |
|  |  |  | No | 4 | 0.826 (0.537-1.270) | 0.383 | F | 1.730 | 0.631 | 0.00% |  |  |  |  |  |  |  |  |
|  |  | AA vs GG | Yes | 3 | 0.358 (0.171-0.749) | 0.006 | F | 3.220 | 0.200 | 38.00% | 0.222 (0.090-0.548) | 0.001 | Zhang Q et al. | 13.75 | F | 0 | 0.698 | 0.00% |
|  |  |  | No | 4 | 0.745 (0.402-1.380) | 0.349 | F | 2.020 | 0.568 | 0.00% |  |  |  |  |  |  |  |  |
|  |  | GA vs AA | Yes | 3 | 1.133 (0.527-2.437) | 0.749 | F | 3.110 | 0.212 | 35.60% | 0.798 (0.334-1.908) | 0.612 | Liu ZF et al. | 9.22 | F | 0.38 | 0.538 | 0.00% |
|  |  |  | No | 4 | 1.211 (0.643-2.278) | 0.554 | F | 0.810 | 0.848 | 0.00% |  |  |  |  |  |  |  |  |
|  |  | GA+AA vs GG | Yes | 3 | 0.847 (0.582-1.231) | 0.383 | F | 0.500 | 0.780 | 0.00% |  |  |  |  |  |  |  |  |
|  |  |  | No | 4 | 0.795 (0.557-1.133) | 0.204 | F | 3.490 | 0.322 | 14.10% | 0.687 (0.460-1.028) | 0.068 | Sun Yongjian et al. | 9.10 | F | 1.24 | 0.547 | 0.00% |
|  |  | G vs A | Yes | 3 | 1.166 (0.865-1.570) | 0.313 | F | 1.550 | 0.460 | 0.00% |  |  |  |  |  |  |  |  |
|  |  |  | No | 4 | 1.240 (0.933-1.647) | 0.138 | F | 4.510 | 0.212 | 33.40% | 1.438 (1.039-1.991) | 0.029 | Sun Yongjian et al. | 9.43 | F | 1.15 | 0.564 | 0.00% |
| Good tumor response | rs13181 | AC vs AA | Yes | 3 | 1.323 (0.900-1.946) | 0.155 | F | 0.080 | 0.960 | 0.00% |  |  |  |  |  |  |  |  |
|  |  |  | No | 3 | 1.050 (0.672-1.640) | 0.832 | F | 0.660 | 0.719 | 0.00% |  |  |  |  |  |  |  |  |
|  |  | CC vs AA | Yes | 3 | 2.058 (0.977-4.337) | 0.058 | F | 0.620 | 0.733 | 0.00% |  |  |  |  |  |  |  |  |
|  |  |  | No | 3 | 1.076 (0.550-2.102) | 0.831 | F | 1.840 | 0.399 | 0.00% |  |  |  |  |  |  |  |  |
|  |  | AC vs CC | Yes | 3 | 0.677 (0.351-1.307) | 0.245 | F | 0.350 | 0.840 | 0.00% |  |  |  |  |  |  |  |  |
|  |  |  | No | 3 | 0.929 (0.466-1.854) | 0.834 | F | 0.600 | 0.739 | 0.00% |  |  |  |  |  |  |  |  |
|  |  | AC+CC vs AA | Yes | 3 | 1.375 (0.973-1.944) | 0.071 | F | 0.190 | 0.907 | 0.00% |  |  |  |  |  |  |  |  |
|  |  |  | No | 3 | 1.022 (0.686-1.521) | 0.917 | F | 2.670 | 0.264 | 25.00% | 1.022 (0.686-1.521) | 0.385 | Sun Yongjian et al. | 10.75 | F | 0.24 | 0.625 | 0.00% |
|  |  | A vs C | Yes | 3 | 0.747 (0.575-0.970) | 0.029 | F | 0.050 | 0.975 | 0.00% |  |  |  |  |  |  |  |  |
|  |  |  | No | 3 | 1.009 (0.634-1.607) | 0.968 | R | 4.180 | 0.124 | 52.20% | 0.826 (0.579-1.177) | 0.290 | Sun Yongjian et al. | 10.00 | F | 0.28 | 0.595 | 0.00% |
|  | rs11615 | TC vs TT | Yes,T/C | 2 | 1.543 (0.993-2.397) | 0.054 | F | 0.050 | 0.819 | 0.00% |  |  |  |  |  |  |  |  |
|  |  | CC vs TT | Yes,T/C | 2 | 2.731 (1.426-5.232) | 0.002 | F | 0.030 | 0.864 | 0.00% |  |  |  |  |  |  |  |  |
|  |  | TC vs CC | Yes,T/C | 2 | 0.458 (0.240-0.876) | 0.018 | F | 0.430 | 0.513 | 0.00% |  |  |  |  |  |  |  |  |
|  |  | TC+CC vs TT | Yes,T/C | 2 | 2.035 (1.386-2.988) | <0.001 | F | 0.650 | 0.418 | 0.00% |  |  |  |  |  |  |  |  |
|  |  | T vs C | Yes,T/C | 2 | 0.520 (0.389-0.695) | <0.001 | F | 0.890 | 0.344 | 0.00% |  |  |  |  |  |  |  |  |
|  | rs1799793 | GA vs GG | Yes | 2 | 1.393 (0.859-2.261) | 0.179 | F | 0.740 | 0.389 | 0.00% |  |  |  |  |  |  |  |  |
|  |  |  | No | 3 | 1.140 (0.735-1.767) | 0.599 | F | 1.590 | 0.451 | 0.00% |  |  |  |  |  |  |  |  |
|  |  | AA vs GG | Yes | 2 | 2.161 (0.803-5.819) | 0.127 | F | 1.030 | 0.310 | 3.00% |  |  |  |  |  |  |  |  |
|  |  |  | No | 3 | 1.282 (0.698-2.354) | 0.423 | F | 3.510 | 0.173 | 43.00% | 1.282 (0.698-2.354) | 0.084 | Sun Yongjian et al. | 24.81 | F | 0.11 | 0.735 | 0.00% |
|  |  | GA vs AA | Yes | 2 | 0.501 (0.191-1.317) | 0.161 | F | 0.040 | 0.842 | 0.00% |  |  |  |  |  |  |  |  |
|  |  |  | No | 3 | 0.812 (0.442-1.492) | 0.503 | F | 0.670 | 0.716 | 0.00% |  |  |  |  |  |  |  |  |
|  |  | GA+AA vs GG | Yes | 2 | 1.617 (1.042-2.511) | 0.032 | F | 0.860 | 0.354 | 0.00% |  |  |  |  |  |  |  |  |
|  |  |  | No | 3 | 1.152 (0.679-1.956) | 0.600 | R | 4.420 | 0.110 | 54.70% | 1.491 (0.982-2.266) | 0.061 | Sun Yongjian et al. | 18.50 | F | 0.17 | 0.682 | 0.00% |
|  |  | G vs A | Yes | 2 | 0.623 (0.437-0.888) | 0.009 | F | 0.690 | 0.407 | 0.00% |  |  |  |  |  |  |  |  |
|  |  |  | No | 3 | 0.869 (0.509-1.481) | 0.605 | R | 7.200 | 0.027 | 72.20% | 0.664 (0.477-0.925) | 0.015 | Sun Yongjian et al. | 19.15 | F | 0.28 | 0.596 | 0.00% |
| Poor | rs13181 | AC vs AA | Yes | 3 | 0.964 (0.654-1.421) | 0.853 | R | 10.260 | 0.006 | 80.50% | 0.769 (0.509-1.161) | 0.211 | D Carolina et al. | 6.51 | F | 0.15 | 0.700 | 0.00% |
|  |  |  | No | 3 | 0.949 (0.609-1.480) | 0.818 | F | 0.970 | 0.615 | 0.00% |  |  |  |  |  |  |  |  |
|  |  | CC vs AA | Yes | 3 | 0.803 (0.413-1.561) | 0.571 | F | 3.550 | 0.169 | 43.70% | 0.599 (0.288-1.246) | 0.170 | D Carolina et al. | 8.37 | F | 0.04 | 0.841 | 0.00% |
|  |  |  | No | 3 | 0.951 (0.502-1.801) | 0.877 | F | 2.660 | 0.265 | 24.80% | 0.705 (0.334-1.486) | 0.358 | Sun Yongjian et al. | 12.20 | F | 0.34 | 0.558 | 0.00% |
|  |  | AC vs CC | Yes | 4 | 2.039 (1.085-3.829) | 0.027 | F | 3.710 | 0.157 | 46.00% | 1.315 (0.608-2.847) | 0.487 | D Carolina et al. | 18.22 | F | 0 | 0.992 | 0.00% |
|  |  |  | No | 3 | 1.073 (0.538-2.143) | 0.841 | F | 0.590 | 0.745 | 0.00% |  |  |  |  |  |  |  |  |
|  |  | AC+CC vs AA | Yes | 2 | 0.725 (0.494-1.065) | 0.101 | F | 0.090 | 0.758 | 0.00% |  |  |  |  |  |  |  |  |
|  |  |  | No | 3 | 0.978 (0.656-1.459) | 0.913 | F | 2.530 | 0.282 | 21.00% | 0.819 (0.517-1.299) | 0.397 | Sun Yongjian et al. | 9.61 | F | 0.24 | 0.628 | 0.00% |
|  |  | A vs C | Yes | 2 | 1.340 (0.980-1.834) | 0.067 | F | 0.040 | 0.835 | 0.00% |  |  |  |  |  |  |  |  |
|  |  |  | No | 3 | 0.992 (0.626-1.572) | 0.972 | R | 4.090 | 0.130 | 51.10% | 1.207 (0.847-1.720) | 0.297 | Sun Yongjian et al. | 9.74 | F | 0.29 | 0.590 | 0.00% |
|  | rs11615 | TC vs TT | Yes,T/C | 3 | 0.650 (0.440-0.959) | 0.030 | F | 2.190 | 0.335 | 8.70% | 0.591 (0.391-0.893) | 0.013 | D Carolina et al. | 6.64 | F | 0.38 | 0.539 | 0.00% |
|  |  | CC vs TT | Yes,T/C | 3 | 0.473 (0.154-1.458) | 0.193 | R | 7.220 | 0.027 | 72.30% | 0.279 (0.147-0.530) | <0.001 | D Carolina et al. | 12.49 | F | 1.04 | 0.307 | 4.10% |
|  |  | TC vs CC | Yes,T/C | 3 | 1.969 (1.129-3.431) | 0.017 | F | 0.740 | 0.690 | 0.00% |  |  |  |  |  |  |  |  |
|  |  | TC+CC vs TT | Yes,T/C | 2 | 1.799 (0.955-3.389) | 0.069 | F | 0.310 | 0.577 | 0.00% |  |  |  |  |  |  |  |  |
|  |  | T vs C | Yes,T/C | 2 | 1.939 (1.453-2.589) | <0.001 | F | 0.900 | 0.342 | 0.00% |  |  |  |  |  |  |  |  |
|  | rs1799793 | GA vs GG | Yes | 3 | 0.788 (0.522-1.190) | 0.257 | F | 2.200 | 0.333 | 9.10% | 0.697 (0.445-1.091) | 0.114 | D Carolina et al. | 7.77 | F | 0.36 | 0.547 | 0.00% |
|  |  |  | No | 3 | 0.878 (0.586-1.316) | 0.530 | F | 1.980 | 0.372 | 0.00% |  |  |  |  |  |  |  |  |
|  |  | AA vs GG | Yes | 3 | 0.672 (0.154-2.937) | 0.597 | R | 6.250 | 0.044 | 68.00% | 0.340 (0.133-0.867) | 0.024 | D Carolina et al. | 11.45 | F | 0.29 | 0.593 | 0.00% |
|  |  |  | No | 3 | 0.774 (0.350-1.711) | 0.527 | R | 4.280 | 0.118 | 53.30% | 0.519 (0.267-1.009) | 0.053 | Sun Yongjian et al. | 20.08 | F | 0.13 | 0.714 | 0.00% |
|  |  | GA vs AA | Yes | 3 | 1.903 (0.954-3.799) | 0.068 | F | 0.060 | 0.969 | 0.00% |  |  |  |  |  |  |  |  |
|  |  |  | No | 3 | 1.235 (0.669-2.280) | 0.500 | F | 0.660 | 0.718 | 0.00% |  |  |  |  |  |  |  |  |
|  |  | GA+AA vs GG | Yes | 2 | 0.625 (0.407-0.960) | 0.032 | F | 0.670 | 0.412 | 0.00% |  |  |  |  |  |  |  |  |
|  |  |  | No | 3 | 0.868 (0.510-1.478) | 0.602 | R | 4.530 | 0.104 | 55.80% | 0.669 (0.442-1.013) | 0.057 | Sun Yongjian et al. | 18.05 | F | 0.15 | 0.700 | 0.00% |
|  |  | G vs A | Yes | 2 | 1.601 (1.123-2.283) | 0.009 | F | 0.640 | 0.423 | 0.00% |  |  |  |  |  |  |  |  |
|  |  |  | No | 3 | 1.154 (0.681-1.958) | 0.594 | R | 7.050 | 0.030 | 71.60% | 1.506 (1.080-2.100) | 0.016 | Sun Yongjian et al. | 19.26 | F | 0.24 | 0.624 | 0.00% |
