# Supplementary material for: Meta-analysis showing that ERCC1 polymorphism is predictive of osteosarcoma prognosis
Source: Oncotarget. 2017 Jul 19;8(37):62769–79. doi: 10.18632/oncotarget.19370 (PMC5617547; doi:10.18632/oncotarget.19370)
Supplement: Supplementary file 12 [file oncotarget-08-62769-s012.doc]

Supplementary Table 11: Subgroup analysis：Metastasis at diagnosis

| Index | Locus | Genetic models | Subgroups | Number of studies | Test (of (association | | Test of heterogeneity | | | | Test of association after sensitivity analysis | | | | Test of heterogeneity after sensitivity analysis | | | |
| --- | --- | --- | --- | --- | --- | --- | --- | --- | --- | --- | --- | --- | --- | --- | --- | --- | --- | --- |
|  |  |  |  |  | HR/OR (95%CI) | P-value | Model | Chi-square | P-value | I² | OR (95%CI) | P-value | Study removed as heterogeneity source | Percentage of removed study(%) | Model | Chi-square | P-value | I^2^ |
| OS | rs13181 | AC vs AA | No | 4 | 0.819 (0.550-1.218) | 0.323 | F | 0.70 | 0.873 | 0.00% |  |  |  |  |  |  |  |  |
|  |  |  | NA | 3 | 0.927 (0.611-1.406) | 0.721 | F | 0.02 | 0.992 | 0.00% |  |  |  |  |  |  |  |  |
|  |  | CC vs AA | No | 4 | 0.727 (0.374-1.413) | 0.347 | F | 1.03 | 0.795 | 0.00% |  |  |  |  |  |  |  |  |
|  |  |  | NA | 3 | 0.774 (0.351-1.706) | 0.525 | F | 0.08 | 0.962 | 0.00% |  |  |  |  |  |  |  |  |
|  |  | AC vs CC | No | 4 | 1.184 (0.610-2.295) | 0.618 | F | 0.42 | 0.936 | 0.00% |  |  |  |  |  |  |  |  |
|  |  |  | NA | 3 | 1.190 (0.594-2.383) | 0.623 | F | 0.12 | 0.942 | 0.00% |  |  |  |  |  |  |  |  |
|  |  | AC+CC vs AA | No | 4 | 0.838 (0.586-1.199) | 0.334 | F | 1.20 | 0.752 | 0.00% |  |  |  |  |  |  |  |  |
|  |  |  | NA | 4 | 0.871 (0.625-1.214) | 0.415 | F | 0.11 | 0.990 | 0.00% |  |  |  |  |  |  |  |  |
|  |  | A vs C | No | 4 | 1.170 (0.881-1.553) | 0.278 | F | 1.46 | 0.690 | 0.00% |  |  |  |  |  |  |  |  |
|  |  |  | NA | 3 | 1.169 (0.877-1.558) | 0.288 | F | 0.52 | 0.770 | 0.00% |  |  |  |  |  |  |  |  |
|  | rs11615 | TC vs TT | No,C/T | 2 | 0.868 (0.229-3.283) | 0.835 | R | 2.73 | 0.099 | 63.30% |  |  |  |  |  |  |  |  |
|  |  |  | No,T/C | 2 | 0.729 (0.418-1.273) | 0.267 | F | 0.00 | 0.945 | 0.00% |  |  |  |  |  |  |  |  |
|  |  |  | NA,T/C | 2 | 0.672 (0.425-1.063) | 0.090 | F | 0.00 | 0.950 | 0.00% |  |  |  |  |  |  |  |  |
|  |  |  | Yes,C/T | 2 | 1.413 (0.757-2.634) | 0.277 | F | 0.00 | 0.983 | 0.00% |  |  |  |  |  |  |  |  |
|  |  | CC vs TT | No,C/T | 2 | 0.857 (0.103-7.108) | 0.886 | R | 8.32 | 0.004 | 88.00% |  |  |  |  |  |  |  |  |
|  |  |  | No,T/C | 2 | 0.745 (0.250-2.218) | 0.597 | R | 2.75 | 0.097 | 63.70% |  |  |  |  |  |  |  |  |
|  |  |  | NA,T/C | 2 | 0.338 (0.151-0.758) | 0.008 | F | 0.01 | 0.916 | 0.00% |  |  |  |  |  |  |  |  |
|  |  |  | Yes,C/T | 2 | 1.899 (1.028-3.506) | 0.041 | F | 0.12 | 0.725 | 0.00% |  |  |  |  |  |  |  |  |
|  |  | TC vs CC | No,C/T | 2 | 0.886 (0.508-1.546) | 0.670 | F | 1.90 | 0.168 | 47.30% |  |  |  |  |  |  |  |  |
|  |  |  | No,T/C | 2 | 0.814 (0.234-2.832) | 0.747 | R | 3.23 | 0.072 | 69.00% |  |  |  |  |  |  |  |  |
|  |  |  | NA,T/C | 2 | 1.982 (0.961-4.088) | 0.064 | F | 0.01 | 0.911 | 0.00% |  |  |  |  |  |  |  |  |
|  |  |  | Yes,C/T | 2 | 0.753 (0.506-1.119) | 0.161 | F | 0.26 | 0.611 | 0.00% |  |  |  |  |  |  |  |  |
|  |  | TC+CC vs TT | No,C/T | 2 | 0.809 (0.133-4.933) | 0.818 | R | 6.53 | 0.011 | 84.70% |  |  |  |  |  |  |  |  |
|  |  |  | No,T/C | 2 | 0.723 (0.442-1.185) | 0.198 | F | 0.35 | 0.555 | 0.00% |  |  |  |  |  |  |  |  |
|  |  |  | NA,T/C | 3 | 0.662 (0.465-0.943) | 0.022 | F | 1.95 | 0.377 | 0.00% |  |  |  |  |  |  |  |  |
|  |  |  | Yes,C/T | 2 | 1.652 (0.919-2.969) | 0.093 | F | 0.04 | 0.842 | 0.00% |  |  |  |  |  |  |  |  |
|  |  | T vs C | No,C/T | 2 | 0.722 (0.543-0.960) | 0.753 | R | 12.37 | <0.001 | 91.90% |  |  |  |  |  |  |  |  |
|  |  |  | No,T/C | 2 | 1.122 (0.609-2.067) | 0.712 | R | 2.70 | 0.100 | 63.00% |  |  |  |  |  |  |  |  |
|  |  |  | NA,T/C | 2 | 1.695 (1.240-2.316) | 0.001 | F | 0.03 | 0.872 | 0.00% |  |  |  |  |  |  |  |  |
|  |  |  | Yes,C/T | 2 | 0.722 (0.543-0.960) | 0.025 | F | 0.26 | 0.613 | 0.00% |  |  |  |  |  |  |  |  |
|  | rs1799793 | GA vs GG | No | 4 | 0.846 (0.553-1.296) | 0.443 | F | 1.83 | 0.608 | 0.00% |  |  |  |  |  |  |  |  |
|  |  |  | NA | 3 | 0.905 (0.590-1.389) | 0.648 | F | 0.02 | 0.991 | 0.00% |  |  |  |  |  |  |  |  |
|  |  | AA vs GG | No | 4 | 0.474 (0.258-0.872) | 0.016 | F | 5.35 | 0.148 | 43.90% | 0.322 (0.158-0.655) | 0.002 | Sun Yongjian et al. | 11.69 | F | 1.03 | 0.599 | 0.00% |
|  |  |  | NA | 3 | 0.692 (0.327-1.465) | 0.336 | F | 1.54 | 0.464 | 0.00% |  |  |  |  |  |  |  |  |
|  |  | GA vs AA | No | 4 | 1.032 (0.527-2.022) | 0.926 | F | 1.74 | 0.628 | 0.00% |  |  |  |  |  |  |  |  |
|  |  |  | NA | 3 | 1.365 (0.673-2.769) | 0.389 | F | 1.88 | 0.391 | 0.00% |  |  |  |  |  |  |  |  |
|  |  | GA+AA vs GG | No | 4 | 0.805 (0.564-1.149) | 0.232 | F | 3.53 | 0.317 | 15.30% | 0.698 (0.466-1.045) | 0.081 | Sun Yongjian et al. | 8.41 | F | 1.39 | 0.498 | 0.00% |
|  |  |  | NA | 4 | 0.861 (0.614-1.205) | 0.383 | F | 0.64 | 0.886 | 0.00% |  |  |  |  |  |  |  |  |
|  |  | G vs A | No | 4 | 1.183 (0.886-1.580) | 0.253 | F | 4.88 | 0.180 | 38.60% | 1.361 (0.976-1.899) | 0.069 | Sun Yongjian et al. | 9.43 | F | 2.12 | 0.347 | 5.50% |
|  |  |  | NA | 3 | 1.225 (0.914-1.642) | 0.174 | F | 1.23 | 0.540 | 0.00% |  |  |  |  |  |  |  |  |
|  | rs3212986 | CA vs CC | NA | 2 | 0.844 (0.537-1.326) | 0.462 | F | 0.00 | 0.980 | 0.00% |  |  |  |  |  |  |  |  |
|  |  | AA vs CC | NA | 2 | 0.658 (0.271-1.598) | 0.355 | F | 0.03 | 0.867 | 0.00% |  |  |  |  |  |  |  |  |
|  |  | CA vs AA | NA | 2 | 1.276 (0.586-2.779) | 0.539 | F | 0.04 | 0.833 | 0.00% |  |  |  |  |  |  |  |  |
|  |  | CA+AA vs CC | NA | 3 | 0.817 (0.571-1.170) | 0.270 | F | 0.16 | 0.923 | 0.00% |  |  |  |  |  |  |  |  |
|  |  | C vs A | NA | 2 | 1.226 (0.897-1.675) | 0.201 | F | 0.09 | 0.767 | 0.00% |  |  |  |  |  |  |  |  |
| Good tumor response | rs13181 | AC vs AA | No | 3 | 1.272 (0.855-1.892) | 0.236 | F | 0.14 | 0.932 | 0.00% |  |  |  |  |  |  |  |  |
|  |  |  | NA | 2 | 0.974 (0.550-1.725) | 0.929 | F | 0.49 | 0.483 | 0.00% |  |  |  |  |  |  |  |  |
|  |  | CC vs AA | No | 3 | 1.634 (0.800-3.340) | 0.178 | F | 0.18 | 0.916 | 0.00% |  |  |  |  |  |  |  |  |
|  |  |  | NA | 2 | 0.865 (0.372-2.011) | 0.736 | F | 1.14 | 0.285 | 12.60% |  |  |  |  |  |  |  |  |
|  |  | AC vs CC | No | 3 | 0.743 (0.385-1.435) | 0.377 | F | 0.02 | 0.991 | 0.00% |  |  |  |  |  |  |  |  |
|  |  |  | NA | 2 | 1.057 (0.462-2.419) | 0.895 | F | 0.29 | 0.588 | 0.00% |  |  |  |  |  |  |  |  |
|  |  | AC+CC vs AA | No | 4 | 1.572 (1.086-2.277) | 0.017 | F | 3.27 | 0.352 | 8.30% | 1.454 (0.994-2.125) | 0.054 | Katja et al. | 2.43 | F | 0.00 | 0.999 | 0.00% |
|  |  |  | NA | 2 | 0.909 (0.574-1.442) | 0.687 | F | 1.70 | 0.192 | 41.20% |  |  |  |  |  |  |  |  |
|  |  | A vs C | No | 3 | 0.739 (0.561-0.973) | 0.031 | F | 0.05 | 0.978 | 0.00% |  |  |  |  |  |  |  |  |
|  |  |  | NA | 2 | 1.196 (0.617-2.320) | 0.596 | R | 2.79 | 0.095 | 64.20% |  |  |  |  |  |  |  |  |
|  | rs11615 | TC vs TT | NA,T/C | 2 | 1.543 (0.993-2.397) | 0.054 | F | 0.05 | 0.819 | 0.00% |  |  |  |  |  |  |  |  |
|  |  | CC vs TT | NA,T/C | 2 | 2.731 (1.426-5.232) | 0.002 | F | 0.03 | 0.864 | 0.00% |  |  |  |  |  |  |  |  |
|  |  | TC vs CC | NA,T/C | 2 | 0.458 (0.240-0.876) | 0.018 | F | 0.43 | 0.513 | 0.00% |  |  |  |  |  |  |  |  |
|  |  | TC+CC vs TT | NA,T/C | 3 | 1.895 (1.309-2.742) | 0.001 | F | 2.47 | 0.291 | 18.90% | 2.035 (1.386-2.988) | <0.001 | Katja et al. | 4.15 | F | 0.65 | 0.418 | 0.00% |
|  |  | T vs C | NA,T/C | 2 | 0.520 (0.389-0.695) | <0.001 | F | 0.89 | 0.344 | 0.00% |  |  |  |  |  |  |  |  |
|  | rs1799793 | GA vs GG | NA | 3 | 1.345 (0.898-2.016) | 0.151 | F | 0.81 | 0.667 | 0.00% |  |  |  |  |  |  |  |  |
|  |  |  | No | 2 | 1.088 (0.630-1.879) | 0.762 | F | 1.51 | 0.219 | 33.90% |  |  |  |  |  |  |  |  |
|  |  | AA vs GG | NA | 3 | 1.932 (0.930-4.012) | 0.077 | F | 1.14 | 0.566 | 0.00% |  |  |  |  |  |  |  |  |
|  |  |  | No | 2 | 1.128 (0.307-4.146) | 0.856 | R | 3.15 | 0.076 | 68.20% |  |  |  |  |  |  |  |  |
|  |  | GA vs AA | NA | 3 | 0.600 (0.295-1.219) | 0.158 | F | 0.33 | 0.849 | 0.00% |  |  |  |  |  |  |  |  |
|  |  |  | No | 2 | 0.852 (0.404-1.798) | 0.674 | F | 0.62 | 0.430 | 0.00% |  |  |  |  |  |  |  |  |
|  |  | GA+AA vs GG | NA | 4 | 1.459 (1.033-2.060) | 0.032 | F | 1.98 | 0.576 | 0.00% |  |  |  |  |  |  |  |  |
|  |  |  | No | 2 | 1.043 (0.427-2.552) | 0.926 | R | 4.10 | 0.043 | 75.60% |  |  |  |  |  |  |  |  |
|  |  | G vs A | NA | 3 | 0.659 (0.495-0.876) | 0.004 | F | 0.96 | 0.620 | 0.00% |  |  |  |  |  |  |  |  |
|  |  |  | No | 2 | 0.957 (0.389-2.355) | 0.923 | R | 6.70 | 0.010 | 85.10% |  |  |  |  |  |  |  |  |
| Poor tumor response | rs13181 | AC vs AA | NA | 3 | 0.767 (0.530-1.110) | 0.159 | F | 0.15 | 0.928 | 0.00% |  |  |  |  |  |  |  |  |
|  |  |  | No | 2 | 1.036 (0.613-1.750) | 0.895 | F | 0.60 | 0.440 | 0.00% |  |  |  |  |  |  |  |  |
|  |  | CC vs AA | NA | 3 | 0.578 (0.307-1.089) | 0.090 | F | 0.08 | 0.962 | 0.00% |  |  |  |  |  |  |  |  |
|  |  |  | No | 2 | 1.171 (0.558-2.455) | 0.676 | F | 1.48 | 0.224 | 32.30% |  |  |  |  |  |  |  |  |
|  |  | AC vs CC | NA | 3 | 1.348 (0.698-2.605) | 0.374 | F | 0.01 | 0.993 | 0.00% |  |  |  |  |  |  |  |  |
|  |  |  | No | 2 | 0.946 (0.414-2.162) | 0.896 | F | 0.29 | 0.589 | 0.00% |  |  |  |  |  |  |  |  |
|  |  | AC+CC vs AA | NA | 4 | 0.704 (0.505-0.981) | 0.038 | F | 0.33 | 0.954 | 0.00% |  |  |  |  |  |  |  |  |
|  |  |  | No | 2 | 1.099 (0.691-1.750) | 0.690 | F | 1.59 | 0.207 | 37.20% |  |  |  |  |  |  |  |  |
|  |  | A vs C | NA | 3 | 1.347 (1.022-1.775) | 0.034 | F | 0.05 | 0.976 | 0.00% |  |  |  |  |  |  |  |  |
|  |  |  | No | 2 | 0.838 (0.435-1.613) | 0.596 | R | 2.72 | 0.099 | 63.30% |  |  |  |  |  |  |  |  |
|  | rs11615 | TC vs TT | NA,T/C | 2 | 0.591 (0.391-0.893) | 0.013 | F | 0.38 | 0.539 | 0.00% |  |  |  |  |  |  |  |  |
|  |  | CC vs TT | NA,T/C | 2 | 0.279 (0.147-0.530) | <0.001 | F | 1.04 | 0.307 | 4.10% |  |  |  |  |  |  |  |  |
|  |  | TC vs CC | NA,T/C | 2 | 2.174 (1.136-4.161) | 0.019 | F | 0.41 | 0.524 | 0.00% |  |  |  |  |  |  |  |  |
|  |  | TC+CC vs TT | NA,T/C | 2 | 1.615 (0.927-2.813) | 0.090 | F | 0.79 | 0.673 | 0.00% |  |  |  |  |  |  |  |  |
|  |  | T vs C | NA,T/C | 2 | 1.939 (1.453-2.589) | <0.001 | F | 0.90 | 0.342 | 0.00% |  |  |  |  |  |  |  |  |
|  | rs1799793 | GA vs GG | NA | 3 | 0.727 (0.500-1.056) | 0.094 | F | 0.47 | 0.789 | 0.00% |  |  |  |  |  |  |  |  |
|  |  |  | No | 2 | 0.925 (0.559-1.531) | 0.762 | F | 1.87 | 0.172 | 46.40% |  |  |  |  |  |  |  |  |
|  |  | AA vs GG | NA | 3 | 0.446 (0.229-0.869) | 0.018 | F | 0.94 | 0.626 | 0.00% |  |  |  |  |  |  |  |  |
|  |  |  | No | 2 | 0.886 (0.245-3.207) | 0.853 | R | 3.82 | 0.051 | 73.80% |  |  |  |  |  |  |  |  |
|  |  | GA vs AA | NA | 3 | 1.681 (0.822-3.435) | 0.154 | F | 0.32 | 0.851 | 0.00% |  |  |  |  |  |  |  |  |
|  |  |  | No | 2 | 1.176 (0.554-2.497) | 0.673 | F | 0.61 | 0.433 | 0.00% |  |  |  |  |  |  |  |  |
|  |  | GA+AA vs GG | NA | 4 | 0.689 (0.493-0.963) | 0.029 | F | 1.62 | 0.656 | 0.00% |  |  |  |  |  |  |  |  |
|  |  |  | No | 2 | 0.963 (0.393-2.362) | 0.935 | R | 4.19 | 0.041 | 76.10% |  |  |  |  |  |  |  |  |
|  |  | G vs A | NA | 3 | 1.520 (1.142-2.022) | 0.004 | F | 0.88 | 0.644 | 0.00% |  |  |  |  |  |  |  |  |
|  |  |  | No | 2 | 1.047 (0.432-2.538) | 0.920 | R | 6.48 | 0.011 | 84.60% |  |  |  |  |  |  |  |  |
