# Supplementary material for: Meta-analysis showing that ERCC1 polymorphism is predictive of osteosarcoma prognosis
Source: Oncotarget. 2017 Jul 19;8(37):62769–79. doi: 10.18632/oncotarget.19370 (PMC5617547; doi:10.18632/oncotarget.19370)
Supplement: Supplementary file 13 [file oncotarget-08-62769-s013.doc]

Supplementary Table 12: Subgroup analysis：Race

| Index | Locus | Genetic models | Subgroups | Number of studies | Test of association | | Test of heterogeneity | | | | Test of association after sensitivity analysis | | | | Test of heterogeneity after sensitivity analysis | | | |
| --- | --- | --- | --- | --- | --- | --- | --- | --- | --- | --- | --- | --- | --- | --- | --- | --- | --- | --- |
|  |  |  |  |  | HR/OR (95%CI) | P-value | Model | Chi-square | P-value | I² | OR (95%CI) | P-value | Study removed as heterogeneity source | Percentage of removed study(%) | Model | Chi-square | P-value | I^2^ |
| OS | rs13181 | AC vs AA | Asian | 6 | 0.892 (0.655-1.214) | 0.466 | F | 0.68 | 0.984 | 0.00% |  |  |  |  |  |  |  |  |
|  |  | CC vs AA | Asian | 6 | 0.806 (0.470-1.383) | 0.434 | F | 0.43 | 0.994 | 0.00% |  |  |  |  |  |  |  |  |
|  |  | AC vs CC | Asian | 6 | 1.132 (0.685-1.871) | 0.625 | F | 0.17 | 0.999 | 0.00% |  |  |  |  |  |  |  |  |
|  |  | AC+CC vs AA | Asian | 6 | 0.882 (0.674-1.155) | 0.361 | F | 1.04 | 0.960 | 0.00% |  |  |  |  |  |  |  |  |
|  |  |  | Caucasian | 2 | 0.749 (0.425-1.320) | 0.317 | F | 0.04 | 0.836 | 0.00% |  |  |  |  |  |  |  |  |
|  |  | A vs C | Asian | 6 | 1.146 (0.926-1.417) | 0.210 | F | 1.63 | 0.898 | 0.00% |  |  |  |  |  |  |  |  |
|  | rs11615 | TC vs TT | Asian,T/C | 3 | 0.692 (0.472-1.014) | 0.059 | F | 0.06 | 0.973 | 0.00% |  |  |  |  |  |  |  |  |
|  |  |  | Asian C/T | 4 | 1.178 (0.719-1.928) | 0.516 | F | 3.60 | 0.308 | 16.50% | 1.476 (0.855-2.550) | 0.162 | Sun Yongjian et al. | 6.36 | F | 0.08 | 0.959 | 0.00% |
|  |  | CC vs TT | Asian,T/C | 3 | 0.376 (0.205-0.688) | 0.002 | F | 0.16 | 0.923 | 0.00% |  |  |  |  |  |  |  |  |
|  |  |  | Asian C/T | 4 | 1.257 (0.471-3.356) | 0.648 | R | 12.91 | 0.005 | 76.80% | 2.035 (1.184-3.497) | 0.010 | Sun Yongjian et al. | 13.21 | F | 0.35 | 0.841 | 0.00% |
|  |  | TC vs CC | Asian,T/C | 3 | 1.781 (1.009-3.143) | 0.046 | F | 0.23 | 0.891 | 0.00% |  |  |  |  |  |  |  |  |
|  |  |  | Asian C/T | 4 | 0.795 (0.576-1.099) | 0.165 | F | 2.37 | 0.499 | 0.00% |  |  |  |  |  |  |  |  |
|  |  | TC+CC vs TT | Asian,T/C | 3 | 0.603 (0.433-0.840) | 0.003 | F | 0.15 | 0.927 | 0.00% |  |  |  |  |  |  |  |  |
|  |  |  | Asian,C/T | 4 | 1.151 (0.500-2.650) | 0.741 | R | 10.01 | 0.018 | 70.00% | 1.741 (1.039-2.918) | 0.035 | Sun Yongjian et al. | 9.28 | F | 0.18 | 0.915 | 0.00% |
|  |  |  | Caucasian,T/C | 2 | 0.998 (0.559-1.783) | 0.995 | F | 0.05 | 0.828 | 0.00% |  |  |  |  |  |  |  |  |
|  |  | T vs C | Asian,T/C | 3 | 1.619 (1.256-2.087) | <0.001 | F | 0.27 | 0.874 | 0.00% |  |  |  |  |  |  |  |  |
|  |  |  | Asian C/T | 4 | 0.934 (0.544-1.605) | 0.806 | R | 16.73 | 0.001 | 82.10% | 0.701 (0.547-0.897) | 0.005 | Sun Yongjian et al. | 11.68 | F | 0.43 | 0.808 | 0.00% |
|  | rs1799793 | GA vs GG | Asian | 6 | 0.928 (0.676-1.276) | 0.647 | F | 0.50 | 0.992 | 0.00% |  |  |  |  |  |  |  |  |
|  |  | AA vs GG | Asian | 6 | 0.560 (0.340-0.923) | 0.023 | F | 7.43 | 0.190 | 32.70% | 0.750 (0.423-1.330) | 0.325 | Wang MJ et al. | 25.42 | F | 3.23 | 0.520 | 0.00% |
|  |  | GA vs AA | Asian | 6 | 1.197 (0.721-1.988) | 0.488 | F | 3.89 | 0.566 | 0.00% |  |  |  |  |  |  |  |  |
|  |  | GA+AA vs GG | Asian | 6 | 0.862 (0.660-1.127) | 0.278 | F | 2.11 | 0.834 | 0.00% |  |  |  |  |  |  |  |  |
|  |  |  | Caucasian | 2 | 0.702 (0.383-1.286) | 0.252 | F | 1.77 | 0.184 | 43.40% |  |  |  |  |  |  |  |  |
|  |  | G vs A | Asian | 7 | 1.158 (0.936-1.434) | 0.177 | F | 4.35 | 0.500 | 0.00% |  |  |  |  |  |  |  |  |
|  | rs3212986 | CA vs CC | Asian | 3 | 0.864 (0.619-1.208) | 0.393 | F | 0.02 | 0.988 | 0.00% |  |  |  |  |  |  |  |  |
|  |  | AA vs CC | Asian | 3 | 0.686 (0.375-1.252) | 0.219 | F | 0.04 | 0.979 | 0.00% |  |  |  |  |  |  |  |  |
|  |  | CA vs AA | Asian | 3 | 1.360 (0.772-2.395) | 0.287 | F | 0.10 | 0.952 | 0.00% |  |  |  |  |  |  |  |  |
|  |  | CA+AA vs CC | Asian | 3 | 0.770 (0.564-1.050) | 0.098 | F | 0.09 | 0.957 | 0.00% |  |  |  |  |  |  |  |  |
|  |  |  | Caucasian | 2 | 1.250 (0.700-2.230) | 0.451 | F | 1.36 | 0.244 | 0.00% |  |  |  |  |  |  |  |  |
|  |  | C vs A | Asian | 3 | 1.272 (1.001-1.616) | 0.049 | F | 0.22 | 0.897 | 0.00% |  |  |  |  |  |  |  |  |
| Good tumor response | rs13181 | AC vs AA | Asian | 6 | 1.199 (0.895-1.605) | 0.224 | F | 1.33 | 0.931 | 0.00% |  |  |  |  |  |  |  |  |
|  |  | CC vs AA | Asian | 6 | 1.437 (0.873-2.366) | 0.154 | F | 4.07 | 0.540 | 0.00% |  |  |  |  |  |  |  |  |
|  |  | AC vs CC | Asian | 6 | 0.787 (0.489-1.267) | 0.324 | F | 1.38 | 0.927 | 0.00% |  |  |  |  |  |  |  |  |
|  |  | AC+CC vs AA | Asian | 6 | 1.210 (0.932-1.571) | 0.153 | F | 4.08 | 0.538 | 0.00% |  |  |  |  |  |  |  |  |
|  |  | A vs C | Asian | 6 | 0.834 (0.683-1.020) | 0.077 | F | 5.91 | 0.315 | 15.40% | 0.774 (0.627-0.955) | 0.017 | Sun Yongjian et al. | 9.19 | F | 0.53 | 0.970 | 0.00% |
|  | rs11615 | TC vs TT | Asian,T/C | 3 | 1.486 (1.032-2.138) | 0.033 | F | 0.14 | 0.932 | 0.00% |  |  |  |  |  |  |  |  |
|  |  |  | Asian C/T | 2 | 1.103 (0.537-2.264) | 0.789 | F | 0.85 | 0.356 | 0.00% |  |  |  |  |  |  |  |  |
|  |  | CC vs TT | Asian,T/C | 3 | 2.659 (1.554-4.548) | <0.001 | F | 0.05 | 0.975 | 0.00% |  |  |  |  |  |  |  |  |
|  |  |  | Asian C/T | 2 | 1.653 (0.378-7.227) | 0.504 | R | 5.05 | 0.025 | 80.20% |  |  |  |  |  |  |  |  |
|  |  | TC vs CC | Asian,T/C | 3 | 0.498 (0.296-0.839) | 0.009 | F | 0.61 | 0.739 | 0.00% |  |  |  |  |  |  |  |  |
|  |  |  | Asian C/T | 2 | 0.727 (0.322-1.642) | 0.443 | R | 2.67 | 0.102 | 62.50% |  |  |  |  |  |  |  |  |
|  |  | TC+CC vs TT | Asian,T/C | 3 | 1.885 (1.373-2.587) | <0.001 | F | 1.14 | 0.566 | 0.00% |  |  |  |  |  |  |  |  |
|  |  |  | Asian C/T | 2 | 1.531 (0.427-5.494) | 0.513 | R | 4.05 | 0.044 | 75.30% |  |  |  |  |  |  |  |  |
|  |  | T vs C | Asian,T/C | 3 | 0.554 (0.437-0.702) | <0.001 | F | 1.45 | 0.484 | 0.00% |  |  |  |  |  |  |  |  |
|  |  |  | Asian C/T | 2 | 0.660 (0.227-1.921) | 0.446 | R | 9.94 | 0.002 | 89.90% |  |  |  |  |  |  |  |  |
|  | rs1799793 | GA vs GG | Asian | 5 | 1.248 (0.901-1.727) | 0.182 | F | 2.70 | 0.610 | 0.00% |  |  |  |  |  |  |  |  |
|  |  | AA vs GG | Asian | 5 | 1.479 (0.881-2.482) | 0.139 | F | 5.32 | 0.256 | 24.80% | 2.014 (1.108-3.660) | 0.022 | Sun Yongjian et al. | 24.81 | F | 1.18 | 0.759 | 0.00% |
|  |  | GA vs AA | Asian | 5 | 0.708 (0.423-1.185) | 0.189 | F | 1.40 | 0.845 | 0.00% |  |  |  |  |  |  |  |  |
|  |  | GA+AA vs GG | Asian | 5 | 1.337 (1.014-1.763) | 0.039 | F | 6.46 | 0.167 | 38.10% | 1.550 (1.145-2.099) | 0.005 | Sun Yongjian et al. | 16.34 | F | 1.10 | 0.778 | 0.00% |
|  |  | G vs A | Asian | 5 | 0.752 (0.533-1.061) | 0.104 | R | 9.59 | 0.048 | 58.30% | 0.645 (0.506-0.821) | <0.001 | Sun Yongjian et al. | 19.15 | F | 1.04 | 0.793 | 0.00% |
|  | rs3212986 | AC+AA vs CC | Asian | 2 | 1.144 (0.817-1.603) | 0.434 | F | 0.00 | 0.980 | 0.00% |  |  |  |  |  |  |  |  |
| Poor tumor response | rs13181 | AC vs AA | Asian | 5 | 0.847 (0.626-1.146) | 0.283 | F | 1.59 | 0.811 | 0.00% |  |  |  |  |  |  |  |  |
|  |  | CC vs AA | Asian | 5 | 0.779 (0.481-1.260) | 0.309 | F | 3.57 | 0.467 | 0.00% |  |  |  |  |  |  |  |  |
|  |  | AC vs CC | Asian | 5 | 1.175 (0.702-1.967) | 0.540 | F | 0.74 | 0.947 | 0.00% |  |  |  |  |  |  |  |  |
|  |  | AC+CC vs AA | Asian | 5 | 0.837 (0.635-1.104) | 0.209 | F | 3.74 | 0.442 | 0.00% |  |  |  |  |  |  |  |  |
|  |  | A vs C | Asian | 5 | 1.171 (0.938-1.461) | 0.163 | F | 5.56 | 0.235 | 28.00% | 1.280 (1.012-1.619) | 0.039 | Sun Yongjian et al. | 12.93 | F | 0.52 | 0.914 | 0.00% |
|  | rs11615 | TC vs TT | Asian,T/C | 3 | 0.632 (0.450-0.889) | 0.008 | F | 0.70 | 0.704 | 0.00% |  |  |  |  |  |  |  |  |
|  |  |  | Asian C/T | 2 | 0.902 (0.440-1.848) | 0.777 | F | 0.83 | 0.362 | 0.00% |  |  |  |  |  |  |  |  |
|  |  | CC vs TT | Asian,T/C | 3 | 0.323 (0.193-0.540) | <0.001 | F | 1.60 | 0.449 | 0.00% |  |  |  |  |  |  |  |  |
|  |  |  | Asian C/T | 2 | 0.607 (0.139-2.639) | 0.505 | R | 5.11 | 0.024 | 80.40% |  |  |  |  | F | 0.00 | <0.001 | 0.00% |
|  |  | TC vs CC | Asian,T/C | 3 | 2.000 (1.188-3.368) | 0.009 | F | 0.58 | 0.747 | 0.00% |  |  |  |  |  |  |  |  |
|  |  |  | Asian C/T | 2 | 1.369 (0.605-3.094) | 0.451 | R | 2.62 | 0.106 | 61.80% |  |  |  |  | F | 0.00 | <0.001 | 0.00% |
|  |  | TC+CC vs TT | Asian,T/C | 3 | 1.677 (1.011-2.781) | 0.045 | F | 0.44 | 0.802 | 0.00% |  |  |  |  |  |  |  |  |
|  |  |  | Asian C/T | 2 | 1.071 (0.746-1.539) | 0.710 | F | 0.31 | 0.581 | 0.00% |  |  |  |  |  |  |  |  |
|  |  | T vs C | Asian,T/C | 3 | 1.814 (1.431-2.300) | <0.001 | F | 1.53 | 0.464 | 0.00% |  |  |  |  |  |  |  |  |
|  |  |  | Asian C/T | 2 | 1.515 (0.526-4.365) | 0.441 | R | 9.84 | 0.002 | 89.80% |  |  |  |  | F | 0.00 | <0.001 | 0.00% |
|  | rs1799793 | GA vs GG | Asian | 5 | 0.792 (0.586-1.069) | 0.128 | F | 2.91 | 0.574 | 0.00% |  |  |  |  |  |  |  |  |
|  |  | AA vs GG | Asian | 5 | 0.601 (0.322-1.121) | 0.109 | R | 6.79 | 0.147 | 41.10% | 0.450 (0.262-0.775) | 0.004 | Sun Yongjian et al. | 25.14 | F | 0.94 | 0.816 | 0.00% |
|  |  | GA vs AA | Asian | 5 | 1.419 (0.845-2.383) | 0.186 | F | 1.39 | 0.845 | 0.00% |  |  |  |  |  |  |  |  |
|  |  | GA+AA vs GG | Asian | 5 | 0.748 (0.570-0.982) | 0.037 | F | 6.33 | 0.176 | 36.80% | 0.648 (0.481-0.872) | 0.004 | Sun Yongjian et al. | 16.57 | F | 0.87 | 0.832 | 0.00% |
|  |  | G vs A | Asian | 5 | 1.330 (0.946-1.869) | 0.101 | R | 9.36 | 0.053 | 57.30% | 1.550 (1.216-1.975) | <0.001 | Sun Yongjian et al. | 19.26 | F | 0.94 | 0.815 | 0.00% |
|  | rs3212986 | CA vs CC | Asian | 2 | 0.803 (0.541-1.191) | 0.275 | F | 0.11 | 0.741 | 0.00% |  |  |  |  |  |  |  |  |
|  |  | AA vs CC | Asian | 2 | 0.415 (0.194-0.889) | 0.024 | F | 0.30 | 0.585 | 0.00% |  |  |  |  |  |  |  |  |
|  |  | CA vs AA | Asian | 2 | 1.971 (0.908-4.280) | 0.086 | F | 0.14 | 0.704 | 0.00% |  |  |  |  |  |  |  |  |
|  |  | CA+AA vs CC | Asian | 2 | 0.723 (0.496-1.054) | 0.092 | F | 0.15 | 0.701 | 0.00% |  |  |  |  |  |  |  |  |
|  |  | C vs A | Asian | 2 | 1.376 (1.026-1.845) | 0.033 | F | 0.10 | 0.755 | 0.00% |  |  |  |  |  |  |  |  |
