# Supplementary material for: Meta-analysis showing that ERCC1 polymorphism is predictive of osteosarcoma prognosis
Source: Oncotarget. 2017 Jul 19;8(37):62769–79. doi: 10.18632/oncotarget.19370 (PMC5617547; doi:10.18632/oncotarget.19370)
Supplement: Supplementary file 14 [file oncotarget-08-62769-s014.doc]

Supplementary Table 13: Subgroup analysis：Treatment

| Index | Locus | Genetic models | Subgroups | Number of studies | Test of association | | Test of heterogeneity | | | | Test of association after sensitivity analysis | | | | Test of heterogeneity after sensitivity analysis | | | |
| --- | --- | --- | --- | --- | --- | --- | --- | --- | --- | --- | --- | --- | --- | --- | --- | --- | --- | --- |
|  |  |  |  |  | HR/OR (95%CI) | P-value | Model | Chi-square | P-value | I² | OR (95%CI) | P-value | Study removed as heterogeneity source | Percentage of removed study(%) | Model | Chi-square | P-value | I^2^ |
| OS | rs13181 | AC vs AA | Cisplatin-based therapy | 5 | 0.880 (0.635-1.220) | 0.443 | F | 0.29 | 0.990 | 0.00% |  |  |  |  |  |  |  |  |
|  |  |  | No preoperative chemotherapy | 2 | 0.830 (0.452-1.521) | 0.546 | F | 0.58 | 0.446 | 0.00% |  |  |  |  |  |  |  |  |
|  |  | CC vs AA | Cisplatin-based therapy | 5 | 0.716 (0.399-1.284) | 0.262 | F | 0.70 | 0.951 | 0.00% |  |  |  |  |  |  |  |  |
|  |  |  | No preoperative chemotherapy | 2 | 0.850 (0.304-2.377) | 0.756 | F | 0.33 | 0.564 | 0.00% |  |  |  |  |  |  |  |  |
|  |  | AC vs CC | Cisplatin-based therapy | 5 | 1.219 (0.711-2.090) | 0.471 | F | 0.49 | 0.974 | 0.00% |  |  |  |  |  |  |  |  |
|  |  |  | No preoperative chemotherapy | 2 | 1.072 (0.376-3.055) | 0.896 | F | 0.00 | 0.952 | 0.00% |  |  |  |  |  |  |  |  |
|  |  | AC+CC vs AA | Cisplatin-based therapy | 6 | 0.849 (0.646-1.116) | 0.242 | F | 0.33 | 0.997 | 0.00% |  |  |  |  |  |  |  |  |
|  |  |  | No preoperative chemotherapy | 2 | 0.881 (0.516-1.503) | 0.641 | F | 0.99 | 0.319 | 0.00% |  |  |  |  |  |  |  |  |
|  |  | A vs C | Cisplatin-based therapy | 5 | 1.190 (0.947-1.495) | 0.136 | F | 0.84 | 0.933 | 0.00% |  |  |  |  |  |  |  |  |
|  |  |  | No preoperative chemotherapy | 2 | 1.099 (0.714-1.693) | 0.667 | F | 1.05 | 0.306 | 4.60% |  |  |  |  |  |  |  |  |
|  | rs11615 | TC vs TT | No preoperative chemotherapy ,C/T | 2 | 1.413 (0.757-2.634) | 0.835 | R | 2.73 | 0.099 | 63.30% |  |  |  |  |  |  |  |  |
|  |  |  | Cisplatin-based therapy,T/C | 4 | 0.695 (0.488-0.990) | 0.044 | F | 0.06 | 0.996 | 0.00% |  |  |  |  |  |  |  |  |
|  |  |  | Cisplatin-based therapy C/T | 2 | 1.413 (0.757-2.634) | 0.277 | F | 0.00 | 0.983 | 0.00% |  |  |  |  |  |  |  |  |
|  |  | CC vs TT | No preoperative chemotherapy ,C/T | 2 | 0.857 (0.103-7.108) | 0.886 | R | 8.32 | 0.004 | 88.00% |  |  |  |  |  |  |  |  |
|  |  |  | Cisplatin-based therapy,T/C | 4 | 0.539 (0.324-0.897) | 0.017 | R | 4.89 | 0.180 | 38.70% | 0.376 (0.205-0.688) | 0.002 | Paola et al. | 13.12 | F | 0.16 | 0.923 | 0.00% |
|  |  |  | Cisplatin-based therapy C/T | 2 | 1.899 (1.028-3.506) | 0.041 | F | 0.12 | 0.725 | 0.00% |  |  |  |  |  |  |  |  |
|  |  | TC vs CC | No preoperative chemotherapy ,C/T | 2 | 0.886 (0.508-1.546) | 0.670 | F | 1.90 | 0.168 | 47.30% |  |  |  |  |  |  |  |  |
|  |  |  | Cisplatin-based therapy,T/C | 4 | 1.280 (0.777-2.108) | 0.332 | F | 5.91 | 0.116 | 49.20% | 1.781 (1.009-3.143) | 0.046 | Paola et al. | 6.75 | F | 0.23 | 0.891 | 0.00% |
|  |  |  | Cisplatin-based therapy C/T | 2 | 0.753 (0.506-1.119) | 0.161 | F | 0.26 | 0.611 | 0.00% |  |  |  |  |  |  |  |  |
|  |  | TC+CC vs TT | No preoperative chemotherapy ,C/T | 2 | 0.809 (0.133-4.933) | 0.818 | R | 6.53 | 0.011 | 84.70% |  |  |  |  |  |  |  |  |
|  |  |  | Cisplatin-based therapy,T/C | 5 | 0.683 (0.512-0.910) | 0.009 | F | 2.38 | 0.666 | 0.00% |  |  |  |  |  |  |  |  |
|  |  |  | Cisplatin-based therapy C/T | 2 | 1.652 (0.919-2.969) | 0.093 | F | 0.04 | 0.842 | 0.00% |  |  |  |  |  |  |  |  |
|  |  | T vs C | No preoperative chemotherapy ,C/T | 2 | 1.231 (0.338-4.477) | 0.753 | R | 12.37 | <0.001 | 91.90% |  |  |  |  |  |  |  |  |
|  |  |  | Cisplatin-based therapy,T/C | 4 | 1.455 (1.151-1.839) | 0.002 | F | 4.83 | 0.185 | 37.80% | 1.619 (1.256-2.087) | <0.001 | Paola et al. | 7.12 | F | 0.27 | 0.874 | 0.00% |
|  |  |  | Cisplatin-based therapy C/T | 2 | 0.722 (0.543-0.960) | 0.025 | F | 0.26 | 0.613 | 0.00% |  |  |  |  |  |  |  |  |
|  | rs1799793 | GA vs GG | No preoperative chemotherapy | 2 | 1.079 (0.568-2.051) | 0.816 | F | 0.16 | 0.687 | 0.00% |  |  |  |  |  |  |  |  |
|  |  |  | Cisplatin-based therapy | 5 | 0.824 (0.585-1.161) | 0.269 | F | 1.21 | 0.877 | 0.00% |  |  |  |  |  |  |  |  |
|  |  | AA vs GG | No preoperative chemotherapy | 2 | 0.539 (0.089-3.283) | 0.503 | R | 5.33 | 0.021 | 81.30% |  |  |  |  |  |  |  |  |
|  |  |  | Cisplatin-based therapy | 5 | 0.595 (0.327-1.081) | 0.088 | F | 1.97 | 0.742 | 0.00% |  |  |  |  |  |  |  |  |
|  |  | GA vs AA | No preoperative chemotherapy | 2 | 1.358 (0.549-3.362) | 0.508 | F | 0.24 | 0.625 | 0.00% |  |  |  |  |  |  |  |  |
|  |  |  | Cisplatin-based therapy | 5 | 1.420 (0.798-2.528) | 0.233 | F | 2.29 | 0.683 | 0.00% |  |  |  |  |  |  |  |  |
|  |  | GA+AA vs GG | No preoperative chemotherapy | 2 | 1.040 (0.630-1.718) | 0.877 | F | 0.69 | 0.405 | 0.00% |  |  |  |  |  |  |  |  |
|  |  |  | Cisplatin-based therapy | 7 | 0.778 (0.588-1.030) | 0.079 | F | 2.57 | 0.765 | 0.00% |  |  |  |  |  |  |  |  |
|  |  | G vs A | No preoperative chemotherapy | 2 | 0.894 (0.599-1.333) | 0.582 | F | 0.47 | 0.491 | 0.00% |  |  |  |  |  |  |  |  |
|  |  |  | Cisplatin-based therapy | 6 | 1.340 (1.054-1.703) | 0.017 | F | 2.77 | 0.597 | 0.00% |  |  |  |  |  |  |  |  |
| Good tumor response | rs13181 | AC vs AA | Cisplatin-based therapy | 5 | 1.196 (0.961-1.488) | 0.110 | F | 1.47 | 0.832 | 0.00% |  |  |  |  |  |  |  |  |
|  |  | CC vs AA | Cisplatin-based therapy | 5 | 1.682 (0.988-2.861) | 0.055 | F | 1.30 | 0.861 | 0.00% |  |  |  |  |  |  |  |  |
|  |  | AC vs CC | Cisplatin-based therapy | 5 | 0.733 (0.443-1.211) | 0.225 | F | 0.60 | 0.963 | 0.00% |  |  |  |  |  |  |  |  |
|  |  | AC+CC vs AA | Cisplatin-based therapy | 6 | 1.380 (1.051-1.813) | 0.021 | F | 4.36 | 0.499 | 0.00% |  |  |  |  |  |  |  |  |
|  |  | A vs C | Cisplatin-based therapy | 5 | 0.774 (0.627-0.955) | 0.017 | F | 0.53 | 0.970 | 0.00% |  |  |  |  |  |  |  |  |
|  | rs11615 | TC vs TT | Cisplatin-based therapy,T/C | 3 | 1.486 (1.032-2.138) | 0.033 | F | 0.14 | 0.932 | 0.00% |  |  |  |  |  |  |  |  |
|  |  | CC vs TT | Cisplatin-based therapy,T/C | 3 | 2.659 (1.554-4.548) | <0.001 | F | 0.05 | 0.975 | 0.00% |  |  |  |  |  |  |  |  |
|  |  | TC vs CC | Cisplatin-based therapy,T/C | 3 | 0.498 (0.296-0.839) | 0.009 | F | 0.61 | 0.739 | 0.00% |  |  |  |  |  |  |  |  |
|  |  | TC+CC vs TT | Cisplatin-based therapy,T/C | 4 | 1.800 (1.322-2.450) | <0.001 | F | 2.71 | 0.439 | 0.00% |  |  |  |  |  |  |  |  |
|  |  | T vs C | Cisplatin-based therapy,T/C | 3 | 0.554 (0.437-0.702) | <0.001 | F | 1.45 | 0.484 | 0.00% |  |  |  |  |  |  |  |  |
|  | rs1799793 | GA vs GG | Cisplatin-based therapy | 4 | 1.364 (0.962-1.934) | 0.082 | F | 0.83 | 0.843 | 0.00% |  |  |  |  |  |  |  |  |
|  |  | AA vs GG | Cisplatin-based therapy | 4 | 2.014 (1.108-3.660) | 0.022 | F | 1.18 | 0.759 | 0.00% |  |  |  |  |  |  |  |  |
|  |  | GA vs AA | Cisplatin-based therapy | 4 | 0.616 (0.345-1.100) | 0.101 | F | 0.34 | 0.952 | 0.00% |  |  |  |  |  |  |  |  |
|  |  | GA+AA vs GG | Cisplatin-based therapy | 5 | 1.500 (1.115-2.017) | 0.007 | F | 2.08 | 0.722 | 0.00% |  |  |  |  |  |  |  |  |
|  |  | G vs A | Cisplatin-based therapy | 4 | 0.645 (0.506-0.821) | <0.001 | F | 1.04 | 0.793 | 0.00% |  |  |  |  |  |  |  |  |
| Poor tumor response | rs13181 | AC vs AA | Cisplatin-based therapy | 5 | 1.031 (0.613-1.733) | 0.908 | R | 10.52 | 0.033 | 62.00% | 0.802 (0.584-1.102) | 0.174 | D Carolina et al. | 10.63 | F | 0.37 | 0.947 | 0.00% |
|  |  | CC vs AA | Cisplatin-based therapy | 5 | 0.758 (0.461-1.245) | 0.131 | F | 3.96 | 0.411 | 0.00% |  |  |  |  |  |  |  |  |
|  |  | AC vs CC | Cisplatin-based therapy | 5 | 1.663 (1.019-2.715) | 0.042 | F | 4.83 | 0.305 | 17.10% | 1.267 (0.732-2.192) | 0.398 | D Carolina et al. | 18.22 | F | 0.13 | 0.988 | 0.00% |
|  |  | AC+CC vs AA | Cisplatin-based therapy | 5 | 0.747 (0.561-0.995) | 0.046 | F | 0.82 | 0.936 | 0.00% |  |  |  |  |  |  |  |  |
|  |  | A vs C | Cisplatin-based therapy | 5 | 1.280 (1.012-1.619) | 0.039 | F | 0.52 | 0.914 | 0.00% |  |  |  |  |  |  |  |  |
|  | rs11615 | TC vs TT | Cisplatin-based therapy,T/C | 4 | 0.673 (0.485-0.933) | 0.017 | F | 2.29 | 0.514 | 0.00% |  |  |  |  |  |  |  |  |
|  |  | CC vs TT | Cisplatin-based therapy,T/C | 4 | 0.443 (0.208-0.944) | 0.035 | R | 7.23 | 0.065 | 58.50% | 0.323 (0.193-0.540) | <0.001 | D Carolina et al. | 12.49 | F | 1.60 | 0.449 | 0.00% |
|  |  | TC vs CC | Cisplatin-based therapy,T/C | 4 | 1.893 (1.185-3.026) | 0.008 | F | 0.81 | 0.848 | 0.00% |  |  |  |  |  |  |  |  |
|  |  | TC+CC vs TT | Cisplatin-based therapy,T/C | 4 | 1.573 (0.990-2.500) | 0.055 | F | 0.82 | 0.845 | 0.00% |  |  |  |  |  |  |  |  |
|  |  | T vs C | Cisplatin-based therapy,T/C | 4 | 1.814 (1.431-2.300) | <0.001 | F | 1.53 | 0.464 | 0.00% |  |  |  |  |  |  |  |  |
|  | rs1799793 | GA vs GG | Cisplatin-based therapy | 5 | 0.769 (0.565-1.048) | 0.096 | F | 2.31 | 0.679 | 0.00% |  |  |  |  |  |  |  |  |
|  |  | AA vs GG | Cisplatin-based therapy | 5 | 0.554 (0.331 0.926) | 0.024 | F | 6.47 | 0.167 | 38.20% | 0.450 (0.262-0.775) | 0.004 | D Carolina et al. | 7.73 | F | 0.94 | 0.816 | 0.00% |
|  |  | GA vs AA | Cisplatin-based therapy | 5 | 1.675 (1.014-2.766) | 0.044 | F | 0.37 | 0.985 | 0.00% |  |  |  |  |  |  |  |  |
|  |  | GA+AA vs GG | Cisplatin-based therapy | 5 | 0.671 (0.503-0.895) | 0.007 | F | 1.71 | 0.788 | 0.00% |  |  |  |  |  |  |  |  |
|  |  | G VS A | Cisplatin-based therapy | 5 | 1.550 (1.216-1.975) | <0.001 | F | 0.94 | 0.815 | 0.00% |  |  |  |  |  |  |  |  |
