# Supplementary material for: Meta-analysis showing that ERCC1 polymorphism is predictive of osteosarcoma prognosis
Source: Oncotarget. 2017 Jul 19;8(37):62769–79. doi: 10.18632/oncotarget.19370 (PMC5617547; doi:10.18632/oncotarget.19370)
Supplement: Supplementary file 15 [file oncotarget-08-62769-s015.doc]

Supplementary Table 14: Literature Strategy

| #1 ERCC OR ERCC1 OR ERCC2 OR “Excision-repair cross-complementing complementation group 1” OR “Excision-repair cross-complementing complementation group 2” OR “Xeroderma pigmentosum group D” OR XPD |
| --- |
| #2 Osteosarcoma OR “Bone malignant tumor” OR “Bone sarcoma” |
| #3 Outcome OR Prognosis OR Survival OR Response |
| #4 Polymorphism OR Mutation OR Variant |
| #5 #1 AND #2 |
| #6 #3 AND #4 |
| #7 #5 AND #6 |
|  |
