# Supplementary material for: Meta-analysis showing that ERCC1 polymorphism is predictive of osteosarcoma prognosis
Source: Oncotarget. 2017 Jul 19;8(37):62769–79. doi: 10.18632/oncotarget.19370 (PMC5617547; doi:10.18632/oncotarget.19370)
Supplement: Supplementary file 16 [file oncotarget-08-62769-s016.doc]

| **Section/topic** | **#** | **Checklist item** | **Reported on page #** |
| --- | --- | --- | --- |
| **TITLE** | | |  |
| Title | 1 | ERCC polymorphisms and osteosarcoma prognosis： a systematic review and meta-analysis | Title section |
| **ABSTRACT** | | |  |
| Structured summary | 2 | To investigate correlations between excision repair cross-complementation group 1 (ERCC1) and 2 (ERCC2) polymorphisms and osteosarcoma prognosis, we conducted a meta-analysis of studies published through October 2016. Studies were identified in the PubMed, ScienceDirect, Springer, and Web of Science databases using preferred reporting items for systematic reviews and meta-analyses (PRISMA). Odds ratios (ORs) or hazard ratios (HRs) and their 95% confidence intervals (CIs) for overall survival (OS), tumor response (TR), and event-free survival (EFS) were estimated. Eleven studies that scored >6 on the Newcastle-Ottawa Scale (NOS) and investigated four SNPs reportedly associated with osteosarcoma prognosis (ERCC1 rs11615 and rs3212986, and ERCC2 rs13181 and rs1799793) were included in our meta-analysis. We found that only one SNP, ERCC1 rs11615, correlated with improved OS and TR. The HR of T vs. C for OS was 1.455 (T/C, 95%CI=1.151–1.839, P=0.002, I2=37.80%). The OR of T vs. C for good TR was 0.554 (T/C, 95%CI=0.437–0.702, P<0.001, I2=0%). Few significant outcomes were observed in subgroup analyses stratified based on study characteristics with adjustments for potential confounders. Our results suggest that ERCC1 rs11615 CC is associated with a better clinical outcome. This suggests rs11615 may be a useful genetic marker for predicting osteosarcoma prognosis. | Abstract section |
| **INTRODUCTION** | | |  |
| Rationale | 3 | Osteosarcoma is one of the most common and aggressive malignant bone tumors, primarily occurring during adolescent growth and in the elderly. Osteosarcoma incidence in adolescents is relatively consistent globally and ranges from 3–4.5 cases per million persons per year. Although osteosarcoma treatment options have improved, patient prognosis remains poor.  DNA repair is critical for maintaining DNA stability and integrity, and cell function. The nucleotide excision repair (NER) pathway is responsible for recognizing and excising DNA lesions. Excision repair cross-complementation group 1 (ERCC1) and 2 (ERCC2), located in 19q13.3, are key rate-limiting enzymes in the NER process. ERCC1 and xeroderma pigmentosum group F (XPF) form a heterodimer to catalyze 5’–3’ incisions, while ERCC2 exhibits ATP-dependent DNA helicase activity, inducing apoptosis and basal transcription. Therefore, ERCC polymorphisms may impact DNA repair and cancer development and progression. | Introduction section  Paragraph 1 and 2 |
| Objectives | 4 | To investigate the correlation between ERCC polymorphisms and osteosarcoma prognosis, a systematic review and meta-analysis was conducted based on PRISMA statement. | Introduction section  Paragraph 2 |
| **METHODS** | | |  |
| Protocol and registration | 5 | Not available | NA |
| Eligibility criteria | 6 | Studies included in our analysis met the following criteria: (1) studies were limited to the published research concerning osteosarcoma prognosis and ERCC polymorphisms; (2) patients in the original studies must have been diagnosed with osteosarcoma via imaging, pathology, or the latest clinical diagnostic criteria, and genotyping was performed using valid molecular techniques; and (3) detailed patient data and the number of participants with distinct genotypes were published in the studies so that the adjusted or crude OR/HR value could be calculated. | Method section  Study selection |
| Information sources | 7 | We comprehensively searched for potential references from the PubMed, ScienceDirect, Springer, and Web of Science databases using key words such as “ERCC,” “osteosarcoma,” “outcome,” etc. through October, 2016. | Method section  Literature search strategy |
| Search | 8 | Literature strategy was shown as following:  (ERCC OR ERCC1 OR ERCC2 OR “Excision-repair cross-complementing complementation group 1” OR “Excision-repair cross-complementing complementation group 2” OR “Xeroderma pigmentosum group D” OR XPD) AND (Osteosarcoma OR “Bone malignant tumor” OR “Bone sarcoma”) AND (Outcome OR Prognosis OR Survival OR Response) AND (Polymorphism OR Mutation) | Method section  Literature search strategy and Supplemental material |
| Study selection | 9 | Studies included in our analysis met the following criteria: (1) studies were limited to the published research concerning osteosarcoma prognosis and ERCC polymorphisms; (2) patients in the original studies must have been diagnosed with osteosarcoma via imaging, pathology, or the latest clinical diagnostic criteria, and genotyping was performed using valid molecular techniques; and (3) detailed patient data and the number of participants with distinct genotypes were published in the studies so that the adjusted or crude OR/HR value could be calculated. | Method section  Study selection |
| Data collection process | 10 | Data were extracted by two investigators independently and double-checked by a third investigator. Inconsistent data were addressed by open discussion and consensus was achieved via input from a senior investigator. | Method section  Data extraction |
| Data items | 11 | Data of fundamental and clinical characteristics were extracted and some variables were used in subgroup analysises to eliminate impacts of potential confounders and further investigate potential correlations. Original outcomes were extracted, among which adjusted ORs were firstly used in our meta-analysis otherwise crude ORs were used. Crude ORs were employed with the hope of enlarging size of studies, but might be a cause of heterogeneity. | Method section  and  Table.1 and 2 |
| Risk of bias in individual studies | 12 | Study type of our included studies were mostly case-control study, and clinical diagnosis of patients and methodology were valid. Bias risks might mainly be in differences of characteristics of patients. | Method section and Table 1 and 2 |
| Summary measures | 13 | Stata 11.0 software was used to perform this meta-analysis. A Hardy-Weinberg equilibrium (HWE) test was performed using extracted data, and P<0.05 was considered a significant imbalance. Adjusted HRs/ORs from confounders (age, metastasis, etc.) in every model were used in our meta-analysis; otherwise, crude HRs/ORs calculated by Revman 5.3 software were used. Pooled data had low heterogeneity if P>0.1 and I2<50%. In these cases, a fixed effects model was used; otherwise, a random effects model was used. Statistical analysis of pooled data was performed using models as follows: 12 versus 11, 22 versus 11, 12 versus 22, 12+22 versus 11, 1 versus 2 (1 represented the wild allele and 2 represented the mutated allele).  Tumor-related indexes, including TR, OS, and EFS, were assessed if the number of studies containing usable data was greater than 3 . Comparisons between groups were two-sided and P<0.05 was considered significant. | Method section  Statistical analysis |
| Synthesis of results | 14 | Heterogeneity was measured by chi-square and I^2^. Pooled data was considered as in a good homogeneity if P>0.1 and fixed effects model was used, otherwise random effects model was used. Sensitivity analysis was performed to detect the source of heterogeneity. | Method section  Statistical analysis and  subgroup analysis and heterogeneity |

Page 1 of 2

| **Section/topic** | **#** | **Checklist item** | **Reported on page #** |
| --- | --- | --- | --- |
| Risk of bias across studies | 15 | Begg’s and Egger’s test were used to evaluate the potential publication bias. Besides, funnel plots were shown to evaluate publication bias visually. | Method section  Publication bias |
| Additional analyses | 16 | Not available | NA |
| **RESULTS** | | |  |
| Study selection | 17 | The combined search yielded 556 potentially relevant references. After screening, eleven studies met our criteria and were included in the meta-analysis. | Results section  Reference Search and Fig.1 |
| Study characteristics | 18 | Fundamental and clinical characteristics of included studies were shown in Table.1 and 2 | Results section  Data extraction  Table.1 and 2 |
| Risk of bias within studies | 19 | Newcastle-Ottawa Scale (NOS) assessments are shown in Table 3. With the aim of evaluating studies more precisely, we added additional clauses to some items. All studies received scores ≥6. | Results section  Data extraction and reference assessment |
| Results of individual studies | 20 | Data extraction was shown in Table. 2, results of meta-analysis are shown in Table.4 and Table.5. Results of subgroup analysis were shown in Supplemental material. | Results section  Statistical analysis  Table 2, 4 and 5 |
| Synthesis of results | 21 | Only ERCC1 rs11615 was observed a significant outcome as positive OS and TR. Results of meta-analysis are shown in Table.4 and Table.5 | Results section  Statistical analysis  Table 4 and 5 |
| Risk of bias across studies | 22 | Begg's tests did not show significance for any SNPs with any outcomes, and Egger's test found significance for TR only in some models (Fig 5). This may have been because TR was not estimated in some studies, and thus sample size was low. | Results section  Statistical analysis  Table 5. |
| Additional analysis | 23 | NA | NA |
| **DISCUSSION** | | |  |
| Summary of evidence | 24 | Only ERCC1 rs11615 was associated with improved patient OS and TR. No outcomes were associated with ERCC2 rs13181 or rs1799793, or ERCC1 rs3212986. | Discuss section  The first paragraph |
| Limitations | 25 | Our study had limitations. First, numerous factors were involved in tumor prognosis. Single SNPs are unlikely to be major factors affecting tumor prognosis. Gene-environment and gene-gene interactions, and the combined effects of multiple SNPs in several genes are more effective outcome predictors than single SNPs alone [77-78]. However, we found no relevant gene interaction data in the included studies. Second, primary data was not available in some models. Thus, adjusted ORs were merged with crude ORs, and heterogeneity under such circumstances was high. We were unable to determine the sources of heterogeneity for some studies, such as that of Sun, et al. In addition, in ERCC1 rs11615, discrepancies between T/C and C/T remain unsettled [31 ]. While rs11615 T/C was correct according to PubMed dbSNP, we could not confirm this with high confidence from our study. In our analysis, T/C and C/T study outcomes were contradictory, with high heterogeneity. We performed a subgroup analysis based on T/C and C/T to reduce heterogeneity, but our sample size was severely limited. Further analyses are necessary to rectify this confusing issue. | Discuss section  The seventh paragraph |
| Conclusions | 26 | In conclusion, our meta-analysis indicated that ERCC1 rs11615 is associated with improved osteosarcoma prognosis. Additional studies with larger sample sizes are needed to more precisely estimate the correlation between ERCC polymorphisms and osteosarcoma prognosis. | Discuss section  Last paragraph |
| **FUNDING** | | |  |
| Funding | 27 | NA. | NA |

*From:*  Moher D, Liberati A, Tetzlaff J, Altman DG, The PRISMA Group (2009). Preferred Reporting Items for Systematic Reviews and Meta-Analyses: The PRISMA Statement. PLoS Med 6(7): e1000097. doi:10.1371/journal.pmed1000097

For more information, visit: **www.prisma-statement.org**.

Page 2 of 2
