# Supplementary material for: Meta-analysis showing that ERCC1 polymorphism is predictive of osteosarcoma prognosis
Source: Oncotarget. 2017 Jul 19;8(37):62769–79. doi: 10.18632/oncotarget.19370 (PMC5617547; doi:10.18632/oncotarget.19370)
Supplement: Supplementary file 17 [file oncotarget-08-62769-s017.doc]

Supplementary Table 16: Websites of dbSNP of SNPs:

| rs13181: <https://www.ncbi.nlm.nih.gov/projects/SNP/snp_ref.cgi?rs=13181> |
| --- |
| rs11615: <http://www.ncbi.nlm.nih.gov/projects/SNP/snp_ref.cgi?rs=11615> |
| rs3212986: <https://www.ncbi.nlm.nih.gov/projects/SNP/snp_ref.cgi?rs=3212986> |
| rs1799793: <https://www.ncbi.nlm.nih.gov/SNP/snp_ref.cgi?rs=1799793> |
|  |
